# Supplementary material for: Relationship between dietary factors and the number of altered metabolic syndrome components in Chinese adults: a cross-sectional study using data from the China Health and Nutrition Survey
Source: BMJ Open. 2017 May 29;7(5):e014911. doi: 10.1136/bmjopen-2016-014911 (PMC5729973; doi:10.1136/bmjopen-2016-014911)
Supplement: Supplementary data [file bmjopen-2016-014911supp001.pdf]

Supplemental Table 1. The full list of food items and examples for each food group using by a household inventory

No	Food group	Examples of food items
1	Rice	Japonica rice, glutinous rice
2	Flour	Steamed bread, noodles
3	Coarse cereals	Corn, barley, millet
4	Tubers	Potato, sweet potato
5	Beans	Beans, mung beans,
6	Vegetables,	Tomato, cabbage, white radish, mungbean sprout
7	Fruits	Apples,watermelons
8	Nuts	Peanuts, melon seeds
9	Pork	
10	Other livestock meat	Cattle, sheep
11	Poultry meat	Chicken, duck, goose
12	Abdominal organs	Pork liver, pork heart
13	Fish/seafood	Fish, shrimp, crab
14	Milk and dairy products	Milk, yogurt
15	Eggs	Eggs, duck eggs
16	Vegetable oil	
17	Fat oil	
18	Snacks and dessert	Bean jelly, cold noodles, cakes
19	Fans/starch and sugar	Starch, sugar
20	Condiments	Salt, soy sauce, sauce

Supplemental Table 2. Energy and multivariate adjusted[a,b] mean nutrient intake according to the number of metabolic syndrome (MetS)components in men (N=2800)

	Number of MetS components					
	0	1	2	3−5	P^b	Trend P
n，%	866（30.9%）	832（29.7%）	618（22.1%）	484（17.3%）		
Energy	2349.9±20.8	2317.5±21.3	2296.7±24.6	2269.6±27.8	0.109	0.016
Nutrients[a]						
VitA(μg RE)	445.8±17.76	443.9±17.58	440.4±19.83	451.1±22.29	0.986	0.886
$VitB_1$(mg)	1.0±0.01	1.0±0.01	1.1±0.02	1.0±0.02	0.688	0.656
$VitB_2$(mg)	0.8±0.01	0.8±0.01	0.8±0.01	0.8±0.01	0.777	0.510
$VitB_3$(mg)	15.1±0.20	15.4±0.19	15.6±0.22	15.6±0.25	0.262	0.101
K(mg)	1750.2±16.31	1740.1±16.15	1727.0±18.25	1753.1±20.47	0.711	0.955
Zn(mg)	11.7±0.10	11.8±0.10	11.9±0.11	12.0±0.13	0.273	0.053
Se(μg)	48.1±0.9	48.2±0.89	48.8±1.00	49.2±1.13	0.858	0.402
P(mg)	1060.9±8.07	1063.2±7.99	1063.4±9.01	1067.7±10.13	0.959	0.596
Mn(mg)	7.0±0.19	7.0±0.19	6.7±0.21	7.3±0.24	0.409	0.701
Cu(mg)	2.1±0.03	2.2±0.03	2.1±0.04	2.2±0.04	0.149	0.141

Values shown are mean ±s.e.

[a]Adjusted for age, energy intake, alcohol intake, smoking, physical activity, per capita annual income, education level, residence(urban/rural) and geographical regions.

[b]Statistical significance was determined by analysis of covariance.

Compared with Number of MetS components 0 group,*P＜0.05, **P＜0.01.

Supplemental Table 3. Energy and multivariate adjusted[a,b] mean nutrient intake according to the number of metabolic syndrome (MetS)components in women (N=3234)

	Number of MetS components					
	**0**	**1**	**2**	**3−5**	P^b	Trend P
n，%	866（30.9%）	832（29.7%）	618（22.1%）	484（17.3%）		
Energy	2349.9±20.8	2317.5±21.3	2296.7±24.6	2269.6±27.8	0.109	0.016
Nutrients[a]						
VitA(μg RE)	476.2±31.68	452.7±31.12	463.2±32.27	444.9±32.5	0.665	0.333
$VitB_1$(mg)	0.8±0.02	0.9±0.02	0.9±0.02	0.9±0.02	0.412	0.165
$VitB_2$(mg)	0.7±0.02	0.7±0.02	0.7±0.02	0.7±0.02	0.826	0.601
$VitB_3$(mg)	13.4±0.27	13.4±0.26	13.4±0.27	13.5±0.27	0.999	0.868
K(mg)	1605.5±25.61	1603.7±25.20	1608.4±26.14	1582.2±26.31	0.612	0.353
Zn(mg)	10.1±0.15	10.3±0.15	10.2±0.15	10.3±0.15	0.455	0.421
Se(μg)	38.1±0.78	39.2±0.77	38.9±0.80	39.0±0.80	0.312	0.270
P(mg)	900.6±12.84	916.0±12.62	910±13.08	915.1±13.18	0.429	0.284
Mn(mg)	5.5±0.20	5.7±0.19	5.8±0.20	5.6±0.20	0.384	0.575
Cu(mg)	1.8±0.04	1.9±0.04	1.9±0.04	1.9±0.04	0.325	0.132

Values shown are mean ±s.e.

[a]Adjusted for age, energy intake, alcohol intake, smoking, physical activity, per capita annual income, education level, residence(urban/rural) and geographical regions.

[b]Statistical significance was determined by analysis of covariance.

Compared with Number of MetS components 0 group,*$P<0.05$, **$P<0.01$.
