# Supplementary material for: Relationship between dietary factors and the number of altered metabolic syndrome components in Chinese adults: a cross-sectional study using data from the China Health and Nutrition Survey
Source: BMJ Open. 2017 May 29;7(5):e014911. doi: 10.1136/bmjopen-2016-014911 (PMC5729973; doi:10.1136/bmjopen-2016-014911)
Supplement: Author's manuscript [file bmjopen-2016-014911.draft_revisions.pdf]

BMJ Open

Relationship between dietary factors and the number of altered metabolic syndrome components in Chinese adults

Journal:	*BMJ Open*
Manuscript ID	bmjopen-2016-014911
Article Type:	Research
Date Submitted by the Author:	01-Nov-2016

Complete List of Authors:	Cheng, Maowei; National Institute for Nutrition and Health, Chinese Center for Disease Control and Prevention; Center for Disease Control and Prevention of Hubei Province<br>Wang, Huijun ; National Institute for Nutrition and Health, Chinese Center for Disease Control and Prevention<br>Wang, Zhihong ; National Institute for Nutrition and Health, Chinese Center for Disease Control and Prevention<br>Du, Wenwen ; National Institute for Nutrition and Health, Chinese Center for Disease Control and Prevention<br>Ouyang, Yifei ; National Institute for Nutrition and Health, Chinese Center for Disease Control and Prevention<br>Zhang, Bing; National Institute for Nutrition and Health, Chinese Center for Disease Control and Prevention

**Primary Subject Heading**:	Public health
Secondary Subject Heading:	Nutrition and metabolism
Keywords:	Adults, Chinese, Components, Dietary factors, Metabolic syndrome

SCHOLARONE™
Manuscripts

Relationship between dietary factors and the number of altered metabolic syndrome components in Chinese adults

Maowei Cheng[1,2], Huijun Wang[1], Zhihong Wang[1], Wenwen Du[1], Yifei Ouyang[1],and Bing Zhang[1,*]

[1]National Institute for Nutrition and Health, Chinese Center for Disease Control and Prevention, Beijing, China

[2] Center for Disease Control and Prevention of Hubei Province, Wuhan, China

[*] **Corresponding author:** Bing Zhang

E-mail: zzhangb327@aliyun.com

Postal address: No.27 Nan Wei Rd., Xi Cheng District, Beijing, zip code:100050

Telephone: 010-83151561(office), 18601109679(Mobile phone)

Fax numbers: 010-67711813

Keywords: Adults; Chinese; Components; Dietary factors; Metabolic syndrome

Word count: 4738 words

For peer review only - http://bmjopen.bmj.com/site/about/guidelines.xhtml

For peer review only

Abstract

Objectives: This study aims to study the correlation between dietary factors and the number of altered MetS components in Chinese adults systematically.

Setting: Data were derived from urban and rural communities in nine provinces in China.

Participants: There were 6034 eligible subjects (2800 men and 3234 women) in this study.

Outcomes: The primary outcome of this study was diet assessments and the number of altered MetS components. Dietary intake was measured using a 3 day period with 24-hour recalls, and a household food inventory, and average daily intakes of nutrients were estimated according to the Chinese Food Composition Table. Blood samples were analyzed in a national central lab and the number of clustering MetS components was calculated by adding the presence of each MetS component.

Results: After adjusting for covariates, the high risk factors correlating with increased numbers of altered MetS components in men were higher intake of protein (70.4–73.4g; trend test *P*=0.0004), cholesterol(238.7–266.6mg;trend test *P*=0.004), meat(90.6–105.7g; trend test *P*=0.016),fish/seafood(30.4–42.3g; trend test *P*=0.001), and lower intake of coarse cereals(16.5–12.7g; trend test *P* = 0.051), tubers(37.3–32.7g; trend test *P* = 0.030), and dietary fiber(11.7–11.5 g; ANCOVA *P*= 0.058). The high risk factors correlating with increased number of altered MetS components in women were higher intake of wheat(101.9–112.6g; trend test *P*=0.066) and sodium(3862.3–4005.7mg; trend test *P*=0.032), and lower intake of ß-carotene(1578.6–1382.7µg; trend test *P*=0.007), milk, and dairy products(17.8–11.5g; trend test *P*=0.002).

Conclusions: The intake of some foods and nutritional components correlates with increased numbers of altered MetS components in Chinese adults. A choice of healthy sources of fat, proteins, and carbohydrates plus a moderate intake of meat and alcohol in men, together with avoidance of refined carbohydrates and sodium in women, would help prevent MetS in China.

For peer review only - http://bmjopen.bmj.com/site/about/guidelines.xhtml

Strengths and limitations of this study

1. This is the first observational study to examine relations between dietary factors and the number of altered MetS components among Chinese adults in a systematic way.
2. This study include the large sample size, fairly representative of the Chinese population and that three 24-hour recalls, plus a household inventory were available for each participant. Furthermore, only healthy adults, not using chronic medication were included.
3. The major limitation of this study is the inability to come to any causal conclusion due to the cross-sectional design.

INTRODUCTION

Metabolic syndrome(MetS) is a cluster of risk factors (dysglycemia, elevated blood pressure, dyslipidaemia, and central adiposity) that has been shown to be predictive of cardiovascular disease and diabetes.[1]

The overall age-standardized estimates of MetS prevalence in Chinese adults were 18.2%, according to the International Diabetes Federation, and 21.3%, according to the modified Adult Treatment Panel III criteria of the National Cholesterol Education Program(NCEP-ATP III). These estimates of MetS prevalence increase gradually with age.[2] Therefore, MetS is considered as a major challenge that affects the quality of life of millions of people. It is urgent to identify effective strategies to better control MetS.

MetS is estimated using five components: waist circumference(WC), fasting glucose, high density lipoprotein cholesterol (HDL-C), triglycerides, and hypertension[1].MetS in individual patients, may involve the alteration of various combinations of these components. Accumulating evidence has suggested that an increased number of altered MetS components is strongly associated with a higher risk of developing diseases.[3–6] Serum magnesium level decreased as the number of altered MetS components increased in elderly Greek patients[5]. The number of altered MetS components in middle-aged and elderly Japanese patients was related to lower intake of vitamin B_6,[6] and lower dietary fiber in men, and to lower intake of calcium, milk, dairy products, and higher cereal intake in women, after adjusting for age, energy intake, alcohol intake, smoking status, physical activity.

For peer review only - http://bmjopen.bmj.com/site/about/guidelines.xhtml

An increasing number of studies in China have focused on the influence of dietary patterns on the prevalence of MetS and its altered components. Using data from the 2002 China National Nutrition and Health Survey(CNNHS),three types of dietary patterns have been identified in the Chinese population. One dietary pattern is called “Yellow Earth” or “traditional northern” pattern; it is characterized by consumption of refined cereal products, salted vegetables and tubers. Another dietary pattern is called “Western/new affluence”; it is characterized by consumption of beef, lamb, milk, cheese, and yogurt. The third dietary pattern is called “Green Water” or “traditional southern” pattern; it is characterized by consumption of rice, vegetables, and moderate amounts of animal foods.[7–9] The “Yellow Earth” pattern and the “Western/new affluence” pattern were associated with an increased likelihood of MetS, or with increased alterations of its components,[8 9] in comparison to the “Green Water” dietary pattern. Our previous studies have identified a modern high-wheat pattern that is characterized by consumption of wheat products, nuts, fruits, eggs, milk and instant noodles or frozen dumplings. This modern pattern could be considered as a combination of the “Yellow Earth” and “Western/new affluence” patterns, and has been positively associated with diabetes.[10 11] Additionally, in our previous studies an “alcohol” dietary pattern was identified among men. This dietary pattern is characterized by consumption of meat, alcohol, and nuts, and has been positively associated with overweight/obesity and central obesity.[12]

However, analysis of the relationship between MetS and single foods or nutrients in previous studies provided results that were not consistent with the analysis of the relationship between MetS and dietary patterns. Diet with a higher content of animal foods, and higher content of animal proteins protected people from glucose metabolic disorders and hypertension. It should be noted that the idea that the Chinese traditional dietary structure is dominated by cereals should not be over emphasized.[13 14] More importantly, because diet is a complex variable, it is necessary to adopt multiple approaches to examine the relationship between diet and the risk of diseases. If there were any effects caused by a particular nutrient, the dietary pattern approach would not be optimal to examine the relationship between diet and risk of diseases because the effect of that nutrient would be diluted by other factors.[15] In addition, it would be difficult to obtain an identical relationship between dietary patterns(or dietary index) and the relevant disease outcomes in different studies[16].

There are significant differences in the dietary characteristics of men and women.[17 18] To our

For peer review only

knowledge, the nutrients that could have a positive effect on MetS or on its components have not been determined in men and women, and no studies have explored the association between dietary factors and the number of altered MetS components in the Chinese population. The aim of our study was to examine the relationship between dietary factors and the number of altered MetS components in a systematic way, using a large sample of Chinese adults (N = 6034).

METHODS

Study population

The China Health and Nutrition Survey(CHNS) is a longitudinal survey of health and nutrition conducted from 1989 to 2009, and the data were collected in 1989, 1991, 1993, 1997, 2000, 2004, 2006 and 2009, respectively. The survey was designed to examine the association among economic and social changes and a range of health behaviors across space and time.[10] A multistage, random cluster sampling method was used to select the samples from nine provinces(Liaoning, Heilongjiang, Jiangsu, Shandong, Henan, Hubei, Hunan, Guangxi and Guizhou) in China. Questionnaire information were used to collect the data as follows: social and economic factors, health factors, nutrition factors and population factors of sociology. However, blood samples were collected only at the first time in 2009. More details about the CHNS data have been described elsewhere.[19]

This analysis focused on 7755 respondents(3589 men and 4166 women)with ages of ≥18 years(in 2009).Individuals with incomplete dietary data (n=296), over 75 years of age (n=419), suffering from chronic diseases (hypertension, diabetes, stroke, myocardial infarction) with a change in diet or physical and/or pharmacological treatment (n=906), and women in pregnancy or lactation status (n=100) were excluded. Finally, a total of 6034 adults(2800 men and 3234 women) were included in this analysis.

Diet assessments

Data were collected over three consecutive days by 24-hour dietary recall and a household inventory. Average daily intakes of nutrients, including energy, fat, protein, carbohydrates, dietary fiber, beta carotene, vitamin C, vitamin E, calcium, sodium, iron, magnesium, ratio of energy from protein and fat, and ratio of protein from various foods were estimated according to the Chinese

For peer review only - http://bmjopen.bmj.com/site/about/guidelines.xhtml

Food Composition Table(2nd edition).[20] Twelve kinds of nutrients, 13 kinds of foods, ratio of energy from protein and fat, and ratio of protein from various foods were selected as diet indicators according to previous studies.[6 21 22]

Other measurements

Weight, height and waist circumference were measured by trained surveyors using standard measurement techniques: Height was measured without shoes to the nearest 0.2cm using a portable stadiometer. Weight was measured without shoes and in light clothing to the nearest 0.1kg on a calibrated beam scale.[23] Between the lowest rib and the iliac crest in a horizontal plane, waist circumference was measured at a point midway using non-elastictape.[24]According to a standard protocol, blood pressure was measured by trained surveyors using a mercury sphygmomanometer.[25]

Blood samples were collected by vein puncture after an overnight fast. All samples were analyzed in a national central lab in Beijing (medical laboratory accreditation certificate ISO 15189:2007) under strict quality control. Fasting plasma glucose was measured with a glucose oxidase phenol 4-aminoantipyrine peroxidase kit(Randox, Crumlin, UK) in a Hitachi 7600 automated analyzer(Hitachi Inc., Tokyo, Japan).[11 26] High-density lipoprotein cholesterol (HDL-C) and triglycerides (TG) were both measured by their corresponding reagents(Kyowa MedexCo., Ltd., Tokyo, Japan)using glycerol-phosphate oxidase method, and the polyethylene glycol modified enzyme method, respectively. All lipid measurements were carried out on the Hitachi 7600 automated analyzer (Hitachi Inc., Tokyo, Japan).[27] History of chronic patients (past/present), smoking drinking (y/n) and the occupational physical activity were collected using questionnaire.

Definition of MetS and its components

The definition of MetS was based on the most recent Joint Interim Statement (JIS) criteria.[28] As mentioned earlier, subjects under treatment for chronic diseases (hypertension, diabetes, stroke, myocardial infarction) were excluded in present study. Thus, MetS was defined as the presence of three or more of the following five components: (1)abdominal obesity, WC men⩾90cm and women⩾80cm specifically for Asia adults;[6] (2)elevated blood pressure, SBP/DBP⩾130/85mmHg; (3) hypertriglyceridemia, triglyceride ⩾ $1.70 mmoll^{-1}$; (4) low high-density lipoprotein

For peer review only - http://bmjopen.bmj.com/site/about/guidelines.xhtml

cholesterol(HDL-C), HDL-C < 1.0mmoll^{-1} in men and < 1.3 mmoll^{-1} in women; and (5) elevated blood glucose levels, fasting blood glucose ≥ 5.6 mmoll^{-1}.The number of clustering MetS components was calculated by adding the number of MetS components.[6]

Definition of covariates

According to the definition of WHO, the adults who smoked at least one cigarettes a day currently was defined as current smoker.[12] For drinker, individuals was asked questions: "Have you consumed alcohol(beer, wine or other alcoholics) during the past year (yes, no)?".[2] Residences was divided into urban and rural and geographical region was divided into the North(Liaoning, Heilongjiang, Henan and Shandong) and the South (Hunan, Hubei, Jiangsu, Guangxi and Guizhou).[12] BMI was calculated as weight in kilograms divided by the square of height in meters.

Current economic status was assessed by mean per capita annual income(unit: RMB Yuan) in the year before the 2009 CHNS. The subjects were classified as low, moderate, and high income level by trisected percentiles of the mean per capita annual income. Participants were interviewed using a semi-quantitative assessment to determine their occupational, domestic, travel, and leisure physical activity levels. The intensity (metabolic equivalent, METs, unit kcal/kg h) of each activity in the questionnaire was coded according to the compendium of physical activities.[29] The total metabolic equivalent (MET-hours/week) was a combined score calculated by multiplying the frequency, duration, and intensity of physical activity. Total metabolic equivalent scores were categorized into three levels(mild, moderate, and high)by trisected percentiles for further analysis.

Statistical analysis

Statistical Analysis System 9.2 (SAS Institute, Cary, NC, USA)was used for all statistical analyses. Subjects were categorized into four groups according to the number of clustering MetS components (0, 1, 2, 3-5).Values of three to five clustering MetS components were combined because only a few subjects had alterations in four or five MetS components (four altered components: 121 men and 215 women; five altered components: 28 men and 64 women). Associations between categorical variables were tested using Chi-Square Test. Comparisons between continuous variables were performed by analysis of variance, and 95% confidence intervals (CI) were estimated.

For peer review only - http://bmjopen.bmj.com/site/about/guidelines.xhtml

For peer review only

Assessment of model effects and trend tests were performed to analyze the association between each of the dietary indicators and the number of altered MetS components using a generalized linear regression model. Adjustments were made for all potential confounding factors, including age, energy intake, alcohol intake, smoking status, per capita annual income, education levels, physical activity levels, residence(urban/rural) and geographical region(North/South). Mean nutritional intakes were calculated by the number of MetS components (0, 1, 2, 3-5).The estimated marginal means for each of dietary indicators of altered MetS components groups1, 2, and 3-5 were compared with those of MetS components group 0 by multiple comparison test adjusted by Bonferroni method.

All reported *P*-values were estimated using two-sided analysis. A *P*-value <0.05 was considered statistically significant.

RESULTS

Characteristics of the study population

Table 1 shows the characteristics and distribution of all the subjects included in the study categorized by to sex. Mean age ± s.d. was 47.3 ± 13.6 years for men, and 47.6 ± 13.1 years for women. Body mass index (BMI) was 23.0 ± 3.1 kgm^{-2} for men, and 23.0 ± 3.2 kgm^{-2} and for women. The number of altered MetS components in both men and women gradually increased as age and BMI increased .

We estimated that 58.0% of men currently smoked, and 63.0% consumed alcohol; 3.2% of women smoked, and 9.2% consumed alcohol.

Overall, there was a positive association between the number of altered MetS components and the proportion of moderate and high per capita annual income, but only in men. In women, the number of altered MetS components was associated with a lower proportion of individuals in junior middle school and subsequent educational levels. In addition, there was a negative relationship between the number of altered MetS components in men and women and moderate and high physical activity levels.

Prevalence of MetS and metabolic abnormalities

As shown in Table 2, the overall prevalence of MetS was significantly higher in women (22.3%, 95% CI 20.9, 23.7) than in men (17.3%, 95% CI 15.9, 18.7). Significant differences between sexes were noted in the analysis of individual components. Elevated blood pressure,

For peer review only - http://bmjopen.bmj.com/site/about/guidelines.xhtml

hypertriglyceridemia, and hyperglycemia were more frequent in men than in women. Abdominal obesity and low HDL cholesterol were more frequent in women than in men. In men hypertension was the most prevalent component of MetS (38.6%), followed by hypertriglyceridemia(31.5%). In women, the most prevalent MetS component was abdominal obesity(49.4%), followed by low HDL-C(31.0%).

Food and nutrient intake in correlation with different MetS components in men

Table 3 shows multivariate adjusted mean food and nutrients intake, according to the number of altered MetS components in men. Higher daily intake of meat(90.6–105.7g) and fish/seafood(30.4–42.3g)was positively correlated with an increased number of altered MetS components(trend test *P*=0.016 for meat; trend test *P*=0.001 for fish/seafood).Although analysis of covariance or trend tests did not reach statistical significance, the number of altered MetS components were positively correlated with daily intakes of visceral meat(2.9–4.4g; trend test *P* = 0.050), beans (17.0–19.5g; trend test *P* = 0.113), and nuts (3.2–4.1g; trend test *P*=0.207).

Among the dietary indicators, there was a negative correlation between the number of altered MetS components and the daily intake of cereals (453.9– 440.8g; ANCOVA *P* = 0.083, trend test *P* = 0.058), coarse cereals (16.5–12.7g; trend test *P* = 0.051), and tubers (37.3–32.7g; ANCOVA *P* = 0.048, trend test *P* = 0.030).

An increased number of altered MetS components were positively correlated with a higher daily intake of protein (70.4–73.4g; trend test *P*=0.0004) and cholesterol (238.7–266.6mg trend test *P*=0.004). It is worth noting that there was a positive correlation between an increased number of altered MetS components and an increased energy daily intake of protein (12.3%–12.8%; ANCOVA *P*=0.001, trend test *P*=0.0001),and with increased daily intake of protein of animal origin (28.6%–30.4% ; ANCOVA *P*=0.021, trend test *P*=0.020).

Among the dietary indicators, there was a negative correlation between the number of altered MetS components and the intake of dietary fiber (11.7–11.5 g; ANCOVA *P*= 0.058). Cholesterol levels in the groups of MetS components1, 2 and 3-5 were significantly higher than in the group 0 (multiple comparison test *P*=0.006, *P*=0.003,*P*=0.012).

Food and nutrient intake in correlation with different MetS components in women

Table 4 shows the multivariate adjusted mean food and nutrient intake,according to the number of MetS components in women. There was a positive correlation between the number of

For peer review only

altered MetS and the daily intake of cereals(345.3–351.6g),especially for wheat (101.9–112.6g; trend test *P*=0.066).In addition, there was a negative correlation between the number of altered MetS components and the daily intake of milk and dairy products (17.8–11.5g; ANCOVA *P*=0.020, trend test *P*=0.002).The daily intake of milk and dairy products for the group 3-5 of altered MetS components was significantly lower than the intake of the group 0 (multiple comparison test *P*=0.009).

Higher daily intake of sodium was positively correlated with an increased number of altered MetS components (3862.3–4005.7mg; trend test *P*=0.032).However, as the daily intake of beta carotene decreased(1578.6–1382.7μg; ANCOVA *P*= 0.001, trend test *P*=0.007) the number of MetS components increased. The daily intake of beta carotene in the altered MetS components groups 1 and 3-5 MetS was significantly lower than in group 0 (multiple comparison test *P*=0.001 and *P*=0.003).

For peer review only

DISCUSSION

Using data from a partially national representative sample of Chinese adults, we found that higher daily intake of meat, fish/seafood, protein, cholesterol, and lower daily intake of coarse cereals, tubers, and dietary fiber was correlated with the number of altered MetS components in men. We also found that higher daily intake of wheat and sodium, and lower intake of beta carotene, milk, and dairy products was correlated with the number of altered MetS components in women. Rather than using a dietary pattern method of analysis, it might be more accurate to evaluate the effect of the single foods or nutrients on the risk of MetS, using a dose-effect relationship analysis systematically.[3-6]

Our study found that the increased intake of meat and fish/seafood was positively associated with the number of altered MetS components in the Chinese male population. This correlation is closely related to the rapid evolution of the diet in China, due to the remarkable economic developments and changes in lifestyle since 1980s. During this social revolution, the popular diet pattern shifted from the traditional Chinese food, composed mainly of cereals, vegetables and few animal foods, to the consumption of a western diet (e.g. high energy, high fat, high protein, and low dietary fiber).[30] High energy density in carbohydrate-rich food is evidently linked with high levels of obesity and nutrition-related non-communicable diseases.[31]

The high intake of fat from meat, particularly saturated fat, has been associated with higher plasma lipoprotein levels and higher blood pressure levels.[32] A higher daily intake of cholesterol from visceral meat increases the risk of cardiovascular disease.[33] In a cohort study of Japanese ancestry, saturated fatty acids played a key role in the relationship between red meat consumption and the risk of developing the MetS[22]. According to the Dietary Reference Intake(DRIs) for Chinese adults, general recommendations propose a total fat intake of 20%–30% of the daily caloric consumption with an emphasis on unsaturated fat; saturated fat should represent <10% of total ingested calories. In opposition to these general recommendations, our study showed that intake of fat (% of energy) was slightly higher than the recommended value in both genders (e.g.31.9% in men, and 33.3% in women with three or more altered MetS components). However, a positive correlation between cholesterol intake and the number of MetS components was only found in men in our study. This observation suggests that a high fat diet among Chinese adults might increase the prevalence of MetS or the prevalence of alterations of its components in men, to a greater extent than in women.

Intake of dietary protein also influences the MetS components, through the percent of protein from various foods, or through quality supplementation of a specific amino acids.[34] A review of epidemiologic evidence indicated that intake of dietary animal proteins appears to increase the risk of MetS.[35] Gadgil MD et al. found that a diet high in animal protein was associated with higher BMI, higher WC, higher total cholesterol, and higher low density lipoprotein cholesterol in South Asians living in the United States.[36] According to the DRIs for Chinese adults, recommendations for daily protein intake are 65g for men and 55g for women.[37] Our study showed that protein intake was also somewhat higher than the recommended amount both in men and women(e.g.73.4–61.4g per day in men and women with three or more altered MetS components).Interestingly, a positive correlation trend between the intake of dietary protein, the percent of protein ingested from animal food(protein %) and the number of MetS or alterations of its components was only observed in men, further indicating that a high animal protein diet in men was responsible for this correlation.[34]

The higher iron content of red meat might be related to the higher prevalence of MetS. Park et al. reported that elevated serum ferritin levels were independently associated with future development of MetS, during a 5 year follow-up period.[38] Additionally, previous studies found that

For peer review only - http://bmjopen.bmj.com/site/about/guidelines.xhtml

serum ferritin levels were higher in men than in women in Chinese adults.[39] We found a positive association between higher intake of meat and an increased number of altered MetS components only in men. Moreover, our study found that mean iron intake in men was positively correlated to the number of altered MetS components, after adjusting for potential confounding factors.

Finally, drinking alcohol may be another factor that increases the risk of MetS. Our previous study found that an "alcohol" dietary pattern was present in Chinese men.[12] In this study the overall prevalence of alcohol consumption is 64.9% in men and 9.2% in women, with three or more altered MetS components. Moderate alcohol intake has consistently been shown to be associated with a lower risk of fatal and non-fatal cardiovascular disease. Wine is an important component of the Mediterranean diet,[40] but heavy drinking was positively associated with MetS and alterations of its components.[41] More recently, a positive association was observed between a diet rich in meat and alcohol and MetS or alterations of its individual components(hypertriglyceridemia, elevated blood pressure) in Korean male adults.[32] Thus, it is not hard to accept the strong relationship between meat intake and the increased number of altered MetS components observed only in Chinese men in the present study.

High cereal fiber content and low glycemic index are inherent attributes of most whole-grain foods.[3] Coarse cereals and tubers can provide rich dietary fiber, less fat, and less protein than animal food.[12] According to the recommendation from Chinese Nutrition Society, a daily fiber intake for Chinese adults should be 25 g to 30 g/day.[37] Our data showed that mean fiber intake was very lower in both genders (e.g. 11.5g and 10.3g per day in men and women with three or more altered MetS components). Similarly to a study of middle-aged and elderly Japanese people,[6] we found a marginally significant correlation between fiber intake in men and the number of altered MetS components. Dietary fiber has the ability to control body weight (through its effect on satiety), to modulate glucose homeostasis and insulin sensitivity, and to positively affect cardiovascular disease risk factors.[42 43] Other data indicated that the protective role of fiber from cereals was higher than that from other sources fiber for the development of MetS and type 2 diabetes.[3] Our study suggested that fiber, which mainly comes from coarse cereals and tubers, possibly played an important role in reducing the risk of MetS in men. Magnesium is another component of whole grains that may improve insulin sensitivity.[5] However, dietary magnesium intake in both genders was not correlated with the number of altered MetS components in the

For peer review only - http://bmjopen.bmj.com/site/about/guidelines.xhtml

present study.

Interestingly, our study showed a marginally significant positive correlation between wheat intake and the number of MetS components only in women, after adjusting for potential confounding factors. In China, wheat and rice belong to dietary patterns with different characteristics; wheat tends to be a major component of a modern high-wheat dietary pattern, as mentioned earlier.[7–10] One explanation for this correlation is that the refining wheat process before cooking results in the removal of healthy wheat constituents(e.g. fiber, vitamins, minerals, phytonutrients, and essential fatty acids).[40] Thus, higher carbohydrate consumption (e.g. higher intake of highly refined starch and sugar) could have adverse effects on the profile of metabolic risk[44]. In addition, high glycemic index foods (such as noodles, bread and steamed bread) can induce inflammation, increase oxidant activities, and cause rapid fluctuations of blood glucose and insulin levels.[45] A "refined bread" dietary pattern was associated with hyperinsulinemia in women. [46] HDL cholesterol levels were negatively associated to carbohydrate and glycemic indexes[35 47 48].Similar to previous studies, we observed lower HDL cholesterol levels in women, which may be partly due to the effects of a higher intake of refined carbohydrates, more so than in men. Another explanation for this association is the frying cooking style for wheat or its products. Females tend to have a high intake of refined carbohydrates, most of which is prepared with wheat (e.g. snacks typically fried in vegetable oil) or with high levels of salt.[9 10] A stronger positive correlation was observed between sodium intake and the number of altered MetS components in women, which may reflect female dietary habits. High sodium diet not only elevated blood pressure, but also reduced insulin sensitivity.[49]

In keeping with previous studies, [6 50–52] our data showed that the intake of beta carotene, as well as of milk and dairy products was negatively associated with the number of altered MetS components in women. Carotenoids, mainly found in fruit and fresh vegetables, can capture free radicals, quenching singlet oxygen, and improve the protective capability of antioxidants.[53] Milk, rich in calcium and vitamin D, could accelerate the elimination of abdominal fat.[51] More recently, the Chinese Nutrition Society recommended a daily intake of fruits of 200 g to 350 g per capita per day, and a daily intake of milk or its products of 300g per capita per day for the general Chinese population.[54] In contrast to these recommendations, the mean dietary intake of fruits and milk and dairy products was very low for men and women(e.g.48.5 g or 73.5g of fruit per day in men and

For peer review only - http://bmjopen.bmj.com/site/about/guidelines.xhtml

women with three or more altered MetS components, respectively; 9.2 g and 11.5 g milk and dairy products per day in men and women, with three or more MetS components, respectively).

A strong negative dose-effect relationship between the intake of beta carotene and milk and the number of altered MetS components was observed only in women. We could not identify the underlying mechanism for this negative correlation. A possible explanation for this correlation may be the higher intake of fruits observed in women in the present study. Previous studies reviewed the effects of the biochemical components found more commonly in fruits than in vegetables.[34] Another explanation could be that an additional factor differently modulates digestion, absorption, and cell metabolism in men and women.[46] Therefore, our study suggests that increasing the intake of beta carotene, milk and dairy products could have a great potential to prevent MetS or the alteration of its components in the Chinese population, especially among women.

Fruits, vegetables, whole grains, fish, nuts, and dairy products were six kinds of food that may be beneficial for preventing MetS in various dietary patterns, such as the Mediterranean dietary patter, dietary approaches to stop hypertension, and the Nordic diet.[55] It is well known that the Mediterranean dietary patter as important features, represented by the daily intake of whole-grain, fruits, vegetables, and dairy products, as well as the weekly intake of fish, poultry, nuts, and beans. [56] Similar to previous multiethnic reports, our study showed that the intake of fruits and vegetables was negatively correlated to the number of altered MetS components, but not with statistical significance for either men or women. In our study a higher intake of fish/seafood(e.g. fish, shrimp, crab, and shellfish), beans, and nuts was positively correlated with an increased number of altered MetS components in men. This inconsistence may be due to the existence of an "alcohol" dietary pattern, characterized by animal foods(e.g. meat, fish/seafood), alcohol, and nuts in the Chinese male population,[12] the fact that in China fish/seafood is preserved with high levels of salt or fried in vegetable oil or the fact that the intake of fried dough with soya-bean milk is a typical breakfast for Chinese people[11 33].

This study has several limitations. First, because of our cross-sectional design, we could not come to any causal conclusion to explain the relationships between dietary factors and the number of altered MetS components. Second, the mixed intake of food and nutrients may produce more complex interactive effects than the analysis of the intake of foods and nutrients as evaluation indicators.[15] For instance prior findings have shown that the "Green Water" dietary pattern, which

For peer review only - http://bmjopen.bmj.com/site/about/guidelines.xhtml

is characterized by high intake of rice, vegetables, and moderate intake of animal foods, has beneficial effects on MetS or on its components.[7–9] There was not a significant negative correlation between rice and vegetables and the number of altered MetS components in both genders in the present study. Third, there maybe residual confounding factors in this study. Due to lack of relevant information, some important variables(e.g. the type and source of meats, dairy products, fat, dietary fiber, and alcohol, gender differences in the ability for digestion, absorption, and cell metabolism) were excluded from analysis, which may limit the strength of our findings. Fourth, our study is just a partially national representative sample that only included nine provinces of China. Finally, in this study we measured diet using a 3 day period with 24-hour recalls, but it is unclear whether short-term records of dietary recall can fully reflect the long-term intake of a dietary pattern.[57]

CONCLUSIONS

In conclusion, our study showed that some foods and nutritional components are correlated to the number of altered MetS components among the Chinese adult population. This observation suggests that a choice of healthy sources of fat, proteins, and carbohydrates, together with a moderate intake of meat and alcohol in men, and avoidance of high intake of refined carbohydrate and sodium in women, would be critical to prevent MetS in China. More research is needed to examine these associations in prospective studies and to develop more appropriate nutritional recommendations to prevent MetS according to gender differences.

Contributors MWC, BZ conceived the study. MWC, YFOY and WWD completed all statistical analyses and interpretation of data. MWC drafted the manuscript. ZHW and WWD contributed to the discussion. HJW and BZ revised the manuscript.

Funding The work was supported by the National Institutes of Health(NIH: R01-HD30880, DK056350 and R01-HD38700).

Competing interests None.

Data sharing statement The data used for the analysis are available from the CPC at http://www.cpc.unc.edu/projects/china.

For peer review only - http://bmjopen.bmj.com/site/about/guidelines.xhtml

REFERENCES

1. Ahluwalia N, Andreeva VA, Kesse-Guyot E, *et al*. Dietary patterns, inflammation and the metabolic syndrome. *Diabetes Metab* 2013;39:99–110.
2. Xi B, He D, Hu Y， *et al*. Prevalence of metabolic syndrome and its influencing factors among the Chinese adults: The China Health and Nutrition Survey in 2009.*Prev Med* 2013;57: 867–71.
3. McKeown NM, Meigs JB, Liu S， *et al*. Carbohydrate nutrition, insulin resistance, and the prevalence of the metabolic syndrome in the Framingham Offspring Cohort. *Diabetes Care* 2004;27:538–46.
4. Ford ES, Giles WH, Dietz WH.Prevalence of the metabolic syndrome among US adults: findings from the third National Health and Nutrition Examination Survey. *JAMA* 2002;287: 356–59.
5. Evangelopoulos AA,Vallianou NG, panagiotakos DB， *et al*. An inverse relationship between cumulating components of the metabolic syndrome and serum magnesium levels. *Nutr Res* 2008;28: 659–63.
6. O Rei，I Tomoko，K Yuki， *et al*. Relationship between number of metabolic syndrome components and dietary factors in middle-aged and elderly Japanese subjects. *Hypertens Res* 2010;33:548-54.
7. Li Y, He Y, Lai J， *et al*. Dietary patterns are associated with stroke in Chinese adults. *J Nutr* 2011;141:1834–39.
8. Wang D, He Y, Li Y， *et al*. Dietary patterns and hypertension among Chinese adults: a nationally representative cross-sectional study. *BMC Public Health* 2011;11:925.
9. He Y, Li Y, Lai. Dietary patterns as compared with physical activity in relation to metabolic syndrome among Chinese adults. *Nutrition, Metabolism & Cardiovascular Diseases* 2013;23:920-28.
10. Batis C, Sotres-Alvarez D, Gordon-Larsen P，*et al*. Longitudinal analysis of dietary patterns in Chinese adults from 1991 to 2009. *Br J Nutr* 2014;111:1441–51.
11. Batis C,Mendez MA, Gordon-Larsen P， *et al*. Using both principal component analysis and reduced rank regression to study dietary patterns and diabetes in Chinese adults. *Public Health*

For peer review only - http://bmjopen.bmj.com/site/about/guidelines.xhtml

Nutr 2016;19:195-203.

12. Zhang J. Changes in dietary patterns and their associations with general and central obesity among adults in China (1991-2009).PhD Thesis, Chinese Center for Disease Control and Prevention, 2013.
13. Zhao Y. The study of relationship between dietary patterns and metabolic syndrome among adult in Rural original Huis of Ningxia [Master's degree thesis]. Ningxia Medical University; 2009.
14. Xia DY. Nutrition and metabolic disorders：A preliminary study in southern China [PhD thesis]. Peking Union Medical University;2004.
15. Hu FB. Dietary pattern analysis: A new direction in nutritional epidemiology. *CurrOpin Lipidol* 2002;13:3–9.
16. Mishra GD, McNaughton SA,Bramwell GD，*et al*. Longitudinal changes in dietary patterns during adult life.*Br J Nutr* 2006;96:735-44.
17. Ashima K. Kant. Dietary patterns and health outcomes. J*ournal of the American Dietetic Association* 2004;104:615-35.
18. Ma G, Kong L(editor). The report of 2002 China National Nutrition and Health Survey(Part Nine)－behavior and way of life. Beijing: People's Medical Publishing House,2006.
19. Popkin B.M., DuS, Zhai, F, *et al*. Cohort profile: the China health and nutrition surve-monitoring and understanding socioeconomic and health change in China, 1989–2011. *Int. J. Epidemiol* 2010;39:1435–40.
20. Yang Y, Wang G, Pan X (editor). China food composition table. 2nd edition. Beijing: Beijing Medical University Press,2009.
21. Millen BE, Pencina MJ, Kimokoti RW，*et al*. Nutritional risk and the metabolic syndrome in women: opportunities for preventive intervention from the Framingham Nutrition Study. *Am J Clin Nutr* 2006; 84:434–41.
22. Dami ˜;ao R, Castro TG, Cardoso MA，*et al*. Dietary intakes associated with metabolic syndrome in a cohort of Japanese ancestry. *Br J Nutr*2006; 96:532–38.
23. Bell AC, Ge K, Popkin BM. Weight gain and its predictors in Chinese adults. *Int J Obes* 2001; 25:1079–86.
24. WHO. Physical Status: The Use and Interpretation of Anthropometry. Report of a WHO

For peer review only - http://bmjopen.bmj.com/site/about/guidelines.xhtml

Expert Committee. World Health Organization, Geneva, 1995.

25. Chobanian AV, Bakris GL, Black HR，*et al*. The seventh report of the Joint National Committee on prevention, detection, evaluation, and treatment of high blood pressure: the JNC 7 report. *JAMA* 2003;289:2560–72.

26. Batis C,Mendez MA,Sotres-Alvarez D，*et al*. Dietary pattern trajectories during 15 years of follow-up and HbA1c, insulin resistance and diabetes prevalence among Chinese adults. *J Epidemiol Community Health* 2014;68:773–79.

27. Yan S, Li J, Li S，*et al*. The expanding burden of cardiometabolic risk in China: the China Health and Nutrition Survey. *Obes. Rev* 2012;13:810–21.

28. AlbertCi KG, Eckel RH, Grundy SM，*et al*.Harmonizing the metabolic syndrome: a joint interim statement of the international diabetes federation task force on epidemiology and prevention; national heart, lung, and blood institute; American heart association; world heart federation; international atherosclerosis society; and international association for the study of obesity. *Circulation* 2009;20:1640–45.

29. Ainsworth BE, Haskell WL, Whitt MC, *et al*. Compendium of physical activities: an update of activity codes and MET intensities. *Med Sci Sports Exerc* 2000;32:S498-S504.

30. Du S,Lu B, Zhai F，*et al*. A new stage of the nutrition transition in China. *Public Health Nutr* 2002; 5:169-74.

31. Popkin BM. The nutrition transition: An overview of world patterns of change. *Nutr Rev* 2004;62:S140-S143.

32. Woo1 HD, Shin A, Kim J. Dietary patterns of Korean adults and the prevalence of metabolic syndrome: A cross-sectional study. *PLoS One* 2014;9:e111593.

33. Li Y, Shi X, Hou P，*et al*. Diet, the scientific evidence nutrition and main chronic non-communicable diseases prevention. *Chinese Journal of preventive medicine* 2011; 45:459-62.

34. Andersen JC, Fernandez ML. Dietary strategies to reduce metabolic syndrome. *Reviews in Endocrine & Metabolic Disorders* 2013; 14,241-54.

35. Amanda B, Terry C, Christine M. Dietary patterns and metabolic syndrome-a review of epidemiologic evidence. *Asia Pac J Clin Nutr* 2006;15:134-42.

36. Gadgil MD, Anderson CA, Kandula NR. Dietary patterns are associated with metabolic risk

For peer review only - http://bmjopen.bmj.com/site/about/guidelines.xhtml

factors in south Asians living in the United States. *J Nutr* 2015;145:1211-17.

37. Chinese Nutrition Society(editor).Chinese Dietary Reference Intakes(2013 edition).Beijing: Science Press,2014.

38. Park SK, Ryoo JH， *et al*. Association of Serum Ferritin and the Development of Metabolic Syndrome in Middle-Aged Korean Men: A 5-year follow-up study. *Diabetes Care* 2012;35, 2521–26.

39. Li J, Wang R, Luo D. Association between Serum Ferritin Levels and Risk of the Metabolic Syndrome in Chinese Adults: A Population Study. *PLoS One* 2013;8:e74168.

40. Dario G, Antonio C, Katherine E.The effects of diet on inflammation: Emphasis on the metabolic syndrome. *J Am Coll Cardiol* 2006;48:677–85.

41. Baik I, Shin C. Prospective study of alcohol consumption and metabolicsyndrome. *Am J Clin Nutr* 2008;87: 1455–63.

42. American Association of Cereal Chemists The definitionof dietary fiber. 2001;46:112–129. http://www.aaccnet.org/CerealFoodsWorld/pdfs/dietfiber.pdf.

43. Veldhuis L, Koppes LLJ, Driessen MT.Effects of dietary fibre intake during adolescence on the components of the metabolic syndrome at the age of 36 years: the Amsterdam Growth and Health Longitudinal Study.*J Hum Nutr Diet* 2010;23:601–08.

44. Noakes M, Keogh JB, Foster PR，*et al*. Effect of anenergy-restricted, high-protein, low-fat diet relative to a conventional high-carbohydrate, low-fat diet on weightloss, body composition, nutritional status, and markers of cardiovascular health in obese women. *Am J Clin Nutr* 2005;81:1298–306.

45. Avignon A, Hokayem M, Bisbal C， *et al*. Dietary antioxidants: do they have a role to play in the ongoing fight against abnormal glucose metabolism? *Nutrition* 2012;28:715–21.

46. Wirfält E,Hedblad B,Gullberg B,*et al*. Food Patterns and Components of the Metabolic Syndrome in Men and Women: A Cross-sectional Study within the Malmö Diet and Cancer Cohort. *Am J Epidemiol* 2001; 154:1150–59

47. Merchant AT, Anand SS, Kelemen LE， *et al*. Carbohydrate intake and HDL in a multiethnic population. *Am J Clin Nutr* 2007; 85:225–30.

48. Frost G, Leeds A, Dore C, *et al*. Glycaemic index as a determinant of serum HDL-cholesterol concentration. *Lancet* 1999;353: 1045–48.

For peer review only - http://bmjopen.bmj.com/site/about/guidelines.xhtml

49. Lastra G, Dhuper S, Johnson MS，*et al*. Salt，aldosterone，and insulin resistance: impact on the cardiovascular system. *Nat Rev Cardiol* 2010;7:577–84.

50. Czernichow S,Vergnaud AC, Galan P， *et al*. Effects of long-term antioxidant supplementation and association of serum antioxidant concentrations with risk of metabolic syndrome in adults. *Am J Clin Nutr* 2009;90:329–35.

51. Zemel M, Teegarden D, Van Loan M， *et al*. Dairy-rich diets augment fat loss on an energy-restricted diet: a multicenter trial. *Nutrients* 2009; 1:83–100.

52. Kim J. Dairy food consumption is inversely associated with the risk of the metabolic syndrome in Korean adults. *J Hum Nutr Diet* 2013;26,Suppl 1:S171–S179.

53. Sun C, Ling W, Huang G， *et al*(editor).Nutrition and food hygiene.7th edition. Beijing: People's Medical Publishing House, 2013.

54. Chinese Nutrition Society(editor).Chinese Dietary Guide. Beijing: People's Medical Publishing House, 2016.

55. Calton EK, James AP, Pannu PK，*et al*. Certain dietary patterns are beneficial for the metabolic syndrome: reviewing the evidence. *Nutr Res* 2014; 34;559–68.

56. Esposito K, Kastorini CM, Panagiotakos DB， *et al*. Mediterranean diet and metabolic syndrome: An updated systematic review. *Reviews in Endocrine & Metabolic Disorders* 2013; 14:255–63.

57. Hu FB. Dietary Assessment Methods: Obesity Epidemiology. New York: Oxford University Press, 2008.

For peer review only

Table 1 Subject characteristics according to the number of metabolic Syndrome(MetS) components

Variable	ALL	Number of MetS components				P^a	Trend P
		0	1	2	3-5		
Men							
n，%	2800（100.0%）	866（30.9%）	832（29.7%）	618（22.1%）	484（17.3%）		
Age（year）	47.3±13.6	44.0±14.1	48.0±13.7	48.7±12.8	50.2±12.0	<0.001	<0.001
Body mass index (Kgm^{-2})	23.0±3.1	21.2±2.4	22.5±2.8	24.0±2.8	25.7±2.9	<0.001	<0.001
Energy intake（Kcal per day）	2308.4±610.4	2349.9±614.1	2317.5±607.0	2296.7±601.3	2269.6±27.8	0.109	0.016
Urban residents(n,%)	740(26.4)	181(20.9)	204(24.5)	183(29.6)	172(35.5)	<0.001	<0.001
Northern residents(n,%)	1176(42.0)	300(34.6)	342(41.1)	292(47.2)	242(50.0)	<0.001	<0.001
Moderate and high physical activity(n,%)	1674（59.7%）	556（64.2%）	516（62.0%）	349（56.4%）	253（52.3%）	<0.001	<0.001
Moderate and high per capita annual income(n,%)	1920（68.6%）	612（70.8%）	547（65.9%）	420（68.1%）	341（70.5%）	0.005	0.001
Education level(n,%)							
Primary school and under	913（32.9%）	295（34.3%）	293（35.5%）	193（31.3%）	132（27.7%）	0.062	0.438
Junior middle school	948（34.1%）	289（33.6%）	292（35.4%）	213（34.5%）	154（32.4%）		
High school and above	916（33.0%）	275（32.0%）	240（29.1%）	211（34.2%）	190（39.9%）		
Drinker(n,%)	1764(63.0%)	525(60.6%)	523(62.9%)	402(65.0%)	314(64.9%)	0.267	0.061
Current smoker（n,%）	1623(58.0%)	516(59.6%)	496(59.6%)	341(55.2%)	270(55.8%)	0.189	0.062
Women							
n，%	3234（100.0%）	837（25.9%）	906（28.0%）	770（23.8%）	721（22.3%）		
Age（year）	47.6±13.1	41.2±12.6	46.4±13.1	50.2±12.0	53.7±11.1	<0.001	<0.001
Body mass index (Kgm^{-2})	23.0±3.2	20.8±2.1	22.3±2.9	24.0±2.9	25.3±3.0	<0.001	<0.001
Energy intake（Kcal per day）	1963.4±535.8	1972.5±522.0	1966±532.7	1974±552.5	1938.3±537.4	0.549	0.275
Urban residents（n,%）	948(29.3)	233(27.8)	267(29.5)	208(27.0)	240(33.3)	0.039	0.068
Northern residents（n,%）	1369(42.3)	279(33.3)	388(42.8)	349(45.3)	353(49.0)	<0.001	<0.001
Moderate and high physical activity（n,%）	1921(59.4%)	553（66.1%）	552（60.9%）	453（58.9%）	363（50.3%）	<0.001	<0.001
Moderate and high per capita annual income(n,%)	2119（66.4%）	574（69.0%）	581（65.1%）	487（64.0%）	477（67.4%）	0.032	0.616
Education level(n,%)							
Primary school and under	1508（46.7%）	280（33.5%）	413（45.6%）	406（52.8%）	409（56.8%）	<0.001	<0.001
Junior middle school	1003（31.0%）	297（35.5%）	306（33.8%）	213（27.7%）	187（26.0%）		
High school and above	720（22.3%）	259（31.0%）	187（20.6%）	150（19.5%）	124（17.2%）		
Drinker(n,%)	299(9.2%)	90(10.8%)	79(8.7%)	64(8.3%)	66(9.2%)	0.338	0.243
Current smoker（n,%）	105(3.2%)	18(2.2%)	28(3.1%)	26(3.4%)	33(4.6%)	0.061	0.008

Values shown are mean ± s.d.

[a]Statistical significance was determined by analysis of variance or Chi-Square test.

For peer review only - http://bmjopen.bmj.com/site/about/guidelines.xhtml

Table 2 Prevalence of metabolic abnormalities according to the number of metabolic Syndrome(MetS) components

Men	ALL		Number of MetS components							
			0		1		2		3-5	
	n, %	95% CI	n, %	95% CI	n, %	95% CI	n, %	95% CI	n, %	95% CI
Subtotal	2800（100. 0）	15. 9, 18. 7	866（30. 9）	27. 8, 34. 0	832（29. 7）	28. 0, 31. 4	618（22. 1）	20. 6, 23. 6	484（17. 3）	15. 9, 18. 7
Metabolic abnormalities（n,%）										
Waist circumference≥90（cm）	710(25. 4)	23. 8, 27. 0	0(-)	—	111(13. 3)	11. 0, 15. 6	254(41. 1)	37. 2, 45. 0	345(71. 3)	67. 3, 75. 3
Triglyceride≥1.7(mmoll^{-1})	883(31. 5)	29. 8, 33. 2	0(-)	—	187(22. 5)	19. 7, 25. 3	296(47. 9)	44. 0, 51. 8	400(82. 6)	79. 2, 86. 0
HDL-cholesterol＜1.0(mmoll^{-1})	411(14. 7)	13. 4, 16. 0	0(-)	—	56(6. 7)	5. 0, 8. 4	119(19. 3)	16. 2, 22. 4	236(48. 8)	44. 3, 53. 3
Blood pressure≥130/85(mmHg)	1081(38. 6)	36. 8, 40. 4	0(-)	—	368(44. 2)	40. 8, 47. 6	354(57. 3)	53. 4, 61. 2	359(74. 2)	70. 3, 78. 1
Fasting glucose≥5.6(mmoll^{-1})	612(21. 9)	20. 4, 23. 4	0(-)	—	110(13. 2)	10. 9, 15. 5	213(34. 5)	30. 8, 38. 2	289(59. 7)	55. 3, 64. 1
Women										
Subtotal	3234（100. 0）	20. 9, 23. 7	837（25. 9）	22. 9, 28. 9	906（28. 0）	26. 5, 29. 5	770（23. 8）	22. 3, 25. 3	721（22. 3）	20. 9, 23. 7
Metabolic abnormalities（n,%）										
Waist circumference≥80（cm）	1599(49. 4)	47. 7, 51. 1	0(-)	—	396(43. 7)	40. 5, 46. 9	555(72. 1)	68. 9, 75. 3	648(89. 9)	87. 7, 92. 1
Triglyceride≥≥1.7(mmoll^{-1})	793(24. 5)	23. 0, 26. 0	0(-)	—	78(8. 6)	6. 8, 10. 4	209(27. 1)	24. 0, 30. 2	506(70. 2)	66. 9, 73. 5
HDL-cholesterol＜1.3(mmoll^{-1})	1002(31. 0)	29. 4, 32. 6	0(-)	—	191(21. 1)	18. 4, 23. 8	297(38. 6)	35. 2, 42. 0	514(71. 3)	68. 0, 74. 6
Blood pressure≥130/85(mmHg	964(29. 8)	28. 2, 31. 4	0(-)	—	162(17. 9)	15. 4, 20. 4	324(42. 1)	38. 6, 45. 6	478(66. 3)	62. 8, 69. 8
Fasting glucose≥5.6(mmoll^{-1})	594(18. 4)	17. 1, 19. 7	0(-)	—	79(8. 7)	6. 9, 10. 5	155(20. 1)	17. 3, 22. 9	360(49. 9)	46. 3, 53. 5

For peer review only

For peer review only - http://bmjopen.bmj.com/site/about/guidelines.xhtml

Table 3 Energy and multivariate adjusted[a,b] mean food and nutrient intake according to the number of metabolic syndrome (MetS)components in men (n=2800)

	Number of MetS components					
Variable	0	1	2	3–5	P^c	Trend P
n，%	866（30. 9%）	832（29. 7%）	618（22. 1%）	484（17. 3%）		
Energy	2349. 9±20. 8	2317. 5±21. 3	2296. 7±24. 6	2269. 6±27. 8	0. 109	0. 016
Nutrients[a]						
Protein(energy %)	12. 3±0. 1	12. 6±0. 1	12. 6±0. 1*	12. 8±0. 1**	0. 001	0. 0001
Fat(energy %)	31. 7±0. 4	31. 6±0. 4	32. 1±0. 4	31. 9±0. 5	0. 748	0. 558
Carbohydrate(energy %)	55. 8±0. 4	55. 7±0. 4	55. 1±0. 4	55. 0±0. 5	0. 460	0. 141
Nutrients[b]						
Protein(g)	70. 4±0. 6	71. 5±0. 6	72. 1±0. 6	73. 4±0. 7**	0. 006	0. 0004
Beans(protein %)	6. 6±0. 3	6. 2±0. 3	6. 4±0. 3	6. 5±0. 4	0. 849	0. 935
Animal foods(protein %)	28. 6±0. 6	29. 9±0. 6	31. 0±0. 6**	30. 4±0. 7	0. 021	0. 020
Other vegetal food (protein %)	62. 9±0. 6	61. 1±0. 6	60. 3±0. 7*	60. 5±0. 8*	0. 011	0. 007
Fat(g)	80. 8±1. 0	80. 8±1. 0	82. 1±1. 1	81. 2±1. 2	0. 790	0. 595
Carbohydrate(g)	321. 0±2. 3	319. 1±2. 3	314. 7±2. 6	317. 7±2. 9	0. 263	0. 192
ß-Carotene(μg)	1493. 6±42. 8	1431. 6±42. 1	1485. 1±47. 4	1472. 0±53. 4	0. 698	0. 957
Vitamin C(mg)	78. 3±1. 4	76. 2±1. 4	76. 6±1. 6	75. 6±1. 8	0. 597	0. 275
Vitamin E(mg)	34. 7±0. 6	34. 3±0. 6	33. 8±0. 7	33. 9±0. 8	0. 744	0. 351
Calcium(mg)	369. 8±5. 3	371. 8±5. 3	371. 5±5. 9	375. 9±6. 7	0. 903	0. 484
Sodium (mg)	4545. 9±79. 2	4563. 3±78. 7	4681. 6±88. 5	4634. 7±99. 2	0. 612	0. 318
Iron(mg)	23. 2±0. 3	23. 6±0. 3	23. 6±0. 4	24. 2±0. 4	0. 184	0. 044
Magnesium(mg)	321. 9±3. 1	322. 8±3	316. 8±3. 4	320. 2±3. 9	0. 547	0. 460
Dietary fiber(g)	11. 7±0. 2	11. 3±0. 2	11. 2±0. 2	11. 5±0. 2	0. 058	0. 419
Cholesterol(mg)	238. 7±6. 3	264±6. 2**	267. 8±7. 0**	266. 6±7. 8*	0. 002	0. 004
Foods[b]						
Cereals(g)	453. 9±4. 9	443. 1±4. 9	437. 1±5. 5*	440. 8±6. 2	0. 083	0. 058
Rice(g)	234. 5±4. 7	233. 9±4. 6	223. 9±5. 2	229. 8±5. 9	0. 376	0. 294
Wheat(g)	159. 5±4. 7	153. 7±4. 6	157. 2±5. 2	155. 8±5. 8	0. 812	0. 741
Coarse cereals(g)	16. 5±1. 4	16. 0±1. 4	14. 1±1. 6	12. 7±1. 8	0. 267	0. 051
Tubers(g)	37. 3±1. 9	39. 1±1. 9	32. 9±2. 1	32. 7±2. 4	0. 048	0. 030
Nuts(g)	3. 2±0. 5	3. 5±0. 5	3. 4±0. 5	4. 1±0. 6	0. 599	0. 207
Beans(g)	17. 0±0. 9	17. 3±0. 9	17. 3±1. 1	19. 5±1. 2	0. 362	0. 113
Vegetables(g)	335. 4±5. 6	327. 9±5. 6	328. 7±6. 3	320. 8±7. 1	0. 408	0. 119
Fruits(g)	54. 0±3. 2	50. 0±3. 2	46. 9±3. 6	48. 5±4. 1	0. 444	0. 212
Fish/seafood (g)	30. 4±2. 1	34. 2±2. 1	33. 8±2. 3	42. 3±2. 6**	0. 003	0. 001
Meats(g)	90. 6±2. 9	95. 7±2. 8	105. 7±3. 2**	98. 4±3. 6	0. 003	0. 016
Abdominal organs (g)	2. 9±0. 5	3. 7±0. 5	4. 1±0. 6	4. 4±0. 6	0. 213	0. 050
Eggs(g)	30. 8±1. 2	33. 3±1. 2	33. 3±1. 4	31. 4±1. 5	0. 318	0. 749
Milk and dairy products(g)	9. 4±1. 4	9. 7±1. 4	11. 9±1. 6	9. 2±1. 8	0. 576	0. 840
Fats_oils(g)	40. 7±0. 9	40. 7±0. 9	40. 4±1	40. 1±1. 1	0. 963	0. 608

For peer review only

For peer review only - http://bmjopen.bmj.com/site/about/guidelines.xhtml

Values shown are mean ± s.e.

[a]Adjusted for age, alcohol intake, smoking ,physical activity, per capita annual income, education level, residences(urban/rural) and geographical regions.

[b]Adjusted for age, energy intake, alcohol intake, smoking, physical activity, per capita annual income, education level, residences(urban/rural) and geographical regions.

[c]Statistical significance was determined by analysis of covariance.

Compared with Number of MetS components 0 group,*$p < 0.05$, **$p < 0.01$.

For peer review only

For peer review only - http://bmjopen.bmj.com/site/about/guidelines.xhtml

Table 4 Energy and multivariate adjusted[a,b] mean food and nutrient intake according to the number of metabolic syndrome (MetS)components in women (n=3234)

	Number of MetS components					
Variable	0	1	2	3-5	P^c	Trend P
n，%	837（25.9%）	906（28.0%）	770（23.8%）	721（22.3%）		
Energy	1972.5±18.8	1966±18.0	1974±19.5	1938.3±20.1	0.549	0.275
Nutrients[a]						
Protein(energy %)	12.7±0.2	12.7±0.2	12.8±0.2	12.8±0.2	0.922	0.567
Fat(energy %)	33.2±0.6	33.4±0.6	33.4±0.7	33.3±0.7	0.979	0.826
Carbohydrate(energy %)	53.9±0.6	53.6±0.6	53.6±0.7	53.7±0.7	0.909	0.715
Nutrients[b]						
Protein(g)	61.0±0.8	61.1±0.8	61.1±0.8	61.4±0.8	0.943	0.583
Beans(protein %)	6.9±0.6	7.2±0.6	7.5±0.6	7.6±0.6	0.449	0.121
Animal foods(protein %)	32.7±1.0	32.3±0.9	32.4±1.0	32.3±1.0	0.929	0.612
Other vegetal food (protein %)	58.6±1.0	59.0±1.0	58.3±1.1	58.5±1.1	0.840	0.695
Fat(g)	72.3±1.4	72.6±1.4	72.5±1.4	72.6±1.5	0.993	0.819
Carbohydrate(g)	259.7±3.2	257.7±3.2	258±3.3	258.5±3.3	0.866	0.689
ß-Carotene(μg)	1578.6±68.0	1391.4±66.9**	1476.5±69.5	1382.7±70.1**	0.001	0.007
Vitamin C(mg)	75.4±2.3	73.9±2.2	76.9±2.3	74.0±2.3	0.292	0.857
Vitamin E(mg)	31.6±0.9	31.9±0.9	32.9±0.9	32.1±0.9	0.309	0.260
Calcium(mg)	353.4±8.4	351.7±8.3	355.5±8.6	342.8±8.7	0.299	0.225
Sodium (mg)	3862.3±116.3	3984.6±114.1	4240.8±118.7**	4005.7±119.0	0.001	0.032
Iron(mg)	19.8±0.5	20.2±0.5	20.2±0.5	19.8±0.5	0.505	0.817
Magnesium(mg)	277.3±4.9	280.9±4.8	278.4±5.0	279.6±5.1	0.804	0.748
Dietary fiber(g)	10.3±0.2	10.6±0.2	10.4±0.2	10.3±0.2	0.555	0.814
Cholesterol(mg)	236.7±10.1	237.6±10	243.7±10.3	245.9±10.4	0.640	0.219
Foods[b]						
Cereals(g)	345.3±6.7	347.8±6.5	346.4±6.8	351.6±6.8	0.440	0.335
Rice(g)	216.4±7.3	207.7±7.1	211.1±7.4	205.5±7.5	0.293	0.139
Wheat(g)	101.9±6.4	106.1±6.3	106.1±6.5	112.6±6.5	0.280	0.066
Coarse cereals(g)	16.1±2.1	15.3±2.1	13.5±2.2	15.8±2.2	0.440	0.646
Tubers(g)	39.8±2.9	38.0±2.9	38.8±3.0	35.2±3.0	0.315	0.112
Nuts(g)	3.7±0.6	3.0±0.6	3.8±0.7	3.4±0.7	0.304	0.907
Beans(g)	15.6±1.4	15.7±1.4	16.6±1.4	16.7±1.4	0.691	0.274
Vegetables(g)	302.2±8.3	303.3±8.1	302.6±8.4	298.2±8.5	0.884	0.583
Fruits(g)	77.9±6.3	76.1±6.2	70.5±6.4	73.5±6.5	0.497	0.268
Fish/seafood (g)	37.3±2.9	34.9±2.8	36.8±2.9	37.1±3.0	0.670	0.886
Meats(g)	86.6±3.7	85.7±3.7	81.0±3.8	84.0±3.8	0.275	0.223
Abdominal organs (g)	3.4±0.7	4.5±0.7	4.1±0.8	4.3±0.8	0.284	0.251
Eggs(g)	28.3±1.8	27.2±1.7	28.6±1.8	27.6±1.8	0.714	0.903
Milk and dairy products(g)	17.8±2.5	14.9±2.4	13.1±2.5	11.5±2.5**	0.020	0.002
Fats_oils(g)	34.7±1.2	35.0±1.2	36.0±1.2	36.0±1.2	0.459	0.133

For peer review only

Values shown are mean ± s.e.

[a]Adjusted for age, alcohol intake, smoking ,physical activity, per capita annual income, education level, residences(urban/rural) and geographical regions.

[b]Adjusted for age, energy intake, alcohol intake, smoking, physical activity, per capita annual income, education level, residences(urban/rural) and geographical regions.

[c]Statistical significance was determined by analysis of covariance.

Compared with Number of MetS components 0 group,*$p < 0.05$, **$p < 0.01$.

For peer review only

For peer review only - http://bmjopen.bmj.com/site/about/guidelines.xhtml

STROBE Statement—checklist of items that should be included in reports of observational studies

	Item No	Recommendation
**abstract(page 1)** **Title(page2)**	1	(*a*) Indicate the study's design with a commonly used term in the title or the abstract
		(*b*) Provide in the abstract an informative and balanced summary of what was done and what was found
**Introduction** (page 3 line 19—page 5 line 8 in Main Document)		
Background/rationale (page 3 line 19—page 4 line 54 in Main Document)	2	Explain the scientific background and rationale for the investigation being reported
Objectives (page 4 line 55—page 5 line 33 in Main Document)	3	State specific objectives, including any prespecified hypotheses
**Methods** (page 5 line 13—page 8 line 20 in Main Document)		
Study design (page 5 line 16—page 5 line 33 in Main Document)	4	Present key elements of study design early in the paper
Setting (page 5 line 48—page 6 line 40 in Main Document)	5	Describe the setting, locations, and relevant dates, including periods of recruitment, exposure, follow-up, and data collection
Participants (page 5 line 35—page 5 line 44 in Main Document)	6	(*a*) *Cohort study*—Give the eligibility criteria, and the sources and methods of selection of participants. Describe methods of follow-up *Case-control study*—Give the eligibility criteria, and the sources and methods of case ascertainment and control selection. Give the rationale for the choice of cases and controls *Cross-sectional study*—Give the eligibility criteria, and the sources and methods of selection of participants
		(*b*) *Cohort study*—For matched studies, give matching criteria and number of exposed and unexposed *Case-control study*—For matched studies, give matching criteria and the number of controls per case
Variables (page 6 line 44—page 7 line 36 in Main Document)	7	Clearly define all outcomes, exposures, predictors, potential confounders, and effect modifiers. Give diagnostic criteria, if applicable
Data sources/ measurement (page 5 line 48—page 6 line 40 in Main Document)	8*	For each variable of interest, give sources of data and details of methods of assessment (measurement). Describe comparability of assessment methods if there is more than one group
Bias (page 7 line 8—page 7 line 36 in Main Document)	9	Describe any efforts to address potential sources of bias
Study size (page 5 line 35—page 5 line 44 in Main Document)	10	Explain how the study size was arrived at
Quantitative variables (page 7 line 52—page 7 line	11	Explain how quantitative variables were handled in the analyses. If applicable, describe which groupings were chosen and why

For peer review only - http://bmjopen.bmj.com/site/about/guidelines.xhtml

55 in Main Document)		
Statistical methods (page 7 line 41—page 8 line 19 in Main Document)	12	(*a*) Describe all statistical methods, including those used to control for confounding
		(*b*) Describe any methods used to examine subgroups and interactions
		(*c*) Explain how missing data were addressed
		(*d*) *Cohort study*—If applicable, explain how loss to follow-up was addressed<br>*Case-control study*—If applicable, explain how matching of cases and controls was addressed<br>*Cross-sectional study*—If applicable, describe analytical methods taking account of sampling strategy
		(*e*) Describe any sensitivity analyses

Continued on next page

For peer review only

For peer review only - http://bmjopen.bmj.com/site/about/guidelines.xhtml

For peer review only

**Results**(page 8 line 23—page 10 line 21 in Main Document)			
Participants<br>（This is observational studies）	13*		(a) Report numbers of individuals at each stage of study—eg numbers potentially eligible, examined for eligibility, confirmed eligible, included in the study, completing follow-up, and analysed
			(b) Give reasons for non-participation at each stage
			(c) Consider use of a flow diagram
Descriptive data<br>（This is observational studies）	14*		(a) Give characteristics of study participants (eg demographic, clinical, social) and information on exposures and potential confounders
			(b) Indicate number of participants with missing data for each variable of interest
			(c) *Cohort study*—Summarise follow-up time (eg, average and total amount)
Outcome data<br>（This is observational studies）	15*		*Cohort study*—Report numbers of outcome events or summary measures over time
			Case-control study—Report numbers in each exposure category, or summary measures of exposure
			Cross-sectional study—Report numbers of outcome events or summary measures
Main results<br>(page8 line 23—page 10 line 21 in Main Document)	16		(*a*) Give unadjusted estimates and, if applicable, confounder-adjusted estimates and their precision (eg, 95% confidence interval). Make clear which confounders were adjusted for and why they were included
			(*b*) Report category boundaries when continuous variables were categorized
			(*c*) If relevant, consider translating estimates of relative risk into absolute risk for a meaningful time period
Other analyses<br>(page 9 line 10—page 10 line 21 in Main Document)	17		Report other analyses done—eg analyses of subgroups and interactions, and sensitivity analyses
**Discussion**(page 10 line 27—page 15 line 34 in Main Document)			
Key results<br>(page 10 line 27—page 10 line 41，page 15 line 22—page 15 line 34 in Main Document)	18		Summarise key results with reference to study objectives
Limitations<br>（page 14 line 48—page 15 line 19）	19		Discuss limitations of the study, taking into account sources of potential bias or imprecision. Discuss both direction and magnitude of any potential bias
Interpretation<br>(page 10 line 43—page 14 line 46 in Main Document)	20		Give a cautious overall interpretation of results considering objectives, limitations, multiplicity of analyses, results from similar studies, and other relevant evidence
Generalisability<br>（page 15 line 22—page 15 line 34 in Main Document）	21		Discuss the generalisability (external validity) of the study results
**Other information**(page 15 line 41—page 15 line 55 in Main Document)			
Funding<br>(page 15 line 47—page 15 line 48 in Main Document)	22		Give the source of funding and the role of the funders for the present study and, if applicable, for the original study on which the present article is based

For peer review only - http://bmjopen.bmj.com/site/about/guidelines.xhtml

*Give information separately for cases and controls in case-control studies and, if applicable, for exposed and unexposed groups in cohort and cross-sectional studies.

Note: An Explanation and Elaboration article discusses each checklist item and gives methodological background and published examples of transparent reporting. The STROBE checklist is best used in conjunction with this article (freely available on the Web sites of PLoS Medicine at http://www.plosmedicine.org/, Annals of Internal Medicine at http://www.annals.org/, and Epidemiology at http://www.epidem.com/). Information on the STROBE Initiative is available at www.strobe-statement.org.

For peer review only

For peer review only - http://bmjopen.bmj.com/site/about/guidelines.xhtml

BMJ Open

Relationship between dietary factors and the number of altered metabolic syndrome components in Chinese adults: a cross-sectional study from the China Health and Nutrition Survey

Journal:	*BMJ Open*
Manuscript ID	bmjopen-2016-014911.R1
Article Type:	Research
Date Submitted by the Author:	31-Jan-2017

Complete List of Authors:	Cheng, Maowei; National Institute for Nutrition and Health, Chinese Center for Disease Control and Prevention; Center for Disease Control and Prevention of Hubei Province<br>Wang, Huijun ; National Institute for Nutrition and Health, Chinese Center for Disease Control and Prevention<br>Wang, Zhihong ; National Institute for Nutrition and Health, Chinese Center for Disease Control and Prevention<br>Du, Wenwen ; National Institute for Nutrition and Health, Chinese Center for Disease Control and Prevention<br>Ouyang, Yifei ; National Institute for Nutrition and Health, Chinese Center for Disease Control and Prevention<br>Zhang, Bing; National Institute for Nutrition and Health, Chinese Center for Disease Control and Prevention

**Primary Subject Heading**:	Public health
Secondary Subject Heading:	Nutrition and metabolism
Keywords:	Adults, Chinese, Components, Dietary factors, Metabolic syndrome

SCHOLARONE™ Manuscripts

For peer review only

Relationship between dietary factors and the number of altered metabolic syndrome components in Chinese adults: a cross-sectional study from the China Health and Nutrition Survey

Maowei Cheng[1,2], Huijun Wang[1], Zhihong Wang[1], Wenwen Du[1], Yifei Ouyang[1], and Bing Zhang[1,*]

[1]National Institute for Nutrition and Health, Chinese Center for Disease Control and Prevention, Beijing, China

[2] Center for Disease Control and Prevention of Hubei Province, Wuhan, China

[*] **Corresponding author:** Bing Zhang

E-mail: zzhangb327@aliyun.com

Postal address: No.27 Nan Wei Rd., Xi Cheng District, Beijing, zip code:100050

Telephone: 010-83151561 (office), 18601109679 (Mobile phone)

Fax numbers: 010-67711813

Keywords: Adults; Chinese; Components; Dietary factors; Metabolic syndrome

Word count: 4738 words

For peer review only

Abstract

Objectives: This study aims to study the correlation between dietary factors and the number of altered MetS components in Chinese adults systematically.

Setting: A cross-sectional study using demographic and dietary data of adults aged 18-75 years from the China Health and Nutrition (2009) was conducted in nine provinces in China.

Participants: There were 6034 eligible subjects (2800 men and 3234 women) in this study.

Outcomes: The primary outcome of this study was diet assessments and the number of altered MetS components. Dietary intake was measured using a combination of 3 day period with 24-hour and household food inventory, and average daily intakes of nutrients were estimated according to the Chinese Food Composition Table. Blood samples were analyzed in a national central lab and the number of clustering MetS components was calculated by adding the presence of each MetS component.

Results: After adjusting for covariates and taking zero MetS as comparison, the high risk factors correlating with increased numbers of altered MetS components in men were higher intake of protein (70.4–73.4g; *P* trend =0.0004), cholesterol (238.7–266.6mg; *P* trend =0.004), meat (90.6–105.7g; *P* trend =0.016), fish/seafood (30.4–42.3g; *P* trend =0.001), and lower intake of coarse cereals (16.5–12.7g; *P* trend = 0.051), tubers (37.3–32.7g; *P* trend = 0.030), and dietary fiber (11.7–11.5 g; ANCOVA *P*= 0.058). Meanwhile, the high risk factors correlating with increased number of altered MetS components in women were higher intake of wheat (101.9–112.6g; *P* trend =0.066) and sodium (3862.3–4005.7mg, *P* trend =0.032); , and lower intake of ß-carotene (1578.6–1382.7μg; *P* trend =0.007), milk, and dairy products (17.8–11.5g; *P* trend =0.002).

Conclusions: Some foods and nutritional factors correlates with increased numbers of altered MetS components in Chinese adults. More clinical research works to examine these associations are underway.

For peer review only - http://bmjopen.bmj.com/site/about/guidelines.xhtml

Strengths and limitations of this study

1. This is the first observational study to examine relations between dietary factors and the number of altered MetS components among Chinese adults in a systematic way.
2. This study include the large sample size, fairly representative of the Chinese population. Furthermore, only healthy adults, not using chronic medication were included.
3. In this study we measured diet using a combination of three consecutive days by 24-hour dietary recall and a household inventory. One of the prominent advantages of this method is that combine 24-hour individual recall with edible oil and other condiments consumption measured by household inventory, which improve the quanlity of individual dietary data greatly.
4. The major limitation of this study is the inability to come to any causal conclusion due to the cross-sectional design.

INTRODUCTION

Metabolic syndrome (MetS) is a cluster of risk factors (dysglycemia, elevated blood pressure, dyslipidaemia, and central adiposity) that has been shown to be predictive of cardiovascular disease and diabetes.[1]

The overall age-standardized estimates of MetS prevalence in Chinese adults was 18.2%, according to the International Diabetes Federation, and 21.3%, according to the modified Adult Treatment Panel III criteria of the National Cholesterol Education Program (NCEP-ATP III). These estimates of MetS prevalence increase gradually with age.[2] Therefore, MetS is considered as a major challenge that affects the quality of life of millions of people. It is urgent to identify effective strategies to better control MetS.

MetS requires 3 of 5 components: waist circumference (WC), fasting glucose, high density lipoprotein cholesterol (HDL-C), triglycerides, and hypertension[1].MetS in individual patients, may involve the alteration of various combinations of these components. Accumulating evidence has suggested that an increased number of altered MetS components is strongly associated with a higher risk of developing diseases.[3–6] Serum magnesium level decreased as the number of altered MetS components increased in elderly Greek patients[5]. The number of altered MetS components in

For peer review only - http://bmjopen.bmj.com/site/about/guidelines.xhtml

middle-aged and elderly Japanese patients was related to lower intake of vitamin B_6,[6] and lower dietary fiber in men, and to lower intake of calcium, milk, dairy products, and higher cereal intake in women, after adjusting for age, energy intake, alcohol intake, smoking status, physical activity.

An increasing number of studies in China have focused on the influence of dietary patterns on the prevalence of MetS and its altered components. Using data from the 2002 China National Nutrition and Health Survey (CNNHS), three types of dietary patterns have been identified in the Chinese population. One dietary pattern is called “Yellow Earth” or “traditional northern” pattern; it is characterized by consumption of refined cereal products, salted vegetables and tubers. Another dietary pattern is called “Western/new affluence”; it is characterized by consumption of beef, lamb, milk, cheese, and yogurt. The third dietary pattern is called “Green Water” or “traditional southern” pattern; it is characterized by consumption of rice, vegetables, and moderate amounts of animal foods.[7–9] The “Yellow Earth” pattern and the “Western/new affluence” pattern were associated with an increased likelihood of MetS, or with increased alterations of its components,[8 9] in comparison to the “Green Water” dietary pattern. Our previous studies have identified a modern high-wheat pattern that is characterized by consumption of wheat products, nuts, fruits, eggs, milk and instant noodles or frozen dumplings. This modern pattern could be considered as a combination of the “Yellow Earth” and “Western/new affluence” patterns, and has been positively associated with diabetes.[10 11]Additionally, in our previous studies an “alcohol” dietary pattern was identified among men. This dietary pattern is characterized by consumption of meat, alcohol, and nuts, and has been positively associated with overweight/obesity and central obesity.[12]

However, it would be difficult to obtain an identical relationship between dietary patterns (or dietary index) and the relevant disease outcomes in different studies[13]. More importantly, because diet is a complex variable, it is necessary to adopt multiple approaches to examine the relationship between diet and the risk of diseases. If there were any effects caused by a particular nutrient, the dietary pattern approach would not be optimal to examine the relationship between diet and risk of diseases because the effect of that nutrient would be diluted by other factors.[14]

There are significant differences in the dietary characteristics of men and women.[15 16] To our knowledge, the nutrients that could have a positive effect on MetS or on its components have not been determined in men and women, and no studies have explored the association between dietary factors and the number of altered MetS components in the Chinese population. The aim of our

For peer review only - http://bmjopen.bmj.com/site/about/guidelines.xhtml

study was to examine the relationship between dietary factors and the number of altered MetS components in a systematic way, using a large sample of Chinese adults (N = 6034).

METHODS

Study population

We used the data collected by the China Health and Nutrition Surveys (CHNS) 2009— an ongoing longitudinal survey, which was designed to examine the association among economic and social changes and a range of health behaviors across space and time.[10] A multistage, random cluster sampling method was used to select the samples from nine provinces (Liaoning, Heilongjiang, Jiangsu, Shandong, Henan, Hubei, Hunan, Guangxi and Guizhou) in China. Questionnaire information was used to collect the data as follows: social and economic factors, health factors, nutrition factors and population factors of sociology. However, blood samples were collected only at the first time in 2009. More details about the CHNS data have been described elsewhere.[17]

In 2009, there were a total of 7755 respondents (3589 men and 4166 women) with ages of ⩾ 18 years, individuals with incomplete dietary data (n=296), over 75 years of age (n=419), suffering from chronic diseases (hypertension, diabetes, stroke, myocardial infarction) with a change in diet or physical and/or pharmacological treatment (n=906), and women in pregnancy or lactation status (n=100) were excluded. Finally, a total of 6034 adults (2800 men and 3234 women) were included in this analysis.

Diet assessments

The 2009 CHNS combined three consecutive days by 24-hour dietary recall and a household inventory to assess individual consumption. Individual dietary intake for three consecutive days (two weekdays and one weekend day) was collected for every household member. Food items consumed at restaurants, canteens and other locations away from home were systematically recorded. Using food models and pictures, trained field interviewers recorded the amounts of all foods and beverage items (measured in grams) consumed during 24 h of the previous day[18 19]. In addition, household food intake was determined on a daily basis by calculating the changes in food inventory. Chinese balance with a maximum limit of 15 kg and a minimum of 20 g was used to

For peer review only

measure household consumption by inventory change from the beginning to the end of each day. At the same time, all foods and condiments in home inventory, purchased from markets, picked from gardens and food waste were carefully recorded at the start and end of each survey[20]. The full list of food groups using by a household inventory have been described in supplemental table 1 and elsewhere.[11]

Individual daily intake value for each food item was assessed using data from 24-hour dietary recall, which was enhanced by use of data from a household measure. Meanwhile, edible oils as well as other common condiments (sugar, starch soya sauce, salt) consumed in the household by each member was allocated based on the proportion of reference men.

The amount of nutrients for each food was available from the Chinese Food Composition Table (2nd edition) [21]. Per capita daily nutrients were calculated by combining both of these. Twelve nutrients, 13 foods, percentage of energy from protein and fat, and percentage of protein from various foods were selected as diet indicators according to previous studies[6 22 23].

Other measurements

Weight, height and waist circumference were measured by trained surveyors using standard measurement techniques: Height was measured without shoes to the nearest 0.2cm using a portable stadiometer. Weight was measured without shoes and in light clothing to the nearest 0.1kg on a calibrated beam scale.[24] Between the lowest rib and the iliac crest in a horizontal plane, waist circumference was measured at a point midway using non-elastictape.[25]According to a standard protocol, blood pressure was measured by trained surveyors using a mercury sphygmomanometer.[26]

Blood samples were collected by vein puncture after an overnight fast. All samples were analyzed in a national central lab in Beijing (medical laboratory accreditation certificate ISO 15189:2007) under strict quality control. Fasting plasma glucose was measured with a glucose oxidase phenol 4-aminoantipyrine peroxidase kit (Randox, Crumlin, UK) in a Hitachi 7600 automated analyzer (Hitachi Inc., Tokyo, Japan).[11 27] High-density lipoprotein cholesterol (HDL-C) and triglycerides (TG) were both measured by their corresponding reagents (Kyowa MedexCo., Ltd., Tokyo, Japan) using glycerol-phosphate oxidase method, and the polyethylene glycol modified enzyme method, respectively. All lipid measurements were carried out on the Hitachi 7600 automated analyzer (Hitachi Inc., Tokyo, Japan).[28] History of chronic patients (past/present),

For peer review only - http://bmjopen.bmj.com/site/about/guidelines.xhtml

For peer review only

smoking drinking (y/n) and the occupational physical activity were collected using questionnaire.

Definition of MetS and its components

The definition of MetS was based on the most recent Joint Interim Statement (JIS) criteria.[29] As mentioned earlier, subjects under treatment for chronic diseases (hypertension, diabetes, stroke, myocardial infarction) were excluded in present study. Thus, MetS was defined as the presence of three or more of the following five components: (1)abdominal obesity, WC men⩾90cm and women⩾80cm specifically for Asia adults;[6] (2)elevated blood pressure, SBP/DBP⩾130/85mmHg; (3) hypertriglyceridemia, triglyceride⩾1.70mmoll^{-1}; (4) low high-density lipoprotein cholesterol (HDL-C), HDL-C＜1.0mmoll^{-1} in men and＜1.3 mmoll^{-1} in women; and (5) elevated blood glucose levels, fasting blood glucose⩾5.6 mmoll^{-1}.The number of clustering MetS components was calculated by adding the number of MetS components.[6]

Definition of covariates

According to the definition of WHO, the adults who smoked at least one cigarette a day currently were defined as current smoker.[12] For drinker, individuals were asked questions: "Have you consumed alcohol (beer, wine or other alcoholic beverage) during the past year (yes, no)?".[2] Residence was divided into urban and rural and geographical region was divided into the North (Liaoning, Heilongjiang, Henan and Shandong) and the South (Hunan, Hubei, Jiangsu, Guangxi and Guizhou).[12] BMI was calculated as weight in kilograms divided by the square of height in meters.

Current economic status was assessed by mean per capita annual income (unit: RMB Yuan) in the year before the 2009 CHNS. The subjects were classified as low, moderate, and high income level by trisected percentiles of the mean per capita annual income. Participants were interviewed using a semi-quantitative assessment to determine their occupational, domestic, travel, and leisure physical activity levels. The intensity (metabolic equivalent, METs, unit kcal/kg h) of each activity in the questionnaire was coded according to the compendium of physical activities.[30] The total metabolic equivalent (MET-hours/week) was a combined score calculated by multiplying the frequency, duration, and intensity of physical activity. Total metabolic equivalent scores were categorized into three levels (mild, moderate, and high) by trisected percentiles for further analysis.

For peer review only - http://bmjopen.bmj.com/site/about/guidelines.xhtml

Statistical analysis

Statistical Analysis System 9.2 (SAS Institute, Cary, NC, USA) was used for all statistical analyses. Subjects were categorized into four groups according to the number of clustering MetS components (0, 1, 2, 3-5).Values of three to five clustering MetS components were combined because only a few subjects had alterations in four or five MetS components (four altered components: 121 men and 215 women; five altered components: 28 men and 64 women). Associations between categorical variables were tested using Chi-Square Test. Comparisons between continuous variables were performed by analysis of variance, and 95% confidence intervals (CI) were estimated.

Assessment of model effects and trend tests were performed to analyze the association between each of the dietary indicators and the number of altered MetS components using a generalized linear regression model. Adjustments were made for all potential confounding factors, including age, energy intake, alcohol intake, smoking status, per capita annual income, education levels, physical activity levels, residence (urban/rural) and geographical region (North/South). Mean nutritional intakes were calculated by the number of MetS components (0, 1, 2, 3-5). The estimated marginal means for each of dietary indicators of altered MetS components groups1, 2, and 3-5 were compared with those of MetS components group 0 by multiple comparison test adjusted by Bonferroni method.

All reported *P*-values were estimated using two-sided analysis. A *P*-value <0.05 was considered statistically significant.

RESULTS

Characteristics of the study population

Table 1 shows the characteristics and distribution of all the subjects included in the study categorized by sex. Mean age ± s.d. was 47.3 ± 13.6 years for men, and 47.6 ± 13.1 years for women. Body mass index (BMI) was 23.0 ± 3.1 kgm^{-2} for men, and 23.0 ± 3.2 kgm^{-2} for women. The number of altered MetS components in both men and women gradually increased as age and BMI increased.

We estimated that 58.0% of men currently smoked, and 63.0% consumed alcohol; 3.2% of women smoked, and 9.2% consumed alcohol.

For peer review only - http://bmjopen.bmj.com/site/about/guidelines.xhtml

Overall, there was a positive association between the number of altered MetS components and the proportion of moderate and high per capita annual income, but only in men. In women, the number of altered MetS components was associated with a higher proportion of individuals in primary school and under educational levels. In addition, there was a negative relationship between the number of altered MetS components in men and women and moderate and high physical activity levels.

Table 1 Subject characteristics according to the number of metabolic Syndrome(MetS) components

		Number of MetS components					
	ALL	0	1	2	3–5	P^a	Trend P
Men							
n，%	2800（100.0%）	866（30.9%）	832（29.7%）	618（22.1%）	484（17.3%）		
Age（year）	47.3±13.6	44.0±14.1	48.0±13.7	48.7±12.8	50.2±12.0	<0.001	<0.001
Body mass index (Kgm^{-2})	23.0±3.1	21.2±2.4	22.5±2.8	24.0±2.8	25.7±2.9	<0.001	<0.001
Energy intake（Kcal per day）	2308.4±610.4	2349.9±614.1	2317.5±607.0	2296.7±601.3	2269.6±27.8	0.109	0.016
Urban residents(n,%)	740(26.4)	181(20.9)	204(24.5)	183(29.6)	172(35.5)	<0.001	<0.001
Northern residents(n,%)	1176(42.0)	300(34.6)	342(41.1)	292(47.2)	242(50.0)	<0.001	<0.001
Moderate and high physical activity(n,%)	1674（59.7%）	556(19.9)	516(18.4)	349(12.5)	253(9.0)	<0.001	<0.001
Moderate and high per capita annual income(n,%)	1864（67.1%）	564(20.3)	532(19.2)	424(15.3)	344(12.4)	0.005	0.001
Education level(n,%)							
Primary school and under	876（31.3）	253(9.0)	283(10.1)	197(7.0)	143(5.1)		
Junior middle school	1122（40.1）	369(13.2)	333(11.9)	225(8.0)	195(7.0)	0.062	0.438
High school and above	798（28.5）	243(8.7)	214(7.7)	195(7.0)	146(5.2)		
Drinker(n,%)	1764(63.0%)	525(60.6%)	523(62.9%)	402(65.0%)	314(64.9%)	0.267	0.061
Current smoker（n,%）	1623(58.0%)	516(59.6%)	496(59.6%)	341(55.2%)	270(55.8%)	0.189	0.062
Women							
n，%	3234（100.0%）	837（25.9%）	906（28.0%）	770（23.8%）	721（22.3%）		
Age（year）	47.6±13.1	41.2±12.6	46.4±13.1	50.2±12.0	53.7±11.1	<0.001	<0.001
Body mass index (Kgm^{-2})	23.0±3.2	20.8±2.1	22.3±2.9	24.0±2.9	25.3±3.0	<0.001	<0.001
Energy intake（Kcal per day）	1963.4±535.8	1972.5±522.0	1966±532.7	1974±552.5	1938.3±537.4	0.549	0.275
Urban residents（n,%）	948(29.3)	233(27.8)	267(29.5)	208(27.0)	240(33.3)	0.039	0.068
Northern residents（n,%）	1369(42.3)	279(33.3)	388(42.8)	349(45.3)	353(49.0)	<0.001	<0.001
Moderate and high physical activity（n,%）	1921(59.4%)	553(17.1)	552(17.1)	453(14.0)	363(11.2)	<0.001	<0.001
Moderate and high per capita annual income(n,%)	2119（66.4%）	574(18.0)	581(18.2)	487(15.3)	477(14.9)	0.032	0.616
Education level(n,%)							
Primary school and under	1508（46.7%）	280(8.7)	413(12.8)	406(12.6)	409(12.7)		
Junior middle school	1003（31.0%）	297(9.2)	306(9.5)	213(6.6)	187(5.8)	<0.001	<0.001
High school and above	720（22.3%）	259(8.0)	187(5.8)	150(4.6)	124(3.8)		
Drinker(n,%)	299(9.2%)	90(10.8%)	79(8.7%)	64(8.3%)	66(9.2%)	0.338	0.243
Current smoker（n,%）	105(3.2%)	18(2.2%)	28(3.1%)	26(3.4%)	33(4.6%)	0.061	0.008

Values shown are mean ± s.d.

[a]Statistical significance was determined by analysis of variance or Chi-Square test.

For peer review only - http://bmjopen.bmj.com/site/about/guidelines.xhtml

Prevalence of MetS and metabolic abnormalities

As shown in Table 2, the overall prevalence of MetS was significantly higher in women (22.3%, 95% CI 20.9, 23.7) than in men (17.3%, 95% CI 15.9, 18.7). Significant differences between sexes were noted in the analysis of individual components. Elevated blood pressure, hypertriglyceridemia, and hyperglycemia were more frequent in men than in women. Abdominal obesity and low HDL cholesterol were more frequent in women than in men. In men hypertension was the most prevalent component of MetS (38.6%), followed by hypertriglyceridemia (31.5%). In women, the most prevalent MetS component was abdominal obesity (49.4%), followed by low HDL-C (31.0%).

For peer review only

Table 2 Prevalence of metabolic abnormalities according to the number of metabolic Syndrome(MetS) components

	ALL		Number of MetS components							
			0		1		2		3-5	
	n, %	95% CI	n, %	95% CI	n, %	95% CI	n, %	95% CI	n, %	95% CI
Men	2800（100. 0）	15. 9, 18. 7	866（30. 9）	27. 8, 34. 0	832（29. 7）	28. 0, 31. 4	618（22. 1）	20. 6, 23. 6	484（17. 3）	15. 9, 18. 7
Metabolic abnormalities（n,%）										
Waist circumference≥90（cm）	710(25. 4)	23. 8, 27. 0	0(−)	—	111(13. 3)	11. 0, 15. 6	254(41. 1)	37. 2, 45. 0	345(71. 3)	67. 3, 75. 3
Triglyceride≥1.7(mmoll^{-1})	883(31. 5)	29. 8, 33. 2	0(−)	—	187(22. 5)	19. 7, 25. 3	296(47. 9)	44. 0, 51. 8	400(82. 6)	79. 2, 86. 0
HDL-cholesterol＜1.0(mmoll^{-1})	411(14. 7)	13. 4, 16. 0	0(−)	—	56(6. 7)	5. 0, 8. 4	119(19. 3)	16. 2, 22. 4	236(48. 8)	44. 3, 53. 3
Blood pressure≥130/85(mmHg)	1081(38. 6)	36. 8, 40. 4	0(−)	—	368(44. 2)	40. 8, 47. 6	354(57. 3)	53. 4, 61. 2	359(74. 2)	70. 3, 78. 1
Fasting glucose≥5.6(mmoll^{-1})	612(21. 9)	20. 4, 23. 4	0(−)	—	110(13. 2)	10. 9, 15. 5	213(34. 5)	30. 8, 38. 2	289(59. 7)	55. 3, 64. 1
Women	3234（100. 0）	20. 9, 23. 7	837（25. 9）	22. 9, 28. 9	906（28. 0）	26. 5, 29. 5	770（23. 8）	22. 3, 25. 3	721（22. 3）	20. 9, 23. 7
Metabolic abnormalities（n,%）										
Waist circumference≥80（cm）	1599(49. 4)	47. 7, 51. 1	0(−)	—	396(43. 7)	40. 5, 46. 9	555(72. 1)	68. 9, 75. 3	648(89. 9)	87. 7, 92. 1
Triglyceride≥≥1.7(mmoll^{-1})	793(24. 5)	23. 0, 26. 0	0(−)	—	78(8. 6)	6. 8, 10. 4	209(27. 1)	24. 0, 30. 2	506(70. 2)	66. 9, 73. 5
HDL-cholesterol＜1.3(mmoll^{-1})	1002(31. 0)	29. 4, 32. 6	0(−)	—	191(21. 1)	18. 4, 23. 8	297(38. 6)	35. 2, 42. 0	514(71. 3)	68. 0, 74. 6
Blood pressure≥130/85(mmHg	964(29. 8)	28. 2, 31. 4	0(−)	—	162(17. 9)	15. 4, 20. 4	324(42. 1)	38. 6, 45. 6	478(66. 3)	62. 8, 69. 8
Fasting glucose≥5.6(mmoll^{-1})	594(18. 4)	17. 1, 19. 7	0(−)	—	79(8. 7)	6. 9, 10. 5	155(20. 1)	17. 3, 22. 9	360(49. 9)	46. 3, 53. 5

For peer review only

For peer review only - http://bmjopen.bmj.com/site/about/guidelines.xhtml

Food and nutrient intake in correlation with different MetS components in men

Table 3 shows multivariate adjusted mean food and nutrients intake, according to the number of altered MetS components in men. Higher daily intake of meat (range of intake: 90.6–105.7g) and fish/seafood (30.4–42.3g) was positively correlated with an increased number of altered MetS components (*P* trend =0.016 for meat; *P* trend=0.001 for fish/seafood). Although analysis of covariance or trend tests did not reach statistical significance, the number of altered MetS components were positively correlated with daily intakes of abdominal organs (2.9–4.4g; *P* trend = 0.050), beans (17.0–19.5g; *P* trend= 0.113), and nuts (3.2–4.1g; *P* trend =0.207).

Among the food groups, there was a negative correlation between the number of altered MetS components and the daily intake of cereals (453.9– 440.8g; ANCOVA *P* = 0.083, *P* trend = 0.058), coarse cereals (16.5–12.7g; *P* trend = 0.051), and tubers (37.3–32.7g; ANCOVA *P* = 0.048, *P* trend = 0.030).

An increased number of altered MetS components were positively correlated with a higher daily intake of protein (70.4–73.4g; *P* trend=0.0004) and cholesterol (238.7–266.6mg *P* trend =0.004). It is worth noting that there was a positive correlation between an increased number of altered MetS components and an increased energy daily intake of protein (12.3%–12.8%; ANCOVA *P*=0.001, *P* trend =0.0001), and with increased daily intake of protein of animal origin (28.6%–30.4% ; ANCOVA *P*=0.021, *P* trend =0.020).

Among the dietary indicators, there was a marginally negative difference between the number of altered MetS components and the intake of dietary fiber (11.7–11.5 g; ANCOVA *P*= 0.058). Dietary cholesterol intake in the groups of MetS components1, 2 and 3-5 were significantly higher than in the group 0 (ANCOVA *P*=0.002; multiple comparison test *P*=0.006, *P*=0.003, *P*=0.012).

For peer review only - http://bmjopen.bmj.com/site/about/guidelines.xhtml

Table 3 Energy and multivariate adjusted[a,b] mean food and nutrient intake according to the number of metabolic syndrome (MetS)components in men (N=2800)

	Number of MetS components					
	0	1	2	3–5	P^c	Trend P
n，%	866（30.9%）	832（29.7%）	618（22.1%）	484（17.3%）		
Energy	2349.9±20.8	2317.5±21.3	2296.7±24.6	2269.6±27.8	0.109	0.016
Nutrients[a]						
Protein(energy %)	12.3±0.1	12.6±0.1	12.6±0.1*	12.8±0.1**	0.001	0.0001
Fat(energy %)	31.7±0.4	31.6±0.4	32.1±0.4	31.9±0.5	0.748	0.558
Carbohydrate(energy %)	55.8±0.4	55.7±0.4	55.1±0.4	55.0±0.5	0.460	0.141
Nutrients[b]						
Protein(g)	70.4±0.6	71.5±0.6	72.1±0.6	73.4±0.7**	0.006	0.0004
Beans(protein %)	6.6±0.3	6.2±0.3	6.4±0.3	6.5±0.4	0.849	0.935
Animal foods(protein %)	28.6±0.6	29.9±0.6	31.0±0.6**	30.4±0.7	0.021	0.020
Other vegetal food (protein %)	62.9±0.6	61.1±0.6	60.3±0.7*	60.5±0.8*	0.011	0.007
Fat(g)	80.8±1.0	80.8±1.0	82.1±1.1	81.2±1.2	0.790	0.595
Carbohydrate(g)	321.0±2.3	319.1±2.3	314.7±2.6	317.7±2.9	0.263	0.192
ß-Carotene(μg)	1493.6±42.8	1431.6±42.1	1485.1±47.4	1472.0±53.4	0.698	0.957
Vitamin C(mg)	78.3±1.4	76.2±1.4	76.6±1.6	75.6±1.8	0.597	0.275
Vitamin E(mg)	34.7±0.6	34.3±0.6	33.8±0.7	33.9±0.8	0.744	0.351
Calcium(mg)	369.8±5.3	371.8±5.3	371.5±5.9	375.9±6.7	0.903	0.484
Sodium (mg)	4545.9±79.2	4563.3±78.7	4681.6±88.5	4634.7±99.2	0.612	0.318
Iron(mg)	23.2±0.3	23.6±0.3	23.6±0.4	24.2±0.4	0.184	0.044
Magnesium(mg)	321.9±3.1	322.8±3	316.8±3.4	320.2±3.9	0.547	0.460
Dietary fiber(g)	11.7±0.2	11.3±0.2	11.2±0.2	11.5±0.2	0.058	0.419
Cholesterol(mg)	238.7±6.3	264.0±6.2**	267.8±7.0**	266.6±7.8*	0.002	0.004
Foods[b]						
Cereals(g)	453.9±4.9	443.1±4.9	437.1±5.5*	440.8±6.2	0.083	0.058
Rice(g)	234.5±4.7	233.9±4.6	223.9±5.2	229.8±5.9	0.376	0.294
Wheat(g)	159.5±4.7	153.7±4.6	157.2±5.2	155.8±5.8	0.812	0.741
Coarse cereals(g)	16.5±1.4	16.0±1.4	14.1±1.6	12.7±1.8	0.267	0.051
Tubers(g)	37.3±1.9	39.1±1.9	32.9±2.1	32.7±2.4	0.048	0.030
Nuts(g)	3.2±0.5	3.5±0.5	3.4±0.5	4.1±0.6	0.599	0.207
Beans(g)	17.0±0.9	17.3±0.9	17.3±1.1	19.5±1.2	0.362	0.113
Vegetables(g)	335.4±5.6	327.9±5.6	328.7±6.3	320.8±7.1	0.408	0.119
Fruits(g)	54.0±3.2	50.0±3.2	46.9±3.6	48.5±4.1	0.444	0.212
Fish/seafood (g)	30.4±2.1	34.2±2.1	33.8±2.3	42.3±2.6**	0.003	0.001
Meats(g)	90.6±2.9	95.7±2.8	105.7±3.2**	98.4±3.6	0.003	0.016
Abdominal organs (g)	2.9±0.5	3.7±0.5	4.1±0.6	4.4±0.6	0.213	0.050
Eggs(g)	30.8±1.2	33.3±1.2	33.3±1.4	31.4±1.5	0.318	0.749
Milk and dairy products(g)	9.4±1.4	9.7±1.4	11.9±1.6	9.2±1.8	0.576	0.840
Fats_oils(g)	40.7±0.9	40.7±0.9	40.4±1.0	40.1±1.1	0.963	0.608

Values shown are mean ± s.e. [a]Adjusted for age, alcohol intake, smoking ,physical activity, per capita annual income, education level, residence(urban/rural) and geographical regions.

[b]Adjusted for age, energy intake, alcohol intake, smoking, physical activity, per capita annual income, education level, residences(urban/rural) and geographical regions.

[c]Statistical significance was determined by analysis of covariance. Compared with Number of MetS components 0 group,*$P < 0.05$, **$P < 0.01$.

For peer review only

Food and nutrient intake in correlation with different MetS components in women

Table 4 shows the multivariate adjusted mean food and nutrient intake, according to the number of MetS components in women. There was a positive correlation between the number of altered MetS and the daily intake of cereals (range of intake: 345.3–351.6g), especially for wheat (101.9–112.6g; *P* trend =0.066). In addition, there was a negative correlation between the number of altered MetS components and the daily intake of milk and dairy products (17.8–11.5g; ANCOVA *P*=0.020, *P* trend=0.002). The daily intake of milk and dairy products for the group 3-5 of altered MetS components was significantly lower than the intake for the group 0 (multiple comparison test *P*=0.009).

Higher daily intake of sodium (3862.3–4005.7mg; ANCOVA *P*=0.001, *P* trend =0.032) was positively correlated with an increased number of altered MetS components, respectively. However, as the daily intake of beta carotene decreased (1578.6–1382.7μg; ANCOVA *P*= 0.001, *P* trend =0.007) the number of MetS components increased. The daily intake of beta carotene in the altered MetS components groups 1 and 3-5 was significantly lower than in group 0 (multiple comparison test *P*=0.001 and *P*=0.003).

For peer review only

For peer review only - http://bmjopen.bmj.com/site/about/guidelines.xhtml

Table 4 Energy and multivariate adjusted[a,b] mean food and nutrient intake according to the number of metabolic syndrome (MetS)components in women (N=3234)

	**Number of MetS components**					
	**0**	**1**	**2**	**3–5**	***P*[c]**	**Trend *P***
n，%	837（25.9%）	906（28.0%）	770（23.8%）	721（22.3%）		
Energy	1972.5±18.8	1966±18.0	1974±19.5	1938.3±20.1	0.549	0.275
Nutrients[a]						
Protein(energy %)	12.7±0.2	12.7±0.2	12.8±0.2	12.8±0.2	0.922	0.567
Fat(energy %)	33.2±0.6	33.4±0.6	33.4±0.7	33.3±0.7	0.979	0.826
Carbohydrate(energy %)	53.9±0.6	53.6±0.6	53.6±0.7	53.7±0.7	0.909	0.715
Nutrients[b]						
Protein(g)	61.0±0.8	61.1±0.8	61.1±0.8	61.4±0.8	0.943	0.583
Beans(protein %)	6.9±0.6	7.2±0.6	7.5±0.6	7.6±0.6	0.449	0.121
Animal foods(protein %)	32.7±1.0	32.3±0.9	32.4±1.0	32.3±1.0	0.929	0.612
Other vegetal food (protein %)	58.6±1.0	59.0±1.0	58.3±1.1	58.5±1.1	0.840	0.695
Fat(g)	72.3±1.4	72.6±1.4	72.5±1.4	72.6±1.5	0.993	0.819
Carbohydrate(g)	259.7±3.2	257.7±3.2	258±3.3	258.5±3.3	0.866	0.689
ß-Carotene(μg)	1578.6±68.0	1391.4±66.9**	1476.5±69.5	1382.7±70.1**	0.001	0.007
Vitamin C(mg)	75.4±2.3	73.9±2.2	76.9±2.3	74.0±2.3	0.292	0.857
Vitamin E(mg)	31.6±0.9	31.9±0.9	32.9±0.9	32.1±0.9	0.309	0.260
Calcium(mg)	353.4±8.4	351.7±8.3	355.5±8.6	342.8±8.7	0.299	0.225
Sodium (mg)	3862.3±116.3	3984.6±114.1	4240.8±118.7**	4005.7±119.0	0.001	0.032
Iron(mg)	19.8±0.5	20.2±0.5	20.2±0.5	19.8±0.5	0.505	0.817
Magnesium(mg)	277.3±4.9	280.9±4.8	278.4±5.0	279.6±5.1	0.804	0.748
Dietary fiber(g)	10.3±0.2	10.6±0.2	10.4±0.2	10.3±0.2	0.555	0.814
Cholesterol(mg)	236.7±10.1	237.6±10.0	243.7±10.3	245.9±10.4	0.640	0.219
Foods[b]						
Cereals(g)	345.3±6.7	347.8±6.5	346.4±6.8	351.6±6.8	0.440	0.335
Rice(g)	216.4±7.3	207.7±7.1	211.1±7.4	205.5±7.5	0.293	0.139
Wheat(g)	101.9±6.4	106.1±6.3	106.1±6.5	112.6±6.5	0.280	0.066
Coarse cereals(g)	16.1±2.1	15.3±2.1	13.5±2.2	15.8±2.2	0.440	0.646
Tubers(g)	39.8±2.9	38.0±2.9	38.8±3.0	35.2±3.0	0.315	0.112
Nuts(g)	3.7±0.6	3.0±0.6	3.8±0.7	3.4±0.7	0.304	0.907
Beans(g)	15.6±1.4	15.7±1.4	16.6±1.4	16.7±1.4	0.691	0.274
Vegetables(g)	302.2±8.3	303.3±8.1	302.6±8.4	298.2±8.5	0.884	0.583
Fruits(g)	77.9±6.3	76.1±6.2	70.5±6.4	73.5±6.5	0.497	0.268
Fish/seafood (g)	37.3±2.9	34.9±2.8	36.8±2.9	37.1±3.0	0.670	0.886
Meats(g)	86.6±3.7	85.7±3.7	81.0±3.8	84.0±3.8	0.275	0.223
Abdominal organs (g)	3.4±0.7	4.5±0.7	4.1±0.8	4.3±0.8	0.284	0.251
Eggs(g)	28.3±1.8	27.2±1.7	28.6±1.8	27.6±1.8	0.714	0.903
Milk and dairy products(g)	17.8±2.5	14.9±2.4	13.1±2.5	11.5±2.5**	0.020	0.002
Fats_oils(g)	34.7±1.2	35.0±1.2	36.0±1.2	36.0±1.2	0.459	0.133

Values shown are mean ± s.e. [a]Adjusted for age, alcohol intake, smoking ,physical activity, per capita annual income, education level, residence(urban/rural) and geographical regions.

[b]Adjusted for age, energy intake, alcohol intake, smoking, physical activity, per capita annual income, education level, residences(urban/rural) and geographical regions.

[c]Statistical significance was determined by analysis of covariance. Compared with Number of MetS components 0 group,**P* < 0.05, ***P* < 0.01.

For peer review only

DISCUSSION

Using data from a partially national representative sample of Chinese adults, we found that higher daily intake of meat, fish/seafood, protein, cholesterol, and lower daily intake of coarse cereals, tubers, and dietary fiber was correlated with the number of altered MetS components in men. We also found that higher daily intake of wheat and sodium, and lower intake of beta carotene, milk, and dairy products was correlated with the number of altered MetS components in women. Rather than using a dietary pattern method of analysis, it might be more accurate to evaluate the effect of the single foods or nutrients on the risk of MetS systematically.[3-6]

Our study found that the increased intake of meat and fish/seafood was positively associated with the number of altered MetS components in the Chinese male population. This correlation is closely related to the rapid evolution of the diet in China, due to the remarkable economic developments and changes in lifestyle since 1980s. During this social revolution, the popular diet pattern shifted from the traditional Chinese food, composed mainly of cereals, vegetables and few animal foods, to the consumption of a western diet (e.g. high energy, high fat, high protein, and low dietary fiber).[31] High energy density in carbohydrate-rich food is evidently linked with high levels of obesity and nutrition-related non-communicable diseases.[32]

Prior findings indicated that meat patterns of Chinese meat consumers were characterized by predominant intake of fatty fresh pork.[33] But the high intake of fats from meat, particularly saturated fat, has been associated with higher plasma lipoprotein levels and higher blood pressure levels.[34] According to the Dietary Reference Intake (DRIs) for Chinese adults, general recommendations propose a total fats intake of 20%–30% of the daily caloric consumption with an emphasis on unsaturated fat; saturated fat should represent <10% of total ingested calories. In opposition to these general recommendations, our study showed that intake of fats (% of energy) was slightly higher than the recommended value in both genders (e.g. 31.7% in men, and 33.2% in women with zero altered MetS components ; 31.9% in men, and 33.3% in women with three or more altered MetS components, respectively). Meanwhile, our previous study observed that adults in China consumed increasingly high cholesterol and deviated from the recommended intake level over the past two decades,[35] but a positive correlation between cholesterol intake and the number of MetS components was only found in men in our study. In contrast, other latest studies have indicated that the effect of dietary cholesterol on incident cardiovascular disease and serum cholesterol outcomes remains unclear.[36 37] Thus, compared with a total fat intake, this observation

For peer review only - http://bmjopen.bmj.com/site/about/guidelines.xhtml

suggests that fats type and cholesterol may be latent factor which affects MetS components risk in men more obvious.

Intake of dietary protein also influences the MetS components, through the percent of protein from various foods, or through quality supplementation of a specific amino acids.[38] A review of epidemiologic evidence indicated that intake of dietary animal proteins appears to increase the risk of MetS.[39] Gadgil MD et al. found that a diet high in animal protein was associated with higher BMI, higher WC, higher total cholesterol, and higher low density lipoprotein cholesterol in South Asians living in the United States.[40] According to the DRIs for Chinese adults, recommendations for daily protein intake are 65g for men and 55g for women.[41] Our study showed that protein intake was also somewhat higher than the recommended amount both in men and women (e.g. 70.4g and 61.0g per day in men and women with zero altered MetS components; 73.4g and 61.4g per day in men and women with three or more altered MetS components, respectively). Interestingly, a positive correlation trend between the intake of dietary protein, the percent of protein ingested from animal food (protein %) and the number of MetS or alterations of its components was only observed in men. This indicated that men and women may vary differently on source and composition of dietary protein and that a animal protein-rich diet in men possibly had important relations to MetS components. [38]

Park et al. reported that elevated serum ferritin levels were independently associated with future development of MetS, during a 5 year follow-up period.[42] Additionally, previous study found that serum ferritin levels were higher in men than in women in Chinese adults, and a positive association between ferritin levels and the prevalence of MetS in both men and women group.[43] Similar to prior findings, after adjusting for potential confounding factors, we found a upward trend between meat intake and an increased number of altered MetS components only in men. This finding indicated that a higher iron intake in men was possibly related to the number of MetS components.

High cereal fiber content and low glycemic index are inherent attributes of most whole-grain foods.[3] Coarse cereals and tubers can provide rich dietary fiber, less fat, and less protein than animal food.[12] Similarly to a study of middle-aged and elderly Japanese people,[6] we found a marginally significant difference between fiber intake in men and the number of altered MetS components. Other data indicated that the protective role of fiber from cereals was higher than that

For peer review only - http://bmjopen.bmj.com/site/about/guidelines.xhtml

from other sources fiber for the development of MetS and type 2 diabetes.[3] Thereby, an inverse association between coarse cereals and tubers and the number of MetS components in our study may be partly explained by dietary fiber. Magnesium is another component of whole grains that may improve insulin sensitivity.[5] However, dietary magnesium intake in both genders was not correlated with the number of altered MetS components in the present study.

Interestingly, our study showed a marginally significant positive correlation between wheat intake and the number of MetS components only in women, after adjusting for potential confounding factors. In China, wheat and rice belong to dietary patterns with different characteristics; wheat tends to be a major component of a modern high-wheat dietary pattern, as mentioned earlier.[7–10] One explanation for this correlation is that the refining wheat process before cooking results in the removal of healthy wheat constituents (e.g. fiber, vitamins, minerals, phytonutrients, and essential fatty acids).[44] Thus, higher carbohydrate consumption (e.g. higher intake of highly refined starch and sugar) could have adverse effects on the profile of metabolic risk[45]. In addition, high glycemic index foods (such as noodles, bread and steamed bread) can induce inflammation, increase oxidant activities, and cause rapid fluctuations of blood glucose and insulin levels.[46] The other explanation for this correlation is that females tend to have a high intake of refined carbohydrates, most of which is prepared by wheat with high levels of salt.[9 10] A stronger positive correlation was observed between sodium intake and the number of altered MetS components in women, which may reflect female dietary habits. High sodium diet not only elevated blood pressure, but also reduced insulin sensitivity.[47]

In keeping with previous studies,[6 48–50] our data showed that the intake of beta carotene, as well as of milk and dairy products was negatively associated with the number of altered MetS components in women. Carotenoids, mainly found in fruit and fresh vegetables, can capture free radicals, quenching singlet oxygen, and improve the protective capability of antioxidants.[51] Milk, rich in calcium and vitamin D, could accelerate the elimination of abdominal fat.[49] More recently, the Chinese Nutrition Society recommended a daily intake of fruits of 200 g to 350 g per capita per day, and a daily intake of milk or its products of 300g per capita per day for the general Chinese population.[52] In contrast to these recommendations, the mean dietary intake of fruits and milk and dairy products was very low for men and women (e.g.54.0 g or 77.9 g of fruit per day in men and women with zero altered MetS components ; 9.4 g and 17.8 g milk and dairy products per day in

For peer review only - http://bmjopen.bmj.com/site/about/guidelines.xhtml

men and women, with zero altered MetS components, respectively). Therefore, our study suggests that increasing the intake of beta carotene, milk and dairy products could possibly have a great potential to prevent MetS or the alteration of its components in the Chinese population, especially among women.

Fruits, vegetables, whole grains, fish, nuts, and dairy products are six kinds of food that may be beneficial for preventing MetS in various dietary patterns, such as the Mediterranean dietary patter, dietary approaches to stop hypertension, and the Nordic diet.[53] It is well known that the Mediterranean dietary patter as important features, represented by the daily intake of whole-grain, fruits, vegetables, and dairy products, as well as the weekly intake of fish, poultry, nuts, and beans. [54] Similar to previous multiethnic reports, our study showed that an downward trend were found between the intake of fruits and vegetables and the number of altered MetS components, but not with statistical significance for either men or women. However, in our study a higher intake of fish/seafood (e.g. fish, shrimp, crab, and shellfish) was positively correlated with an increased number of altered MetS components in men. This inconsistence may be possibly due to the existence of an "alcohol" dietary pattern, characterized by animal foods (e.g. meat, fish/seafood), alcohol, and nuts in the Chinese male population,[12] the fact that in China fish/seafood is preserved with high levels of salt or the fact that the intake of fried dough with soya-bean milk is a typical breakfast for Chinese people[11] .

This study has several limitations. First, there maybe residual confounding factors in this study. For instance, considerable recent progress has been made in the identification of genetic loci that are associated with insulin resistance and MetS[55]; MetS has been related to the increasing prevalence of obesity, which is escalating even among older age groups[56]; Hormone therapy improves the lipid and oxidative alterations that occur in MetS in postmenopausal women[57]. Cumulative alcohol consumption, especially may be an important confounding factor. our previous study found that an "alcohol" dietary pattern was present in Chinese men.[12] In this study the overall prevalence of alcohol consumption is 64.9% in men and 9.2% in women, with three or more altered MetS components. Moderate alcohol intake has consistently been shown to be associated with a lower risk of fatal and non-fatal cardiovascular disease. Wine is an important component of the Mediterranean diet,[44] but heavy drinking was positively associated with MetS and alterations of its components.[58] Due to lack of relevant information, important factors

For peer review only - http://bmjopen.bmj.com/site/about/guidelines.xhtml

mentioned above were excluded from analysis, which may limit the strength of our findings.

Second, the mixed intake of food and nutrients may produce more complex interactive effects than the analysis of the intake of foods and nutrients as evaluation indicators.[14] For instance prior findings have shown that the "Green Water" dietary pattern, which is characterized by high intake of rice, vegetables, and moderate intake of animal foods, has beneficial effects on MetS or on its components.[7–9] But there was not a significant negative correlation between rice and vegetables and the number of altered MetS components in both genders in the present study.

Third, we measured diet using a combination of three consecutive days by 24-hour dietary recall and a household inventory in this study. However, this method has a few shortcomings. One is that the allocation of oil and other condiments among household members was based on the proportion of reference men instead of total food intake, which could lead to an additional level of error for the analysis of individual intake. The other is that dietary data collected by this method omit adjustment for the oil and other condiments comsumption for foods eaten away from home[20]. Additionally, it is unclear whether short-term records of dietary recall can fully reflect the long-term intake of a dietary pattern.[59]

Fourth, despite a progressive increase or decrease in some foods and nutritional factors intake levels (e.g. meat, protein, cholesterol in men and beta carotene, milk, and dairy products in women) was observed according to the cumulative distribution of MetS components, it is uncertain whether this levels would have a direct impact on MetS and their metabolic profiles. Prior correlation findings to the best of our knowledge have not previously been reported. Fifth, because of our cross-sectional design, we could not come to any causal conclusion to explain the relationships between dietary factors and the number of altered MetS components.

CONCLUSIONS

In conclusion, our study showed that some foods and nutritional factors are correlated to the number of altered MetS components among the Chinese adult population. However, whether consumption of these foods and nutritional factors would directly affect metabolic disorders requires further investigation and more prospective and clinical research is needed to examine these associations.

For peer review only - http://bmjopen.bmj.com/site/about/guidelines.xhtml

Contributors MWC, BZ conceived the study. MWC, YFOY and WWD completed all statistical analyses and interpretation of data. MWC drafted the manuscript. ZHW and WWD contributed to the discussion. HJW and BZ revised the manuscript.

Funding The work was supported by the National Institutes of Health (NIH: R01-HD30880, DK056350 and R01-HD38700).

Competing interests None.

Patient consent Obtained.

Ethics approval All protocols, instruments and the process for obtaining informed consent for this study were approved by the Ethical Committee of the University of North Carolina at Chapel Hill and the National Institute for Nutrition and Food Safety of USA, Chinese Center for Disease Control and Prevention. Signed consent forms were obtained from all participants.

Data sharing statement The data used for the analysis are available from the CPC at http://www.cpc.unc.edu/projects/china.

REFERENCES

1. Ahluwalia N, Andreeva VA, Kesse-Guyot E, *et al*. Dietary patterns, inflammation and the metabolic syndrome. *Diabetes Metab* 2013;39:99–110.
2. Xi B, He D, Hu Y, *et al*. Prevalence of metabolic syndrome and its influencing factors among the Chinese adults: The China Health and Nutrition Survey in 2009.*Prev Med* 2013;57: 867–71.
3. McKeown NM, Meigs JB, Liu S, *et al*. Carbohydrate nutrition, insulin resistance, and the prevalence of the metabolic syndrome in the Framingham Offspring Cohort. *Diabetes Care* 2004;27:538–46.
4. Ford ES, Giles WH, Dietz WH.Prevalence of the metabolic syndrome among US adults: findings from the third National Health and Nutrition Examination Survey. *JAMA* 2002;287: 356–59.
5. Evangelopoulos AA,Vallianou NG, panagiotakos DB, *et al*. An inverse relationship between cumulating components of the metabolic syndrome and serum magnesium levels. *Nutr Res* 2008;28: 659–63.
6. Rei Otsuka1,Tomoko Imai1,Yuki Kato1,*et al*. Relationship between number of metabolic syndrome components and dietary factors in middle-aged and elderly Japanese subjects.

For peer review only - http://bmjopen.bmj.com/site/about/guidelines.xhtml

Hypertens Res 2010;33:548-54.

7. Li Y, He Y, Lai J，*et al*. Dietary patterns are associated with stroke in Chinese adults. *J Nutr* 2011;141:1834–39.
8. Wang D, He Y, Li Y，*et al*. Dietary patterns and hypertension among Chinese adults: a nationally representative cross-sectional study. *BMC Public Health* 2011;11:925.
9. He Y, Li Y, Lai. Dietary patterns as compared with physical activity in relation to metabolic syndrome among Chinese adults. *Nutrition, Metabolism & Cardiovascular Diseases* 2013;23:920-28.
10. Batis C, Sotres-Alvarez D, Gordon-Larsen P，*et al*. Longitudinal analysis of dietary patterns in Chinese adults from 1991 to 2009. *Br J Nutr* 2014;111:1441–51.
11. Batis C,Mendez MA, Gordon-Larsen P，*et al*. Using both principal component analysis and reduced rank regression to study dietary patterns and diabetes in Chinese adults. *Public Health Nutr* 2016;19:195-203.
12. Zhang JG. Changes in dietary patterns and their associations with general and central obesity among adults in China (1991-2009).PhD Thesis, Chinese Center for Disease Control and Prevention, 2013.
13. Mishra GD, McNaughton SA,Bramwell GD，*et al*. Longitudinal changes in dietary patterns during adult life.*Br J Nutr* 2006;96:735-44.
14. Hu FB. Dietary pattern analysis: A new direction in nutritional epidemiology. *CurrOpin Lipidol* 2002;13:3–9.
15. Ashima K. Kant. Dietary patterns and health outcomes. J*ournal of the American Dietetic Association* 2004;104:615-35.
16. Ma G, Kong L(editor). The report of 2002 China National Nutrition and Health Survey(Part Nine)—behavior and way of life. Beijing: People's Medical Publishing House,2006.
17. Popkin B.M., DuS, Zhai, F, *et al.* Cohort profile: the China health and nutrition surve-monitoring and understanding socioeconomic and health change in China, 1989–2011. *Int. J. Epidemiol* 2010;39:1435–40.
18. Zhang JG, Wang HJ, Wang YF, *et al.* Ditary patterns and their associations with childhood obesity in China. *British Journal of Nutrition* 2015; 113:1978-84.
19. Zhang JG, Wang ZH, Wang HJ, *et al*. Dietary patterns and their associations with general

For peer review only - http://bmjopen.bmj.com/site/about/guidelines.xhtml

obesity and abdominal obesity among young Chinese women. *European Journal of Clinical Nutrition* 2015; 69:1009-14.

20. Zhai FY, Guo XG, Popkin BM, *et al.* Evalutaion of the 24-hour individual recall method in China. *Food and Nutrition Bulletin* 1996; 17:154–61.
21. Yang Y, Wang G, Pan X (editor). China food composition table. 2nd edition. Beijing: Beijing Medical University Press, 2009.
22. Millen BE, Pencina MJ, Kimokoti RW， *et al*. Nutritional risk and the metabolic syndrome in women: opportunities for preventive intervention from the Framingham Nutrition Study. *Am J Clin Nutr* 2006; 84:434–41.
23. Dami ̃;ao R, Castro TG, Cardoso MA， *et al*. Dietary intakes associated with metabolic syndrome in a cohort of Japanese ancestry. *Br J Nutr* 2006; 96:532–38.
24. Bell AC, Ge K, Popkin BM. Weight gain and its predictors in Chinese adults. *Int J Obes* 2001; 25:1079–86.
25. WHO. Physical Status: The Use and Interpretation of Anthropometry. Report of a WHO Expert Committee. World Health Organization, Geneva, 1995.
26. Chobanian AV, Bakris GL, Black HR， *et al*. The seventh report of the Joint National Committee on prevention, detection, evaluation, and treatment of high blood pressure: the JNC 7 report. *JAMA* 2003;289:2560–72.
27. Batis C,Mendez MA,Sotres-Alvarez D， *et al*. Dietary pattern trajectories during 15 years of follow-up and HbA1c, insulin resistance and diabetes prevalence among Chinese adults. *J Epidemiol Community Health* 2014;68:773–79.
28. Yan S, Li J, Li S， *et al*. The expanding burden of cardiometabolic risk in China: the China Health and Nutrition Survey. *Obes. Rev* 2012;13:810–21.
29. AlbertCi KG, Eckel RH, Grundy SM， *et al*.Harmonizing the metabolic syndrome: a joint interim statement of the international diabetes federation task force on epidemiology and prevention; national heart, lung, and blood institute; American heart association; world heart federation; international atherosclerosis society; and international association for the study of obesity. *Circulation* 2009;20:1640–45.
30. Ainsworth BE, Haskell WL, Whitt MC, *et al*. Compendium of physical activities: an update of activity codes and MET intensities. *Med Sci Sports Exerc* 2000;32:S498-S504.

For peer review only - http://bmjopen.bmj.com/site/about/guidelines.xhtml

31. Du S,Lu B, Zhai F， *et al*. A new stage of the nutrition transition in China. *Public Health Nutr* 2002; 5:169-74.

32. Popkin BM. The nutrition transition: An overview of world patterns of change. *Nutr Rev* 2004;62:S140-S143.

33. Wang ZH, Zhai FY, Wang HJ, *et al*. Secular trends in meat and seafood consumption patterns among Chinese adults, 1991–2011. *European Journal of Clinical Nutrition* 2015; 69: 227–33

34. Woo1 HD, Shin A, Kim J. Dietary patterns of Korean adults and the prevalence of metabolic syndrome: A cross-sectional study. *PLoS One* 2014;9:e111593.

35. Su C, Jia XF, Wang ZH, *et al*. Trends in dietary cholesterol intake among Chinese adults: a longitudinal study from the China Health and Nutrition Survey, 1991–2011. *BMJ Open* 2015; 5:e007532.

36. Samantha B, Gowri R, Rohini V, *et al*. Dietary cholesterol and cardiovascular disease: a systematic review and meta-analysis. *Am J Clin Nutr* 2015;102: 276–94.

37. Fatemeh RK, Gangadaran S, Amy G, *et al*. The role of dietary cholesterol in lipoprotein metabolism and related metabolic abnormalities: a mini-review. *Critical Reviews in Food Science and Nutrition* 2016; 56: 2408–15.

38. Andersen JC, Fernandez ML. Dietary strategies to reduce metabolic syndrome. *Reviews in Endocrine & Metabolic Disorders* 2013; 14,241-54.

39. Amanda B, Terry C, Christine M. Dietary patterns and metabolic syndrome-a review of epidemiologic evidence. *Asia Pac J Clin Nutr* 2006;15:134-42.

40. Gadgil MD, Anderson CA, Kandula NR. Dietary patterns are associated with metabolic risk factors in south Asians living in the United States. *J Nutr* 2015;145:1211-17.

41. Chinese Nutrition Society(editor).Chinese Dietary Reference Intakes(2013 edition).Beijing: Science Press,2014.

42. Park SK, Ryoo JH， *et al*. Association of Serum Ferritin and the Development of Metabolic Syndrome in Middle-Aged Korean Men: A 5-year follow-up study. *Diabetes Care* 2012;35, 2521–26.

43. Li J, Wang R, Luo D. Association between Serum Ferritin Levels and Risk of the Metabolic Syndrome in Chinese Adults: A Population Study. *PLoS One* 2013;8:e74168.

44. Dario G, Antonio C, Katherine E.The effects of diet on inflammation: Emphasis on the

For peer review only

metabolic syndrome. *J Am Coll Cardiol* 2006;48:677–85.

45. Noakes M, Keogh JB, Foster PR，*et al*. Effect of anenergy-restricted, high-protein, low-fat diet relative to a conventional high-carbohydrate, low-fat diet on weightloss, body composition, nutritional status, and markers of cardiovascular health in obese women. *Am J Clin Nutr* 2005;81:1298–306.
46. Avignon A, Hokayem M, Bisbal C， *et al*. Dietary antioxidants: do they have a role to play in the ongoing fight against abnormal glucose metabolism? *Nutrition* 2012;28:715–21.
47. Lastra G, Dhuper S, Johnson MS，*et al*. Salt，aldosterone，and insulin resistance: impact on the cardiovascular system. *Nat Rev Cardiol* 2010;7:577–84.
48. Czernichow S,Vergnaud AC, Galan P， *et al*. Effects of long-term antioxidant supplementation and association of serum antioxidant concentrations with risk of metabolic syndrome in adults. *Am J Clin Nutr* 2009;90:329–35.
49. Sébédio JL, Corinne MB. Metabolic syndrome and dairy product consumption:Where do we stand?. *Food Research International* 2016; 89:1077–84.
50. Kim J. Dairy food consumption is inversely associated with the risk of the metabolic syndrome in Korean adults. *J Hum Nutr Diet* 2013;26,Suppl 1:S171–S179.
51. Sun C, Ling W, Huang G， *et al*(editor).Nutrition and food hygiene.7th edition. Beijing: People's Medical Publishing House, 2013.
52. Chinese Nutrition Society(editor).Chinese Dietary Guide. Beijing: People's Medical Publishing House, 2016.
53. Calton EK, James AP, Pannu PK，*et al*. Certain dietary patterns are beneficial for the metabolic syndrome: reviewing the evidence. *Nutr Res* 2014; 34: 559–68.
54. Schwingshack L, Hoffmann G. Does a Mediterranean-type diet reduce cancer risk? *Curr Nutr Rep* 2016; 5: 9–17.
55. Brown AE, Mark W. Genetics of insulin resistance and the metabolic syndrome. *Curr Cardiol Rep* 2016; 18: doi 10.1007/s11886-016-0755-4.
56. Dominguez LJ, Mario B. The biology of the metabolic syndrome and aging. *Curr Opin Clin Nutr Metab Care* 2016; 19:5-11.
57. Sánchez-Rodríguez MA, Mariano ZF, Castrejón-Delgado L, *et al*. Effects of hormone therapy on oxidative stress in postmenopausal women with metabolic syndrome. *Int. J. Mol. Sci* 2016;

For peer review only - http://bmjopen.bmj.com/site/about/guidelines.xhtml

17: 1388, doi:10.3390/ijms17091388.

58. Im HJ, Park SM, Choi JH, *et al*. Binge drinking and its relation to metabolic syndrome in Korean adult men. *Korean J Fam Med* 2014; 35:173-81.

59. Ranil J. Comparison dietary assessment methods in Sri Lankan adults: use of 24-hour dietary recall and 7-day weighed intake. *BMC Nutrition* 2016; 18: doi 10.1186/s40795-016-0059-5.

For peer review only

For peer review only - http://bmjopen.bmj.com/site/about/guidelines.xhtml

Supplemental Table 1. The full list of food items and examples for each food group using by a household inventory

No	Food group	Examples of food items
1	Rice	Japonica rice, glutinous rice
2	Flour	Steamed bread, noodles
3	Coarse cereals	Corn, barley, millet
4	Tubers	Potato, sweet potato
5	Beans	Beans, mung beans,
6	Vegetables,	Tomato, cabbage, white radish, mungbean sprout
7	Fruits	Apples,watermelons
8	Nuts	Peanuts, melon seeds
9	Pork	
10	Other livestock meat	Cattle, sheep
11	Poultry meat	Chicken, duck, goose
12	Abdominal organs	Pork liver, pork heart
13	Fish/seafood	Fish, shrimp, crab
14	Milk and dairy products	Milk, yogurt
15	Eggs	Eggs, duck eggs
16	Vegetable oil	
17	Fat oil	
18	Snacks and dessert	Bean jelly, cold noodles, cakes
19	Fans/starch and sugar	Starch, sugar
20	Condiments	Salt, soy sauce, sauce

For peer review only - http://bmjopen.bmj.com/site/about/guidelines.xhtml

Supplemental Table 2. Energy and multivariate adjusted[a,b] mean nutrient intake according to the number of metabolic syndrome (MetS)components in men (N=2800)

	Number of MetS components					
	**0**	**1**	**2**	**3−5**	P^b	Trend P
n，%	866（30.9%）	832（29.7%）	618（22.1%）	484（17.3%）		
Energy	2349.9±20.8	2317.5±21.3	2296.7±24.6	2269.6±27.8	0.109	0.016
Nutrients[a]						
VitA(μg RE)	445.8±17.76	443.9±17.58	440.4±19.83	451.1±22.29	0.986	0.886
$VitB_1$(mg)	1.0±0.01	1.0±0.01	1.1±0.02	1.0±0.02	0.688	0.656
$VitB_2$(mg)	0.8±0.01	0.8±0.01	0.8±0.01	0.8±0.01	0.777	0.510
$VitB_3$(mg)	15.1±0.20	15.4±0.19	15.6±0.22	15.6±0.25	0.262	0.101
K(mg)	1750.2±16.31	1740.1±16.15	1727.0±18.25	1753.1±20.47	0.711	0.955
Zn(mg)	11.7±0.10	11.8±0.10	11.9±0.11	12.0±0.13	0.273	0.053
Se(μg)	48.1±0.9	48.2±0.89	48.8±1.00	49.2±1.13	0.858	0.402
P(mg)	1060.9±8.07	1063.2±7.99	1063.4±9.01	1067.7±10.13	0.959	0.596
Mn(mg)	7.0±0.19	7.0±0.19	6.7±0.21	7.3±0.24	0.409	0.701
Cu(mg)	2.1±0.03	2.2±0.03	2.1±0.04	2.2±0.04	0.149	0.141

Values shown are mean ±s.e.

[a]Adjusted for age, energy intake, alcohol intake, smoking, physical activity, per capita annual income, education level, residence(urban/rural) and geographical regions.

[b]Statistical significance was determined by analysis of covariance.

Compared with Number of MetS components 0 group,*$P<0.05$, **$P<0.01$.

For peer review only - http://bmjopen.bmj.com/site/about/guidelines.xhtml

Supplemental Table 3. Energy and multivariate adjusted[a,b] mean nutrient intake according to the number of metabolic syndrome (MetS)components in women (N=3234)

	Number of MetS components					
	0	1	2	3–5	P^b	Trend P
n，%	866（30.9%）	832（29.7%）	618（22.1%）	484（17.3%）		
Energy	2349.9±20.8	2317.5±21.3	2296.7±24.6	2269.6±27.8	0.109	0.016
Nutrients[a]						
VitA(μg RE)	476.2±31.68	452.7±31.12	463.2±32.27	444.9±32.5	0.665	0.333
$VitB_1$(mg)	0.8±0.02	0.9±0.02	0.9±0.02	0.9±0.02	0.412	0.165
$VitB_2$(mg)	0.7±0.02	0.7±0.02	0.7±0.02	0.7±0.02	0.826	0.601
$VitB_3$(mg)	13.4±0.27	13.4±0.26	13.4±0.27	13.5±0.27	0.999	0.868
K(mg)	1605.5±25.61	1603.7±25.20	1608.4±26.14	1582.2±26.31	0.612	0.353
Zn(mg)	10.1±0.15	10.3±0.15	10.2±0.15	10.3±0.15	0.455	0.421
Se(μg)	38.1±0.78	39.2±0.77	38.9±0.80	39.0±0.80	0.312	0.270
P(mg)	900.6±12.84	916.0±12.62	910±13.08	915.1±13.18	0.429	0.284
Mn(mg)	5.5±0.20	5.7±0.19	5.8±0.20	5.6±0.20	0.384	0.575
Cu(mg)	1.8±0.04	1.9±0.04	1.9±0.04	1.9±0.04	0.325	0.132

Values shown are mean ±s.e.

[a]Adjusted for age, energy intake, alcohol intake, smoking, physical activity, per capita annual income, education level, residence(urban/rural) and geographical regions.

[b]Statistical significance was determined by analysis of covariance.

Compared with Number of MetS components 0 group,*$P<0.05$, **$P<0.01$.

For peer review only - http://bmjopen.bmj.com/site/about/guidelines.xhtml

STROBE Statement—checklist of items that should be included in reports of observational studies

	Item No	Recommendation
Title (page 1 line 2-8) Abstract (page2 line 12-55)	1	(*a*) Indicate the study's design with a commonly used term in the title or the abstract
		(*b*) Provide in the abstract an informative and balanced summary of what was done and what was found
**Introduction** (page 3 line 29—page 5 line 4 in Main Document)		
Background/rationale (page 3 line 30—page 4 line 56 in Main Document)	2	Explain the scientific background and rationale for the investigation being reported
Objectives (page 4 line 57—page 5 line 4 in Main Document)	3	State specific objectives, including any prespecified hypotheses
**Methods** (page 5 line 10—page 8 line 40 in Main Document)		
Study design (page 5 line 11—page 5 line 27 in Main Document)	4	Present key elements of study design early in the paper
Setting (page 5 line 11—page 6 line 27 in Main Document)	5	Describe the setting, locations, and relevant dates, including periods of recruitment, exposure, follow-up, and data collection
Participants (page 5 line 29—page 5 line 39 in Main Document)	6	(*a*) *Cohort study*—Give the eligibility criteria, and the sources and methods of selection of participants. Describe methods of follow-up *Case-control study*—Give the eligibility criteria, and the sources and methods of case ascertainment and control selection. Give the rationale for the choice of cases and controls *Cross-sectional study*—Give the eligibility criteria, and the sources and methods of selection of participants
		(*b*) *Cohort study*—For matched studies, give matching criteria and number of exposed and unexposed *Case-control study*—For matched studies, give matching criteria and the number of controls per case
Variables (page 6 line 19—25, page 7 line 8—57 in Main Document)	7	Clearly define all outcomes, exposures, predictors, potential confounders, and effect modifiers. Give diagnostic criteria, if applicable
Data sources/ measurement (page 5 line 44—page 6 line 17, page 6 line 29—page 7 line 1 in Main Document)	8*	For each variable of interest, give sources of data and details of methods of assessment (measurement). Describe comparability of assessment methods if there is more than one group
Bias (page 7 line 28—57 in Main Document)	9	Describe any efforts to address potential sources of bias
Study size (page 5 line 18—20 in Main Document)	10	Explain how the study size was arrived at
Quantitative variables	11	Explain how quantitative variables were handled in the

For peer review only - http://bmjopen.bmj.com/site/about/guidelines.xhtml

(page 7 line 42—57, page 8 line 30—36 in Main Document)		analyses. If applicable, describe which groupings were chosen and why
Statistical methods<br>(page 8 line 16—25, page 8 line 33—36in Main Document)	12	(*a*) Describe all statistical methods, including those used to control for confounding
		(*b*) Describe any methods used to examine subgroups and interactions
		(*c*) Explain how missing data were addressed
		(*d*) *Cohort study*—If applicable, explain how loss to follow-up was addressed<br>*Case-control study*—If applicable, explain how matching of cases and controls was addressed<br>*Cross-sectional study*—If applicable, describe analytical methods taking account of sampling strategy
		(*e*) Describe any sensitivity analyses

Continued on next page

For peer review only

**Results**(page 8 line 45—page 15 line 59 in Main Document)		
Participants (This is observational studies)	13*	(a) Report numbers of individuals at each stage of study—eg numbers potentially eligible, examined for eligibility, confirmed eligible, included in the study, completing follow-up, and analysed
		(b) Give reasons for non-participation at each stage
		(c) Consider use of a flow diagram
Descriptive data (This is observational studies)	14*	(a) Give characteristics of study participants (eg demographic, clinical, social) and information on exposures and potential confounders
		(b) Indicate number of participants with missing data for each variable of interest
		(c) *Cohort study*—Summarise follow-up time (eg, average and total amount)
Outcome data (This is observational studies)	15*	*Cohort study*—Report numbers of outcome events or summary measures over time
		Case-control study—Report numbers in each exposure category, or summary measures of exposure
		Cross-sectional study—Report numbers of outcome events or summary measures
Main results (page 8 line 45—page 15 line 59 in Main Document)	16	(*a*) Give unadjusted estimates and, if applicable, confounder-adjusted estimates and their precision (eg, 95% confidence interval). Make clear which confounders were adjusted for and why they were included
		(*b*) Report category boundaries when continuous variables were categorized
		(*c*) If relevant, consider translating estimates of relative risk into absolute risk for a meaningful time period
Other analyses (None)	17	Report other analyses done—eg analyses of subgroups and interactions, and sensitivity analyses
**Discussion**(page 16 line 2—page 20 line 55 in Main Document)		
Key results (page 16 line 3—15，page 20 line 47—54 in Main Document)	18	Summarise key results with reference to study objectives
Limitations (page 19 line 36—page 20 line 42)	19	Discuss limitations of the study, taking into account sources of potential bias or imprecision. Discuss both direction and magnitude of any potential bias
Interpretation (page 16 line 17—page 19 line 34 in Main Document)	20	Give a cautious overall interpretation of results considering objectives, limitations, multiplicity of analyses, results from similar studies, and other relevant evidence
Generalisability (page 20 line 31—42 in Main Document)	21	Discuss the generalisability (external validity) of the study results
**Other information**(page 21 line 2—23 in Main Document)		
Funding (page 21 line 7—8 in Main Document)	22	Give the source of funding and the role of the funders for the present study and, if applicable, for the original study on which the present article is based

*Give information separately for cases and controls in case-control studies and, if applicable, for exposed and unexposed groups in cohort and cross-sectional studies.

For peer review only

Note: An Explanation and Elaboration article discusses each checklist item and gives methodological background and published examples of transparent reporting. The STROBE checklist is best used in conjunction with this article (freely available on the Web sites of PLoS Medicine at http://www.plosmedicine.org/, Annals of Internal Medicine at http://www.annals.org/, and Epidemiology at http://www.epidem.com/). Information on the STROBE Initiative is available at www.strobe-statement.org.

For peer review only

For peer review only - http://bmjopen.bmj.com/site/about/guidelines.xhtml

BMJ Open

Relationship between dietary factors and the number of altered metabolic syndrome components in Chinese adults: a cross-sectional study using data from the China Health and Nutrition Survey

Journal:	*BMJ Open*
Manuscript ID	bmjopen-2016-014911.R2
Article Type:	Research
Date Submitted by the Author:	03-Mar-2017
Complete List of Authors:	Cheng, Maowei; National Institute for Nutrition and Health, Chinese Center for Disease Control and Prevention; Center for Disease Control and Prevention of Hubei Province<br>Wang, Huijun ; National Institute for Nutrition and Health, Chinese Center for Disease Control and Prevention<br>Wang, Zhihong ; National Institute for Nutrition and Health, Chinese Center for Disease Control and Prevention<br>Du, Wenwen ; National Institute for Nutrition and Health, Chinese Center for Disease Control and Prevention<br>Ouyang, Yifei ; National Institute for Nutrition and Health, Chinese Center for Disease Control and Prevention<br>Zhang, Bing; National Institute for Nutrition and Health, Chinese Center for Disease Control and Prevention
**Primary Subject Heading**:	Public health
Secondary Subject Heading:	Nutrition and metabolism
Keywords:	Adults, Chinese, Components, Dietary factors, Metabolic syndrome

SCHOLARONE™ Manuscripts

For peer review only

Relationship between dietary factors and the number of altered metabolic syndrome components in Chinese adults: a cross-sectional study using data from the China Health and Nutrition Survey

Maowei Cheng[1,2], Huijun Wang[1], Zhihong Wang[1], Wenwen Du[1], Yifei Ouyang[1],and Bing Zhang[1,*]

[1]National Institute for Nutrition and Health, Chinese Center for Disease Control and Prevention, Beijing, China

[2] Center for Disease Control and Prevention of Hubei Province, Wuhan, China

[*] **Corresponding author:** Bing Zhang

E-mail:zzhangb327@aliyun.com

Postal address:No.27 Nan Wei Rd., Xi Cheng District, Beijing, zip code:100050

Telephone: 010-83151561(office), 18601109679(Mobile phone)

Fax numbers: 010-67711813

Keywords: Adults; Chinese; Components; Dietary factors; Metabolic syndrome

Word count: 4738 words

For peer review only - http://bmjopen.bmj.com/site/about/guidelines.xhtml

Abstract

Objectives: This study aims to study the correlation between dietary factors and the number of altered metabolic syndrome components (MetS) in Chinese adults systematically.

Setting: A cross-sectional study using demographic and dietary data of adults aged 18–75 years from the China Health and Nutrition Survey (2009) was conducted in nine provinces in China.

Participants: There were 6,034 eligible subjects (2,800 men and 3,234 women) in this study.

Outcomes: The primary outcome of this study were diet assessments and the number of altered MetS components. Dietary intake was measured using a combination of a 3-day period with 24-hour and household food inventory; average daily intakes of nutrients were estimated according to the Chinese Food Composition Table. Blood samples were analyzed in a national central lab and the number of clustering MetS components was calculated by adding the presence of each MetS component.

Results: After adjusting for covariates, and taking zero MetS as comparison, the high risk factors correlating with increased numbers of altered MetS components in men were higher intake of protein (70.4–73.4g;*P* trend=0.0004), cholesterol (238.7–266.6mg;*P* trend=0.004), meat (90.6–105.7g;*P* trend=0.016),fish/seafood (30.4–42.3g;*P* trend=0.001), and lower intake of coarse cereals (16.5–12.7g; *P* trend= 0.051), tubers (37.3–32.7g; *P* trend= 0.030), and dietary fiber (11.7–11.5 g; ANCOVA *P*= 0.058). Meanwhile, the high risk factors correlating with increased number of altered MetS components in women were higher intake of wheat (101.9–112.6g; *P* trend =0.066) and sodium (3862.3–4005.7mg, *P* trend =0.032); , and lower intake of ß-carotene (1578.6–1382.7μg; *P* trend =0.007), milk, and dairy products (17.8–11.5g; *P* trend=0.002).

Conclusions: Some foods and nutritional factors correlate with an increased number of altered MetS components in Chinese adults. More prospective, multicenter and clinical research work to further examine these associations is underway.

For peer review only - http://bmjopen.bmj.com/site/about/guidelines.xhtml

Strengths and limitations of this study

1. This is the first observational study to examine the relationship between dietary factors and the number of altered MetS components among Chinese adults in a systematic way.
2. This study includes a large sample size that is fairly representative of the Chinese population. Furthermore, only healthy adults that were not using chronic medication were included.
3. In this study, we measured diet using a combination of three consecutive days by 24-hour dietary recall and a household inventory. One of the prominent advantages of this method is that it combines 24-hour individual recall with edible oil and other condiments consumption measured by household inventory, which greatly improve the quality of individual dietary data.
4. The major limitation of this study is our inability to come to any causal conclusion due to the cross-sectional design.

INTRODUCTION

Metabolic syndrome (MetS) is a cluster of risk factors (dysglycemia, elevated blood pressure, dyslipidaemia, and central adiposity) that has been shown to be predictive of cardiovascular disease and diabetes.[1]

The overall age-standardized estimates of MetS prevalence in Chinese adults was 18.2%, according to the International Diabetes Federation, and 21.3%, according to the modified Adult Treatment Panel III criteria of the National Cholesterol Education Program (NCEP-ATP III). These estimates of MetS prevalence increase gradually with age.[2] Therefore, MetS is considered as a major challenge that affects the quality of life of millions of people. It is urgent to identify effective strategies to better control MetS.

MetS requires 3 of 5 components: waist circumference (WC), fasting glucose, high density lipoprotein cholesterol (HDL-C), triglycerides, and hypertension[1].MetS in individual patients, may involve the alteration of various combinations of these components. Accumulating evidence has suggested that an increased number of altered MetS components is strongly associated with a higher risk of developing diseases.[3–6] Serum magnesium level decreased as the number of altered MetS components increased in elderly Greek patients[5]. The number of altered MetS components in middle-aged and elderly Japanese patients was related to lower intake of vitamin B_6,[6] and lower dietary fiber in men, and to lower intake of calcium, milk, dairy products, and higher cereal intake in

For peer review only - http://bmjopen.bmj.com/site/about/guidelines.xhtml

women, after adjusting for age, energy intake, alcohol intake, smoking status, physical activity.

An increasing number of studies in China have focused on the influence of dietary patterns on the prevalence of MetS and its altered components. Using data from the 2002 China National Nutrition and Health Survey (CNNHS), three types of dietary patterns have been identified in the Chinese population. One dietary pattern is called “Yellow Earth” or “traditional northern” pattern; it is characterized by consumption of refined cereal products, salted vegetables and tubers. Another dietary pattern is called “Western/new affluence”; it is characterized by consumption of beef, lamb, milk, cheese, and yogurt. The third dietary pattern is called “Green Water” or “traditional southern” pattern; it is characterized by consumption of rice, vegetables, and moderate amounts of animal foods.[7–9] The “Yellow Earth” pattern and the “Western/new affluence” pattern were associated with an increased likelihood of MetS, or with increased alterations of its components,[8 9]in comparison to the “Green Water” dietary pattern. Our previous studies have identified a modern high-wheat pattern that is characterized by consumption of wheat products, nuts, fruits, eggs, milk and instant noodles or frozen dumplings. This modern pattern could be considered as a combination of the “Yellow Earth” and “Western/new affluence” patterns, and has been positively associated with diabetes.[10 11]Additionally, in our previous studies an “alcohol” dietary pattern was identified among men. This dietary pattern is characterized by consumption of meat, alcohol, and nuts, and has been positively associated with overweight/obesity and central obesity.[12]

However, it would be difficult to obtain an identical relationship between dietary patterns (or dietary index) and the relevant disease outcomes in different studies[13].More importantly, because diet is a complex variable, it is necessary to adopt multiple approaches to examine the relationship between diet and the risk of diseases. If there were any effects caused by a particular nutrient, the dietary pattern approach would not be optimal to examine the relationship between diet and risk of diseases because the effect of that nutrient would be diluted by other factors.[14]

There are significant differences in the dietary characteristics of men and women.[15 16] To our knowledge, the nutrients that could have a positive effect on MetS or on its components have not been determined in men and women, and no studies have explored the association between dietary factors and the number of altered MetS components in the Chinese population. The aim of our study was to examine the relationship between dietary factors and the number of altered MetS components in a systematic way, using a large sample of Chinese adults (N = 6034).

For peer review only - http://bmjopen.bmj.com/site/about/guidelines.xhtml

METHODS

Study population

We used the data collected by the China Health and Nutrition Survey (CHNS) 2009.The CHNS is an ongoing longitudinal survey designed to examine the association between economic and social changes and a range of health behaviors across space and time.[10] A multistage, random cluster sampling method was used to select the samples from nine provinces (Liaoning, Heilongjiang, Jiangsu, Shandong, Henan, Hubei, Hunan, Guangxi and Guizhou) in China. Questionnaire information was used to collect the data as follows: social and economic factors, health factors, nutrition factors and population factors of sociology. However, blood samples were collected only for the first time in 2009. More details about the CHNS data have been described elsewhere.[17]

In 2009, there were a total of 7,755 respondents (3,589 men and 4,166 women) age ≥18 years. Individuals with incomplete dietary data (n=296), individuals over 75 years of age (n=419), individuals suffering from chronic diseases (hypertension, diabetes, stroke, myocardial infarction), individuals with a change in diet or physical and/or pharmacological treatment (n=906), and women in pregnancy or lactation status (n=100) were excluded. Finally, a total of 6,034 adults (2,800 men and 3,234 women) were included in our analysis.

Diet assessments

The 2009 CHNS combined three consecutive days of a 24-hour dietary recall and a household inventory to assess individual consumption. Individual dietary intake for three consecutive days (two week days and one weekend day) was collected for every household member. Food items consumed at restaurants, canteens and other locations away from home were systematically recorded. Using food models and pictures, trained field interviewers recorded the amounts of all foods and beverage items (measured in grams) consumed during 24 h in the previous day[18 19]. In addition, household food intake was determined on a daily basis by calculating the changes in food inventory. Chinese balance with a maximum limit of 15 kg and a minimum limit of 20 g was used to measure household consumption by inventory changes, from the beginning to the end of each day. At the same time, all foods and condiments in the home inventory (purchased from markets, or picked from gardens, and also food waste) were carefully recorded at the start and end of each survey[20].The full list of food groups used in the household inventory have been described in supplemental Table 1 and elsewhere.[11]

For peer review only - http://bmjopen.bmj.com/site/about/guidelines.xhtml

The individual daily intake value for each food item was assessed using data from the 24-hour dietary recall, which was enhanced by use of data from household measures. Additionally, edible oils, and other common condiments (sugar, starch soya sauce, salt) consumed in the household by each member, were allocated based on the proportion of reference men. The amount of nutrients for each food is available from the Chinese Food Composition Table (2nd edition)[21]. Per capita daily nutrients were calculated by combining both of these.

Twelve nutrients, 13 foods, the percentage of energy from protein and fat, and the percentage of protein from various foods were selected as diet indicators according to previous studies[6 22 23].

Other measurements

Weight, height and waist circumference were measured by trained surveyors using standard measurement techniques: Height was measured without shoes to the nearest 0.2cm using a portable stadiometer. Weight was measured without shoes and in light clothing to the nearest 0.1kg on a calibrated beam scale.[24] Between the lowest rib and the iliac crest in a horizontal plane, waist circumference was measured at a point midway using non-elastictape.[25] According to a standard protocol, blood pressure was measured by trained surveyors using a mercury sphygmomanometer.[26]

Blood samples were collected by vein puncture after an overnight fast. All samples were analyzed in a national central lab in Beijing (medical laboratory accreditation certificate ISO 15189:2007) under strict quality control. Fasting plasma glucose was measured with a glucose oxidase phenol 4-aminoantipyrine peroxidase kit (Randox, Crumlin, UK) in a Hitachi 7600 automated analyzer (Hitachi Inc., Tokyo, Japan).[11 27] High-density lipoprotein cholesterol (HDL-C) and triglycerides (TG) were both measured by their corresponding reagents (Kyowa MedexCo., Ltd., Tokyo, Japan) using the glycerol-phosphate oxidase method, and the polyethylene glycol modified enzyme method, respectively. All lipid measurements were carried out on the Hitachi 7600 automated analyzer (Hitachi Inc., Tokyo, Japan).[28] History of chronic patients (past/present), smoking drinking (y/n) and the occupational physical activity were collected using questionnaires.

Definition of MetS and its components

The definition of MetS was based on the most recent Joint Interim Statement (JIS) criteria.[29] As mentioned earlier, subjects under treatment for chronic diseases (hypertension, diabetes, stroke, myocardial infarction) were excluded in present study. Thus, MetS was defined as the presence of three or more of the following five components: (1)abdominal obesity, WC men≥90cm and

For peer review only - http://bmjopen.bmj.com/site/about/guidelines.xhtml

women≥80cm,specifically for Asia adults;[6] (2)elevated blood pressure, SBP/DBP≥130/85mmHg; (3)hypertriglyceridemia,triglyceride≥1.70mmoll^{-1};(4)low high-density lipoprotein cholesterol (HDL-C), HDL-C<1.0 mmoll^{-1} in men and<1.3 mmoll^{-1} in women; and (5) elevated blood glucose levels, fasting blood glucose≥5.6 mmoll^{-1}.The number of clustering MetS components was calculated by adding the number of MetS components.[6]

Definition of covariates

According to the definition of WHO, adults who smoked at least one cigarette a day are defined as current smokers.[12] To determine alcohol consumption, individuals were asked the question: "Have you consumed alcohol (beer, wine or other alcoholic beverage) during the past year (yes, no)?".[2] Residence was divided into urban and rural and geographical region was divided into the North (Liaoning, Heilongjiang, Henan and Shandong) and the South (Hunan, Hubei, Jiangsu, Guangxi and Guizhou). [12]Body mass index (BMI) was calculated as weight in kilograms divided by the square of height in meters.

Current economic status was assessed by mean per capita annual income (unit: RMB Yuan) in the year before the 2009 CHNS. The subjects were classified as low, moderate, and high income level by trisected percentiles of the mean per capita annual income. Participants were interviewed using a semi-quantitative assessment to determine their occupational, domestic, travel, and leisure physical activity levels. The intensity (metabolic equivalent, METs, unit kcal/kg h) of each activity in the questionnaire was coded according to the compendium of physical activities.[30] The total metabolic equivalent (MET-hours/week) was a combined score calculated by multiplying the frequency, duration, and intensity of physical activity. Total metabolic equivalent scores were categorized into three levels (mild, moderate, and high) by trisected percentiles for further analysis.

Statistical analysis

Statistical Analysis System 9.2 (SAS Institute, Cary, NC, USA) was used for all statistical analyses. Subjects were categorized into four groups according to the number of clustering MetS components (0, 1, 2, 3–5).Values of three to five clustering MetS components were combined because only a few subjects had alterations in four or five MetS components (four altered components: 121 men and 215 women; five altered components: 28 men and 64 women). Associations between categorical variables were tested using a chi-Square Test. Comparisons

For peer review only - http://bmjopen.bmj.com/site/about/guidelines.xhtml

For peer review only

between continuous variables were performed by analysis of variance, and 95% confidence intervals (CI) were estimated.

Assessment of model effects and trend tests were performed to analyze the association between each of the dietary indicators and the number of altered MetS components using a generalized linear regression model. Adjustments were made for all potential confounding factors, including age, energy intake, alcohol intake, smoking status, per capita annual income, education levels, physical activity levels, residence (urban/rural) and geographical region (North/South). Mean nutritional intakes were calculated by the number of MetS components (0, 1, 2, 3–5).The estimated marginal means for each of the dietary indicators of altered MetS componentsgroups1, 2, and 3–5 were compared with those of MetS components group 0 by multiple comparison test adjusted using the Bonferroni correction.

All reported *P*-values were estimated using two-sided analysis. A *P*-value <0.05 was considered statistically significant.

RESULTS

Characteristics of the study population

Table 1 shows the characteristics and distribution of all the subjects included in the study categorized by sex. Mean age ± s.d. was 47.3 ± 13.6 years for men, and 47.6 ± 13.1 years for women. Body mass index (BMI) was 23.0 ± 3.1 kgm^{-2} for men, and 23.0 ± 3.2 kgm^{-2} for women. The number of altered MetS components in both men and women gradually increased as age and BMI increased.

We estimated that 58.0% of men currently smoked, and 63.0% consumed alcohol; 3.2% of women smoked, and 9.2% consumed alcohol.

Overall, there was a positive association between the number of altered MetS components and the proportion of moderate and high per capita annual income, but only in men. In women, the number of altered MetS components was associated with a higher proportion of individuals with an educational level of primary school and under. In addition, there was a negative relationship between the number of altered MetS components in men and women and moderate and high physical activity levels.

Prevalence of MetS and metabolic abnormalities

For peer review only - http://bmjopen.bmj.com/site/about/guidelines.xhtml

As shown in Table 2, the overall prevalence of MetS was significantly higher in women (22.3%, 95% CI 20.9, 23.7) than in men (17.3%, 95% CI 15.9, 18.7). Significant differences between sexes were noted in the analysis of individual components. Elevated blood pressure, hypertriglyceridemia, and hyperglycemia were more frequent in men than in women. Abdominal obesity and low HDL cholesterol were more frequent in women than in men. In men, hypertension was the most prevalent component of MetS (38.6%), followed by hypertriglyceridemia (31.5%). In women, the most prevalent MetS component was abdominal obesity (49.4%), followed by low HDL-C (31.0%).

Food and nutrient intake in correlation with different MetS components in men

Table 3 shows multivariate adjusted mean food and nutrients intake, according to the number of altered MetS components in men. Higher daily intake of meat (range of intake: 90.6–105.7g) and fish/seafood (30.4–42.3g) was positively correlated with an increased number of altered MetS components(*P* trend=0.016 for meat; *P* trend=0.001 for fish/seafood).Although analysis of covariance or trend tests did not reach statistical significance, the number of altered MetS components were positively correlated with daily intakes of abdominal organs (2.9–4.4g; *P* trend = 0.050), beans (17.0–19.5g; *P* trend=0.113), and nuts (3.2–4.1g;*P* trend =0.207).

Among the food groups, there was a negative correlation between the number of altered MetS components and the daily intake of cereals (453.9– 440.8g; ANCOVA *P* = 0.083, *P* trend= 0.058), coarse cereals (16.5–12.7g; *P* trend = 0.051), and tubers (37.3–32.7g; ANCOVA *P* = 0.048, *P* trend = 0.030).

An increased number of altered MetS components were positively correlated with a higher daily intake of protein (70.4–73.4g; *P* trend=0.0004) and cholesterol (238.7–266.6mg;*P* trend =0.004). It is worth noting that there was a positive correlation between an increased number of altered MetS components and an increased energy daily intake of protein (12.3%–12.8%; ANCOVA *P*=0.001, *P* trend=0.0001),and with increased daily intake of protein of animal origin (28.6%–30.4%; ANCOVA *P*=0.021, *P* trend=0.020).

Among the dietary indicators, there was a marginally negative difference between the number of altered MetS components and the intake of dietary fiber (11.7–11.5g; ANCOVA *P*= 0.058). Dietary cholesterol intake in the groups of MetS components 1, 2 and 3–5 were significantly higher than in the group 0 (ANCOVA *P*=0.002; multiple comparison test *P*=0.006, *P*=0.003, *P*=0.012).

For peer review only - http://bmjopen.bmj.com/site/about/guidelines.xhtml

Food and nutrient intakein correlation with different MetS components in women

Table 4 shows the multivariate adjusted mean food and nutrient intake, according to the number of MetS components in women. There was a positive correlation between the number of altered MetS and the daily intake of cereals (range of intake: 345.3–351.6g), especially for wheat (101.9–112.6g; *P* trend =0.066). In addition, there was a negative correlation between the number of altered MetS components and the daily intake of milk and dairy products (17.8–11.5g; ANCOVA *P*=0.020, *P* trend=0.002).The daily intake of milk and dairy products for the group 3–5 of altered MetS components was significantly lower than the intake for the group 0 (multiple comparison test *P*=0.009).

Higher daily intake of sodium (3862.3–4005.7mg; ANCOVA *P*=0.001,*P* trend=0.032) was positively correlated with an increased number of altered MetS components, respectively. However, as the daily intake of beta carotene decreased (1578.6–1382.7μg; ANCOVA *P*= 0.001, *P* trend =0.007) the number of MetS components increased. The daily intake of beta carotene in the altered MetS components groups 1 and 3–5 was significantly lower than in group 0 (multiple comparison test *P*=0.001 and *P*=0.003).

For peer review only

For peer review only - http://bmjopen.bmj.com/site/about/guidelines.xhtml

Table 1 Subject characteristics according to the number of metabolic Syndrome (MetS) components

		Number of MetS components					
	**ALL**	**0**	**1**	**2**	**3-5**	P^a	**Trend *P***
Men							
n, %	2800 (100.0%)	866 (30.9%)	832 (29.7%)	618 (22.1%)	484 (17.3%)		
Age (year)	47.3±13.6	44.0±14.1	48.0±13.7	48.7±12.8	50.2±12.0	<0.001	<0.001
Body mass index (Kgm^{-2})	23.0±3.1	21.2±2.4	22.5±2.8	24.0±2.8	25.7±2.9	<0.001	<0.001
Energy intake (Kcal per day)	2308.4±610.4	2349.9±614.1	2317.5±607.0	2296.7±601.3	2269.6±27.8	0.109	0.016
Urban residents(n,%)	740(26.4)	181(20.9)	204(24.5)	183(29.6)	172(35.5)	<0.001	<0.001
Northern residents(n,%)	1176(42.0)	300(34.6)	342(41.1)	292(47.2)	242(50.0)	<0.001	<0.001
Moderate and high physical activity(n,%)	1674 (59.7%)	556(19.9)	516(18.4)	349(12.5)	253(9.0)	<0.001	<0.001
Moderate and high per capita annual income(n,%)	1864 (67.1%)	564(20.3)	532(19.2)	424(15.3)	344(12.4)	0.005	0.001
Education level(n,%)							
Primary school and under	876 (31.3)	253(9.0)	283(10.1)	197(7.0)	143(5.1)		
Junior middle school	1122 (40.1)	369(13.2)	333(11.9)	225(8.0)	195(7.0)	0.062	0.438
High school and above	798 (28.5)	243(8.7)	214(7.7)	195(7.0)	146(5.2)		
Drinker(n,%)	1764(63.0%)	525(60.6%)	523(62.9%)	402(65.0%)	314(64.9%)	0.267	0.061
Current smoker (n,%)	1623(58.0%)	516(59.6%)	496(59.6%)	341(55.2%)	270(55.8%)	0.189	0.062
Women							
n, %	3234 (100.0%)	837 (25.9%)	906 (28.0%)	770 (23.8%)	721 (22.3%)		
Age (year)	47.6±13.1	41.2±12.6	46.4±13.1	50.2±12.0	53.7±11.1	<0.001	<0.001
Body mass index (Kgm^{-2})	23.0±3.2	20.8±2.1	22.3±2.9	24.0±2.9	25.3±3.0	<0.001	<0.001
Energy intake (Kcal per day)	1963.4±535.8	1972.5±522.0	1966±532.7	1974±552.5	1938.3±537.4	0.549	0.275
Urban residents (n,%)	948(29.3)	233(27.8)	267(29.5)	208(27.0)	240(33.3)	0.039	0.068
Northern residents (n,%)	1369(42.3)	279(33.3)	388(42.8)	349(45.3)	353(49.0)	<0.001	<0.001
Moderate and high physical activity (n,%)	1921(59.4%)	553(17.1)	552(17.1)	453(14.0)	363(11.2)	<0.001	<0.001

For peer review only - http://bmjopen.bmj.com/site/about/guidelines.xhtml

Moderate and high per capita annual income(n,%)	2119 (66.4%)	574(18.0)	581(18.2)	487(15.3)	477(14.9)	0.032	0.616
Education level(n,%)							
Primary school and under	1508 (46.7%)	280(8.7)	413(12.8)	406(12.6)	409(12.7)		
Junior middle school	1003 (31.0%)	297(9.2)	306(9.5)	213(6.6)	187(5.8)	＜0.001	＜0.001
High school and above	720 (22.3%)	259(8.0)	187(5.8)	150(4.6)	124(3.8)		
Drinker(n,%)	299(9.2%)	90(10.8%)	79(8.7%)	64(8.3%)	66(9.2%)	0.338	0.243
Current smoker (n,%)	105(3.2%)	18(2.2%)	28(3.1%)	26(3.4%)	33(4.6%)	0.061	0.008

Values shown are mean±s.d.

[a]Statistical significance was determined by analysis of variance or a chi-Square test.

For peer review only - http://bmjopen.bmj.com/site/about/guidelines.xhtml

Table 2 Prevalence of metabolic abnormalities according to the number of metabolic Syndrome (MetS) components

	ALL		Number of MetS components							
			0		1		2		3–5	
	**n,%**	**95% CI**	**n,%**	**95% CI**	**n,%**	**95% CI**	**n,%**	**95% CI**	**n,%**	**95% CI**
Men	2800(100.0)	15.9,18.7	866 (30.9)	27.8,34.0	832 (29.7)	28.0,31.4	618 (22.1)	20.6,23.6	484 (17.3)	15.9,18.7
Metabolic abnormalities (n,%)										
Waist circumference (≥90cm)	710(25.4)	23.8,27.0	0(-)	—	111(13.3)	11.0,15.6	254(41.1)	37.2,45.0	345(71.3)	67.3,75.3
Triglyceride (≥1.7(mmoll^{-1})	883(31.5)	29.8,33.2	0(-)	—	187(22.5)	19.7,25.3	296(47.9)	44.0,51.8	400(82.6)	79.2,86.0
HDL-cholesterol (<1.0mmoll^{-1})	411(14.7)	13.4,16.0	0(-)	—	56(6.7)	5.0,8.4	119(19.3)	16.2,22.4	236(48.8)	44.3,53.3
Blood pressure (≥130/85mmHg)	1081(38.6)	36.8,40.4	0(-)	—	368(44.2)	40.8,47.6	354(57.3)	53.4,61.2	359(74.2)	70.3,78.1
Fasting glucose (≥5.6mmoll^{-1})	612(21.9)	20.4,23.4	0(-)	—	110(13.2)	10.9,15.5	213(34.5)	30.8,38.2	289(59.7)	55.3,64.1
Women	3234(100.0)	20.9,23.7	837 (25.9)	22.9,28.9	906 (28.0)	26.5,29.5	770 (23.8)	22.3,25.3	721 (22.3)	20.9,23.7
Metabolic abnormalities (n,%)										
Waist circumference(≥80cm)	1599(49.4)	47.7,51.1	0(-)	—	396(43.7)	40.5,46.9	555(72.1)	68.9,75.3	648(89.9)	87.7,92.1
Triglyceride (≥≥1.7mmoll^{-1})	793(24.5)	23.0,26.0	0(-)	—	78(8.6)	6.8,10.4	209(27.1)	24.0,30.2	506(70.2)	66.9,73.5
HDL-cholesterol(<1.3mmoll^{-1})	1002(31.0)	29.4,32.6	0(-)	—	191(21.1)	18.4,23.8	297(38.6)	35.2,42.0	514(71.3)	68.0,74.6
Blood pressure (≥130/85mmHg	964(29.8)	28.2,31.4	0(-)	—	162(17.9)	15.4,20.4	324(42.1)	38.6,45.6	478(66.3)	62.8,69.8
Fasting glucose (≥5.6mmoll^{-1})	594(18.4)	17.1,19.7	0(-)	—	79(8.7)	6.9,10.5	155(20.1)	17.3,22.9	360(49.9)	46.3,53.5

For peer review only

For peer review only - http://bmjopen.bmj.com/site/about/guidelines.xhtml

Table 3 Energy and multivariate adjusted[a,b] mean food and nutrient intake according to the number of metabolic syndrome (MetS)components in men (N=2800)

	Number of MetS components					
	0	1	2	3–5	*P*[c]	Trend *P*
n,%	866 (30.9%)	832 (29.7%)	618 (22.1%)	484 (17.3%)		
Energy	2349.9±20.8	2317.5±21.3	2296.7±24.6	2269.6±27.8	0.109	0.016
Nutrients[a]						
Protein(energy %)	12.3±0.1	12.6±0.1	12.6±0.1*	12.8±0.1**	0.001	0.0001
Fat(energy %)	31.7±0.4	31.6±0.4	32.1±0.4	31.9±0.5	0.748	0.558
Carbohydrate(energy %)	55.8±0.4	55.7±0.4	55.1±0.4	55.0±0.5	0.460	0.141
Nutrients[b]						
Protein(g)	70.4±0.6	71.5±0.6	72.1±0.6	73.4±0.7**	0.006	0.0004
Beans(protein %)	6.6±0.3	6.2±0.3	6.4±0.3	6.5±0.4	0.849	0.935
Animal foods(protein %)	28.6±0.6	29.9±0.6	31.0±0.6**	30.4±0.7	0.021	0.020
Other vegetal food (protein %)	62.9±0.6	61.1±0.6	60.3±0.7*	60.5±0.8*	0.011	0.007
Fat(g)	80.8±1.0	80.8±1.0	82.1±1.1	81.2±1.2	0.790	0.595
Carbohydrate(g)	321.0±2.3	319.1±2.3	314.7±2.6	317.7±2.9	0.263	0.192
ß-Carotene(µg)	1493.6±42.8	1431.6±42.1	1485.1±47.4	1472.0±53.4	0.698	0.957
Vitamin C(mg)	78.3±1.4	76.2±1.4	76.6±1.6	75.6±1.8	0.597	0.275
Vitamin E(mg)	34.7±0.6	34.3±0.6	33.8±0.7	33.9±0.8	0.744	0.351
Calcium(mg)	369.8±5.3	371.8±5.3	371.5±5.9	375.9±6.7	0.903	0.484
Sodium (mg)	4545.9±79.2	4563.3±78.7	4681.6±88.5	4634.7±99.2	0.612	0.318
Iron(mg)	23.2±0.3	23.6±0.3	23.6±0.4	24.2±0.4	0.184	0.044
Magnesium(mg)	321.9±3.1	322.8±3	316.8±3.4	320.2±3.9	0.547	0.460
Dietary fiber(g)	11.7±0.2	11.3±0.2	11.2±0.2	11.5±0.2	0.058	0.419
Cholesterol(mg)	238.7±6.3	264.0±6.2**	267.8±7.0**	266.6±7.8*	0.002	0.004
Foods[b]						

For peer review only - http://bmjopen.bmj.com/site/about/guidelines.xhtml

Cereals(g)	453.9±4.9	443.1±4.9	437.1±5.5*	440.8±6.2	0.083	0.058
Rice(g)	234.5±4.7	233.9±4.6	223.9±5.2	229.8±5.9	0.376	0.294
Wheat(g)	159.5±4.7	153.7±4.6	157.2±5.2	155.8±5.8	0.812	0.741
Coarse cereals(g)	16.5±1.4	16.0±1.4	14.1±1.6	12.7±1.8	0.267	0.051
Tubers(g)	37.3±1.9	39.1±1.9	32.9±2.1	32.7±2.4	0.048	0.030
Nuts(g)	3.2±0.5	3.5±0.5	3.4±0.5	4.1±0.6	0.599	0.207
Beans(g)	17.0±0.9	17.3±0.9	17.3±1.1	19.5±1.2	0.362	0.113
Vegetables(g)	335.4±5.6	327.9±5.6	328.7±6.3	320.8±7.1	0.408	0.119
Fruits(g)	54.0±3.2	50.0±3.2	46.9±3.6	48.5±4.1	0.444	0.212
Fish/seafood (g)	30.4±2.1	34.2±2.1	33.8±2.3	42.3±2.6**	0.003	0.001
Meats(g)	90.6±2.9	95.7±2.8	105.7±3.2**	98.4±3.6	0.003	0.016
Abdominal organs (g)	2.9±0.5	3.7±0.5	4.1±0.6	4.4±0.6	0.213	0.050
Eggs(g)	30.8±1.2	33.3±1.2	33.3±1.4	31.4±1.5	0.318	0.749
Milk and dairy products(g)	9.4±1.4	9.7±1.4	11.9±1.6	9.2±1.8	0.576	0.840
Fats_oils(g)	40.7±0.9	40.7±0.9	40.4±1.0	40.1±1.1	0.963	0.608

Values shown are mean ± s.e.

[a]Adjusted for age, alcohol intake, smoking, physical activity, per capita annual income, education level, residence (urban/rural) and geographical regions.

[b]Adjusted for age, energy intake, alcohol intake, smoking, physical activity, per capita annual income, education level, residences (urban/rural) and geographical regions.

[c]Statistical significance was determined by analysis of covariance. Comparisons made with the number of MetS components 0 group,*$P < 0.05$, **$P < 0.01$.

For peer review only - http://bmjopen.bmj.com/site/about/guidelines.xhtml

Table 4 Energy and multivariate adjusted[a,b] mean food and nutrient intake according to the number of metabolic syndrome (MetS) components in women (N=3234)

	Number of MetS components					
	**0**	**1**	**2**	**3–5**	***P*[c]**	**Trend *P***
n,%	837 (25.9%)	906 (28.0%)	770 (23.8%)	721 (22.3%)		
Energy	1972.5±18.8	1966±18.0	1974±19.5	1938.3±20.1	0.549	0.275
Nutrients[a]						
Protein(energy %)	12.7±0.2	12.7±0.2	12.8±0.2	12.8±0.2	0.922	0.567
Fat(energy %)	33.2±0.6	33.4±0.6	33.4±0.7	33.3±0.7	0.979	0.826
Carbohydrate(energy %)	53.9±0.6	53.6±0.6	53.6±0.7	53.7±0.7	0.909	0.715
Nutrients[b]						
Protein(g)	61.0±0.8	61.1±0.8	61.1±0.8	61.4±0.8	0.943	0.583
Beans(protein %)	6.9±0.6	7.2±0.6	7.5±0.6	7.6±0.6	0.449	0.121
Animal foods(protein %)	32.7±1.0	32.3±0.9	32.4±1.0	32.3±1.0	0.929	0.612
Other vegetal food(protein %)	58.6±1.0	59.0±1.0	58.3±1.1	58.5±1.1	0.840	0.695
Fat(g)	72.3±1.4	72.6±1.4	72.5±1.4	72.6±1.5	0.993	0.819
Carbohydrate(g)	259.7±3.2	257.7±3.2	258±3.3	258.5±3.3	0.866	0.689
ß-Carotene(μg)	1578.6±68.0	1391.4±66.9**	1476.5±69.5	1382.7±70.1**	0.001	0.007
Vitamin C(mg)	75.4±2.3	73.9±2.2	76.9±2.3	74.0±2.3	0.292	0.857
Vitamin E(mg)	31.6±0.9	31.9±0.9	32.9±0.9	32.1±0.9	0.309	0.260
Calcium(mg)	353.4±8.4	351.7±8.3	355.5±8.6	342.8±8.7	0.299	0.225
Sodium (mg)	3862.3±116.3	3984.6±114.1	4240.8±118.7**	4005.7±119.0	0.001	0.032
Iron(mg)	19.8±0.5	20.2±0.5	20.2±0.5	19.8±0.5	0.505	0.817
Magnesium(mg)	277.3±4.9	280.9±4.8	278.4±5.0	279.6±5.1	0.804	0.748
Dietary fiber(g)	10.3±0.2	10.6±0.2	10.4±0.2	10.3±0.2	0.555	0.814
Cholesterol(mg)	236.7±10.1	237.6±10.0	243.7±10.3	245.9±10.4	0.640	0.219
Foods[b]						
Cereals(g)	345.3±6.7	347.8±6.5	346.4±6.8	351.6±6.8	0.440	0.335

For peer review only - http://bmjopen.bmj.com/site/about/guidelines.xhtml

Rice(g)	216.4±7.3	207.7±7.1	211.1±7.4	205.5±7.5	0.293	0.139
Wheat(g)	101.9±6.4	106.1±6.3	106.1±6.5	112.6±6.5	0.280	0.066
Coarse cereals(g)	16.1±2.1	15.3±2.1	13.5±2.2	15.8±2.2	0.440	0.646
Tubers(g)	39.8±2.9	38.0±2.9	38.8±3.0	35.2±3.0	0.315	0.112
Nuts(g)	3.7±0.6	3.0±0.6	3.8±0.7	3.4±0.7	0.304	0.907
Beans(g)	15.6±1.4	15.7±1.4	16.6±1.4	16.7±1.4	0.691	0.274
Vegetables(g)	302.2±8.3	303.3±8.1	302.6±8.4	298.2±8.5	0.884	0.583
Fruits(g)	77.9±6.3	76.1±6.2	70.5±6.4	73.5±6.5	0.497	0.268
Fish/seafood (g)	37.3±2.9	34.9±2.8	36.8±2.9	37.1±3.0	0.670	0.886
Meats(g)	86.6±3.7	85.7±3.7	81.0±3.8	84.0±3.8	0.275	0.223
Abdominal organs (g)	3.4±0.7	4.5±0.7	4.1±0.8	4.3±0.8	0.284	0.251
Eggs(g)	28.3±1.8	27.2±1.7	28.6±1.8	27.6±1.8	0.714	0.903
Milk and dairy products(g)	17.8±2.5	14.9±2.4	13.1±2.5	11.5±2.5**	0.020	0.002
Fats_oils(g)	34.7±1.2	35.0±1.2	36.0±1.2	36.0±1.2	0.459	0.133

Values shown are mean ± s.e.

[a]Adjusted for age, alcohol intake, smoking, physical activity, per capita annual income, education level, residence (urban/rural) and geographical regions.

[b]Adjusted for age, energy intake, alcohol intake, smoking, physical activity, per capita annual income, education level, residences (urban/rural) and geographical regions.

[c]Statistical significance was determined by analysis of covariance. Comparisons made with the number of MetS components 0 group,*$P < 0.05$, **$P < 0.01$.

For peer review only

DISCUSSION

Using data from a partially national representative sample of Chinese adults, we found that higher daily intake of meat, fish/seafood, protein, cholesterol, and lower daily intake of coarse cereals, tubers, and dietary fiber were correlated with the number of altered MetS components in men. We also found that higher daily intake of wheat and sodium, and lower intake of beta carotene, milk, and dairy products was correlated with the number of altered MetS components in women. Rather than using a dietary pattern method of analysis, it might be more accurate to evaluate the effect of the single foods or nutrients on the risk of MetS systematically.[3–6]

Our study found that the increased intake of meat and fish/seafood was positively associated with the number of altered MetS components in the Chinese male population. This correlation is closely related to the rapid evolution of the diet in China, due to the remarkable economic developments and changes in lifestyle since 1980s. During this social revolution, the popular diet pattern shifted from the traditional Chinese food, composed mainly of cereals, vegetables and few animal foods, to the consumption of a western diet (e.g. high energy, high fat, high protein, and low dietary fiber).[31]High energy density in carbohydrate-rich food is evidently linked with high levels of obesity and nutrition-related non-communicable diseases.[32]

Prior findings indicated that meat patterns of Chinese meat consumers were characterized by predominant intake of fatty fresh pork. [33] But the high intake of fats from meat, particularly saturated fat, has been associated with higher plasma lipoprotein levels and higher blood pressure levels.[34] According to the Dietary Reference Intake (DRIs) for Chinese adults, general recommendations propose a total fats intake of 20%–30% of the daily caloric consumption with an emphasis on unsaturated fat; saturated fat should represent <10% of total ingested calories. In opposition to these general recommendations, our study showed that intake of fats (% of energy) was slightly higher than the recommended value in both genders (e.g. 31.7% in men, and 33.2% in women with zero altered MetS components; 31.9% in men, and 33.3% in women with three or more altered MetS components).Furthermore, our previous study revealed that, over the past two decades, adults in China consumed increasingly high cholesterol amounts deviating from the recommended intake level. [35]However, a positive correlation between cholesterol intake and the number of MetS components was only found in men in our study. In contrast, other recent studies have indicated that the effect of dietary cholesterol on the incidence of cardiovascular disease and on the level of serum cholesterol is still unclear. [36 37] Thus, compared with a total fat intake, this observation suggests that

For peer review only - http://bmjopen.bmj.com/site/about/guidelines.xhtml

the consumed fats type and cholesterol may be latent factors influencing the risk of elevated MetS components in men.

Intake of dietary protein also influences the MetS components, through the percent of protein from various foods, or through quality supplementation of a specific amino acids.[38] A review of epidemiologic evidence indicated that intake of dietary animal proteins appears to increase the risk of MetS.[39] Gadgil MD et al. found that a diet high in animal protein was associated with higher BMI, higher WC, higher total cholesterol, and higher low density lipoprotein cholesterol in South Asians living in the United States.[40] According to the DRIs for Chinese adults, recommendations for daily protein intake are 65g for men and 55g for women.[41] Our study showed that protein intake both in men and women was also somewhat higher than the recommended amount (e.g. 70.4g and 61.0g per day in men and women, respectively, with zero altered MetS components, and 73.4g and 61.4g per day in men and women, respectively, with three or more altered MetS components). Interestingly, a positive correlation was observed in men between the intake of dietary protein, the percent of protein ingested from animal food (protein %), and the number of MetS or alterations of its components. This observation indicated that men and women may have different sources and different composition of dietary protein, and that in men, a diet rich in animal protein possibly had an important relationship with the MetS components.[38]

Park et al. reported that elevated serum ferritin levels were independently associated with future development of MetS, during a 5year follow-up period.[42] Additionally, a previous study found that serum ferritin levels were higher in men than in women in Chinese adults, and described a positive association between ferritin levels and the prevalence of MetS in both men and women.[43] Similar to prior findings, after adjusting for potential confounding factors, we found a positive correlation between meat intake and an increased number of altered MetS components only in men. This finding indicated that a higher iron intake in men was possibly related to the number of MetS components.

High cereal fiber content and low glycemic index are inherent attributes of most whole-grain foods.[3]Coarse cereals and tubers can provide rich dietary fiber, less fat, and less protein than animal food.[12] Similarly to a study of middle-aged and elderly Japanesepeople,[6] we found that dietary fiber intake had a marginally significant difference among altered MetS components groups 0, 1, 2 and 3-5 overall. Other data indicated that the protective role of fiber from cereals was higher than that of fiber from other sources against the development of MetS and type 2 diabetes.[3] Therefore, an inverse

For peer review only - http://bmjopen.bmj.com/site/about/guidelines.xhtml

association between coarse cereals and tubers and the number of MetS components in our study may be partly explained by dietary fiber. Magnesium is another component of whole grains that may improve insulin sensitivity.[5] However, dietary magnesium intake in both genders was not correlated with the number of altered MetS components in the present study.

Interestingly, our study showed a marginally significant positive correlation between wheat intake and the number of MetS components only in women, after adjusting for potential confounding factors. In China, wheat and rice belong to dietary patterns with different characteristics; wheat tends to be a major component of a modern high-wheat dietary pattern, as mentioned earlier.[7–10] One explanation for this correlation is that the refining wheat process before cooking results in the removal of healthy wheat constituents(e.g. fiber, vitamins, minerals, phytonutrients, and essential fatty acids).[44] Thus, higher carbohydrate consumption (e.g. higher intake of highly refined starch and sugar) could have adverse effects on the profile of metabolic risk[45]. In addition, high glycemic index foods (such as noodles, bread and steamed bread) can induce inflammation, increase oxidant activities, and cause rapid fluctuations of blood glucose and insulin levels.[46] The other explanation for this correlation is that females tend to have a high intake of refined carbohydrates, most of which is prepared by wheat with high levels of salt.[9 10] A stronger positive correlation was observed between sodium intake and the number of altered MetS components in women, which may reflect female dietary habits. High sodium diet not only elevated blood pressure, but also reduced insulin sensitivity.[47]

In keeping with previous studies, [6 48–50] our data showed that the intake of beta carotene, as well as of milk and dairy products was negatively associated with the number of altered MetS components in women. Carotenoids, mainly found in fruit and fresh vegetables, can capture free radicals, quenching singlet oxygen, and improve the protective capability of antioxidants.[51] Milk, rich in calcium and vitamin D, could accelerate the elimination of abdominal fat.[49] More recently, the Chinese Nutrition Society recommended a daily intake of fruits of 200 g to 350 g per capita per day, and a daily intake of milk or its products of 300g per capita per day for the general Chinese population.[52] In contrast to these recommendations, the mean dietary intake of fruits and milk and dairy products was very low for men and women (e.g. 54.0g or 77.9g of fruit per day in men and women, respectively, with zero altered MetS components, and 9.4g and 17.8g milk and dairy products per day in men and women, respectively, with zero altered MetS components). Therefore,

For peer review only - http://bmjopen.bmj.com/site/about/guidelines.xhtml

our study suggests that increasing the intake of beta carotene, milk and dairy products could possibly be of great potential to prevent MetS, or the alteration of its components, in the Chinese population, especially in women.

Fruits, vegetables, whole grains, fish, nuts, and dairy products are six kinds of food that may be beneficial for preventing MetS in various dietary patterns, such as the Mediterranean dietary patter, dietary approaches to stop hypertension, and the Nordic diet.[53] It is well known that the Mediterranean dietary pattern as important features, represented by the daily intake of whole-grain, fruits, vegetables, and dairy products, as well as the weekly intake of fish, poultry, nuts, and beans.[54] Similar to previous multiethnic reports, our study showed a downward trend between the intake of fruits and vegetables and the number of altered MetS components, but not with statistical significance for either men or women. However, in our study a higher intake of fish/seafood (e.g. fish, shrimp, crab, and shellfish) was positively correlated with an increased number of altered MetS components in men. This inconsistency may possibly be due to the existence of an “alcohol” dietary pattern, characterized by animal foods (e.g. meat, fish/seafood), alcohol, and nuts in the Chinese male population,[12] by the fact that in China, fish/seafood is preserved with high levels of salt, or by the fact that the intake of fried dough with soy-bean milk is a typical breakfast for Chinese people[11].

This study has several limitations. First, there maybe residual confounding factors in our study. For instance, considerable recent progress has been made in the identification of genetic loci that are associated with insulin resistance and MetS[55].MetS has been related to the increasing prevalence of obesity, which is escalating even among older people[56].Hormone therapy improves the lipid and oxidative alterations that occur in MetS in postmenopausal women[57].Cumulative alcohol consumption may be an especially important confounding factor. Our previous study found that an “alcohol” dietary pattern was present in Chinese men.[12] In this study, the overall prevalence of alcohol consumption was 64.9% in men and 9.2% in women with three or more altered MetS components. Moderate alcohol intake has been consistently associated with a lower risk of fatal and non-fatal cardiovascular disease. Wine is an important component of the Mediterranean diet,[44] but heavy drinking was positively associated with MetS and with alterations of its components.[58]Due to lack of relevant information, important factors mentioned above were excluded from analysis, which may limit the strength of our findings. Second, the mixed intake of food and nutrients may create more complex interactive effects than the analysis of the intake of foods and nutrients as indicators.[14]

For peer review only - http://bmjopen.bmj.com/site/about/guidelines.xhtml

For instance, prior findings have shown that the “Green Water” dietary pattern, which is characterized by high intake of rice, vegetables, and moderate intake of animal foods, has beneficial effects on MetS or on its components.[7–9] There was no significant negative correlation between the intake of rice and vegetables and the number of altered MetS components in both genders in the present study.

Third, in this study, we measured diet using a combination of three consecutive days by 24-hour dietary recall, and a household inventory. This method has a few shortcomings. One is that the allocation of oil and other condiments among household members was based on the proportion of reference men, instead of total food intake, which could lead to an additional level of error for the analysis of individual intake. Another shortcoming is that dietary data collected by this method omits adjustment for intake of oil and other condiments for foods eaten away from home[20]. Additionally, it is unclear whether short-term records of dietary recall can fully reflect the long-term intake of a dietary pattern.[59]

Fourth, despite a progressive increase or decrease in intake levels of some foods and nutritional factors (e.g. meat, protein, cholesterol in men and beta carotene, milk, and dairy products in women) according to the cumulative distribution of MetS components, it is uncertain whether these levels would have a direct impact on MetS and their metabolic profiles. Possible reasons for this uncertainty is due to our cross-sectional design, and we could not come to any causal conclusion to explain the relationships between dietary factors and the number of altered MetS components.

Fifth, although some correlations were observed between dietary factors and the number of altered MetS components among Chinese adults aged 18–75 years in the present study, it is difficult to predict an overall and long-term associations between dietary factors and the number of altered MetS components for Chinese adult population. One the one hand, our study is just a partial national representative sample including only nine provinces of China, and prior correlation findings have not previously been reported to the best of our knowledge; One the other hand, gradual increase in the level of urbanization has led to improvement in the dietary intake of Chinese adult population over the past decade. It is necessary to conduct multicenter studies that represent the groups of interest in different regions and periods, and thereby extend the applicability of such dietary factors in the battle against MetS in China.

For peer review only - http://bmjopen.bmj.com/site/about/guidelines.xhtml

CONCLUSIONS

In conclusion, our study showed that some foods and nutritional factors are correlated to the number of altered MetS components among the Chinese adult population aged 18–75 years. However, whether consumption of these foods and nutritional factors would directly affect metabolic disorders requires further investigation; further prospective and clinical research is needed to examine these associations.

For peer review only

Contributors: MWC, BZ conceived the study. MWC, YFOY and WWD completed all statistical analyses and interpretation of data. MWC drafted the manuscript. ZHW and WWD contributed to the discussion. HJW and BZ revised the manuscript.

Funding: The work was supported by the National Institutes of Health(NIH: R01-HD30880, DK056350 and R01-HD38700).

Competing interests: None.

Patient consent: Obtained.

Ethics approval: All protocols, instruments and the process for obtaining informed consent for this study were approved by the Ethical Committee of the University of North Carolina at Chapel Hill and the National Institute for Nutrition and Food Safety of USA, Chinese Center for Disease Control and Prevention. Signed consent forms were obtained from all participants.

Data sharing statement: The data used for the analysis are available from the CPC at http://www.cpc.unc.edu/projects/china.

REFERENCES

1. Ahluwalia N, Andreeva VA, Kesse-Guyot E, *et al.*Dietary patterns, inflammation and the metabolic syndrome. *Diabetes Metab* 2013;39:99–110.
2. Xi B, He D, Hu Y, *et al*. Prevalence of metabolic syndrome and its influencing factors among the Chinese adults: The China Health and Nutrition Survey in 2009.*Prev Med* 2013;57: 867–71.
3. McKeown NM, Meigs JB, Liu S, *et al*. Carbohydrate nutrition, insulin resistance, and the prevalence of the metabolic syndrome in the Framingham Offspring Cohort. *Diabetes Care*

For peer review only - http://bmjopen.bmj.com/site/about/guidelines.xhtml

2004; 27:538–46.

4. Ford ES, Giles WH, Dietz WH. Prevalence of the metabolic syndrome among US adults: findings from the third National Health and Nutrition Examination Survey. *JAMA* 2002; 287: 356–59.

5. Evangelopoulos AA,Vallianou NG, panagiotakos DB, *et al*. An inverse relationship between cumulating components of the metabolic syndrome and serum magnesium levels. *Nutr Res* 2008;28:659–63.

6. Rei Otsuka1,Tomoko Imai1,Yuki Kato1,*et al*. Relationship between number of metabolic syndrome components and dietary factors in middle-aged and elderly Japanese subjects. *Hypertens Res* 2010;33:548-54.

7. Li Y, He Y, Lai J, *et al*. Dietary patterns are associated with stroke in Chinese adults. *J Nutr* 2011;141:1834–39.

8. Wang D, He Y, Li Y, *et al*. Dietary patterns and hypertension among Chinese adults: a nationally representative cross-sectional study. *BMC Public Health* 2011;11:925.

9. He Y, Li Y, Lai. Dietary patterns as compared with physical activity in relation to metabolic syndrome among Chinese adults. *Nutrition, Metabolism & Cardiovascular Diseases* 2013;23:920-28.

10. Batis C, Sotres-Alvarez D, Gordon-Larsen P, *et al*. Longitudinal analysis of dietary patterns in Chinese adults from 1991 to 2009. *Br J Nutr* 2014;111:1441–51.

11. Batis C,Mendez MA, Gordon-Larsen P, *et al*. Using both principal component analysis and reduced rank regression to study dietary patterns and diabetes in Chinese adults. *Public Health Nutr* 2016;19:195-203.

12. Zhang JG. Changes in dietary patterns and their associations with general and central obesity among adults in China (1991-2009).PhD Thesis, Chinese Center for Disease Control and Prevention, 2013.

13. Mishra GD, McNaughton SA, Bramwell GD, *et al*. Longitudinal changes in dietary patterns during adult life.*Br J Nutr* 2006;96:735-44.

14. Hu FB. Dietary pattern analysis: A new direction in nutritional epidemiology. *CurrOpin Lipidol* 2002;13:3–9.

15. Kant AK. Dietary patterns and health outcomes. J*ournal of the American Dietetic Association*

For peer review only - http://bmjopen.bmj.com/site/about/guidelines.xhtml

2004;104:615-35.

16. Ma G, Kong L(editor). The report of 2002 China National Nutrition and Health Survey (Part Nine)—behavior and way of life. Beijing: People's Medical Publishing House,2006.
17. Popkin BM, Du S, Zhai F, *et al.* Cohort profile: the China health and nutrition survey-monitoring and understanding socioeconomic and health change in China, 1989–2011. *Int. J. Epidemiol* 2010;39:1435–40.
18. Zhang JG, Wang HJ, Wang YF, *et al.* Dietary patterns and their associations with childhood obesity in China. *British Journal of Nutrition* 2015; 113:1978-84.
19. Zhang JG, Wang ZH, Wang HJ, *et al.* Dietary patterns and their associations with general obesity and abdominal obesity among young Chinese women. *European Journal of Clinical Nutrition* 2015; 69:1009-14.
20. Zhai FY, Guo XG, Popkin BM, *et al.* Evaluation of the 24-hour individual recall method in China. *Food and Nutrition Bulletin* 1996; 17:154–61.
21. Yang Y, Wang G, Pan X (editor). China food composition table. 2nd edition. Beijing: Beijing Medical University Press, 2009.
22. Millen BE, Pencina MJ, Kimokoti RW， *et al.* Nutritional risk and the metabolic syndrome in women: opportunities for preventive intervention from the Framingham Nutrition Study. *Am J Clin Nutr* 2006; 84:434–41.
23. Dami˜;ao R, Castro TG, Cardoso MA， *et al.* Dietary intakes associated with metabolic syndrome in a cohort of Japanese ancestry. *Br J Nutr* 2006; 96:532–38.
24. Bell AC, Ge K, Popkin BM. Weight gain and its predictors in Chinese adults. *Int J Obes* 2001; 25:1079–86.
25. WHO. Physical Status: The Use and Interpretation of Anthropometry. Report of a WHO Expert Committee. World Health Organization, Geneva, 1995.
26. Chobanian AV, Bakris GL, Black HR， *et al.* The seventh report of the Joint National Committee on prevention, detection, evaluation, and treatment of high blood pressure: the JNC 7 report. *JAMA* 2003;289:2560–72.
27. Batis C,Mendez MA,Sotres-Alvarez D， *et al.* Dietary pattern trajectories during 15 years of follow-up and HbA1c, insulin resistance and diabetes prevalence among Chinese adults. *J Epidemiol Community Health* 2014;68:773–79.

For peer review only

For peer review only - http://bmjopen.bmj.com/site/about/guidelines.xhtml

28. Yan S, Li J, Li S, *et al*. The expanding burden of cardio metabolic risk in China: the China Health and Nutrition Survey. *Obes. Rev* 2012;13:810–21.

29. Albertci KG, Eckel RH, Grundy SM, *et al*. Harmonizing the metabolic syndrome: a joint interim statement of the international diabetes federation task force on epidemiology and prevention; national heart, lung, and blood institute; American heart association; world heart federation; international atherosclerosis society; and international association for the study of obesity. *Circulation* 2009;20:1640–45.

30. Ainsworth BE, Haskell WL, Whitt MC, *et al*. Compendium of physical activities: an updateof activity codes and MET intensities. *Med Sci Sports Exerc* 2000;32: S498-S504.

31. Du S, Lu B, Zhai F, *et al*. A new stage of the nutrition transition in China. *Public Health Nutr* 2002; 5:169-74.

32. Popkin BM. The nutrition transition: An overview of world patterns of change. *Nutr Rev* 2004;62:S140-S143.

33. Wang ZH, Zhai FY, Wang HJ, *et al*. Secular trends in meat and seafood consumption patterns among Chinese adults, 1991–2011.*European Journal of Clinical Nutrition* 2015; 69: 227–33

34. Woo1 HD, Shin A, Kim J. Dietary patterns of Korean adults and the prevalence of metabolic syndrome: A cross-sectional study. *PLoS One* 2014;9:e111593.

35. Su C, Jia XF, Wang ZH, *et al*. Trends in dietary cholesterol intake among Chinese adults: a longitudinal study from the China Health and Nutrition Survey, 1991–2011. *BMJ Open* 2015; 5:e007532.

36. Samantha B, Gowri R, Rohini V, *et al*.Dietary cholesterol and cardiovascular disease: a systematic review and meta-analysis. *Am J Clin Nutr* 2015;102:276–94.

37. Fatemeh RK, Gangadaran S, Amy G, *et al*. The role of dietary cholesterol in lipoprotein metabolism and related metabolic abnormalities: a mini-review. *Critical Reviews in Food Science and Nutrition* 2016; 56:2408–15.

38. Andersen JC, Fernandez ML. Dietary strategies to reduce metabolic syndrome. *Reviews in Endocrine & Metabolic Disorders* 2013; 14,241-54.

39. Amanda B, Terry C, Christine M. Dietary patterns and metabolic syndrome-a review of epidemiologic evidence. *Asia Pac J Clin Nutr* 2006;15:134-42.

40. Gadgil MD, Anderson CA, Kandula NR. Dietary patterns are associated with metabolic risk

For peer review only - http://bmjopen.bmj.com/site/about/guidelines.xhtml

factors in south Asians living in the United States. *J Nutr* 2015;145:1211-17.

41. Chinese Nutrition Society(editor).Chinese Dietary Reference Intakes (2013 edition).Beijing: Science Press,2014.
42. Park SK, Ryoo JH, *et al*. Association of serum ferritin and the development of metabolic syndrome in middle-aged Korean men: A 5-year follow-up study. *Diabetes Care* 2012;35, 2521–26.
43. Li J, Wang R, Luo D. Association between serum ferritin levels and risk of the metabolic syndrome in Chinese adults: A population study. *PLoS One* 2013;8:e74168.
44. Dario G, Antonio C, Katherine E.The effects of diet on inflammation: Emphasis on the metabolic syndrome. *J Am Coll Cardiol* 2006;48:677–85.
45. Noakes M, Keogh JB, Foster PR, *et al*. Effect of an energy-restricted, high-protein, low-fat diet relative to a conventional high-carbohydrate, low-fat diet on weight loss, body composition, nutritional status, and markers of cardiovascular health in obese women. *Am J Clin Nutr* 2005;81:1298–306.
46. Avignon A, Hokayem M, Bisbal C, *et al*. Dietary antioxidants: do they have a role to play in the ongoing fight against abnormal glucose metabolism? *Nutrition* 2012;28:715–21.
47. Lastra G, Dhuper S, Johnson MS, *et al*. Salt, aldosterone, and insulin resistance: impact on the cardiovascular system. *Nat Rev Cardiol* 2010;7:577–84.
48. Czernichow S, Vergnaud AC, Galan P, *et al*. Effects of long-term antioxidant supplementation and association of serum antioxidant concentrations with risk of metabolic syndrome in adults. *Am J Clin Nutr* 2009;90:329–35.
49. Sébédio JL, Corinne MB. Metabolic syndrome and dairy product consumption: Where do we stand?. *Food Research International* 2016; 89:1077–84.
50. Kim J. Dairy food consumption is inversely associated with the risk of the metabolic syndrome in Korean adults. *J Hum Nutr Diet* 2013;26,Suppl 1:S171–S179.
51. Sun C, Ling W, Huang G, *et al*(editor).Nutrition and food hygiene.7th edition. Beijing: People's Medical Publishing House, 2013.
52. Chinese Nutrition Society (editor).Chinese Dietary Guide. Beijing: People's Medical Publishing House, 2016.
53. Calton EK, James AP, Pannu PK, *et al*. Certain dietary patterns are beneficial for the metabolic

For peer review only - http://bmjopen.bmj.com/site/about/guidelines.xhtml

syndrome: reviewing the evidence. *Nutr Res* 2014; 34: 559–68.

54. Schwingshack L, Hoffmann G. Does a Mediterranean-type diet reduce cancer risk? *Curr Nutr Rep* 2016; 5: 9–17.

55. Brown AE, Mark W. Genetics of insulin resistance and the metabolic syndrome. *Curr Cardiol Rep* 2016; 18: doi 10.1007/s11886-016-0755-4.

56. Dominguez LJ, Mario B. The biology of the metabolic syndrome and aging. *Curr Opin Clin Nutr Metab Care* 2016; 19:5-11.

57. Sánchez-Rodríguez MA, Mariano ZF, Castrejón-Delgado L, *et al*. Effects of hormone therapy on oxidative stress in postmenopausal women with metabolic syndrome. *Int. J. Mol. Sci* 2016; 17: 1388, doi:10.3390/ijms17091388.

58. Im HJ, Park SM, Choi JH, *et al*. Binge drinking and its relation to metabolic syndrome in Korean adult men. *Korean J Fam Med* 2014;35:173-81.

59. Ranil J. Comparison dietary assessment methods in Sri Lankan adults: use of 24-hour dietary recall and 7-day weighed intake.*BMC Nutrition* 2016; 18: doi 10.1186/s40795-016-0059-5.

For peer review only

For peer review only - http://bmjopen.bmj.com/site/about/guidelines.xhtml

Supplemental Table 1. The full list of food items and examples for each food group using by a household inventory

No	Food group	Examples of food items
1	Rice	Japonica rice, glutinous rice
2	Flour	Steamed bread, noodles
3	Coarse cereals	Corn, barley, millet
4	Tubers	Potato, sweet potato
5	Beans	Beans, mung beans,
6	Vegetables,	Tomato, cabbage, white radish, mungbean sprout
7	Fruits	Apples,watermelons
8	Nuts	Peanuts, melon seeds
9	Pork	
10	Other livestock meat	Cattle, sheep
11	Poultry meat	Chicken, duck, goose
12	Abdominal organs	Pork liver, pork heart
13	Fish/seafood	Fish, shrimp, crab
14	Milk and dairy products	Milk, yogurt
15	Eggs	Eggs, duck eggs
16	Vegetable oil	
17	Fat oil	
18	Snacks and dessert	Bean jelly, cold noodles, cakes
19	Fans/starch and sugar	Starch, sugar
20	Condiments	Salt, soy sauce, sauce

For peer review only - http://bmjopen.bmj.com/site/about/guidelines.xhtml

Supplemental Table 2. Energy and multivariate adjusted[a,b] mean nutrient intake according to the number of metabolic syndrome (MetS)components in men (N=2800)

	Number of MetS components					
	**0**	**1**	**2**	**3–5**	P^b	Trend P
n，%	866（30.9%）	832（29.7%）	618（22.1%）	484（17.3%）		
Energy	2349.9±20.8	2317.5±21.3	2296.7±24.6	2269.6±27.8	0.109	0.016
Nutrients[a]						
VitA(μg RE)	445.8±17.76	443.9±17.58	440.4±19.83	451.1±22.29	0.986	0.886
$VitB_1$(mg)	1.0±0.01	1.0±0.01	1.1±0.02	1.0±0.02	0.688	0.656
$VitB_2$(mg)	0.8±0.01	0.8±0.01	0.8±0.01	0.8±0.01	0.777	0.510
$VitB_3$(mg)	15.1±0.20	15.4±0.19	15.6±0.22	15.6±0.25	0.262	0.101
K(mg)	1750.2±16.31	1740.1±16.15	1727.0±18.25	1753.1±20.47	0.711	0.955
Zn(mg)	11.7±0.10	11.8±0.10	11.9±0.11	12.0±0.13	0.273	0.053
Se(μg)	48.1±0.9	48.2±0.89	48.8±1.00	49.2±1.13	0.858	0.402
P(mg)	1060.9±8.07	1063.2±7.99	1063.4±9.01	1067.7±10.13	0.959	0.596
Mn(mg)	7.0±0.19	7.0±0.19	6.7±0.21	7.3±0.24	0.409	0.701
Cu(mg)	2.1±0.03	2.2±0.03	2.1±0.04	2.2±0.04	0.149	0.141

Values shown are mean ±s.e.

[a]Adjusted for age, energy intake, alcohol intake, smoking, physical activity, per capita annual income, education level, residence(urban/rural) and geographical regions.

[b]Statistical significance was determined by analysis of covariance.

Compared with Number of MetS components 0 group,*P＜0.05, **P＜0.01.

Supplemental Table 3. Energy and multivariate adjusted[a,b] mean nutrient intake according to the number of metabolic syndrome (MetS)components in women (N=3234)

	Number of MetS components					
	0	1	2	3–5	P^b	Trend P
n，%	866（30.9%）	832（29.7%）	618（22.1%）	484（17.3%）		
Energy	2349.9±20.8	2317.5±21.3	2296.7±24.6	2269.6±27.8	0.109	0.016
Nutrients[a]						
VitA(μg RE)	476.2±31.68	452.7±31.12	463.2±32.27	444.9±32.5	0.665	0.333
$VitB_1$(mg)	0.8±0.02	0.9±0.02	0.9±0.02	0.9±0.02	0.412	0.165
$VitB_2$(mg)	0.7±0.02	0.7±0.02	0.7±0.02	0.7±0.02	0.826	0.601
$VitB_3$(mg)	13.4±0.27	13.4±0.26	13.4±0.27	13.5±0.27	0.999	0.868
K(mg)	1605.5±25.61	1603.7±25.20	1608.4±26.14	1582.2±26.31	0.612	0.353
Zn(mg)	10.1±0.15	10.3±0.15	10.2±0.15	10.3±0.15	0.455	0.421
Se(μg)	38.1±0.78	39.2±0.77	38.9±0.80	39.0±0.80	0.312	0.270
P(mg)	900.6±12.84	916.0±12.62	910±13.08	915.1±13.18	0.429	0.284
Mn(mg)	5.5±0.20	5.7±0.19	5.8±0.20	5.6±0.20	0.384	0.575
Cu(mg)	1.8±0.04	1.9±0.04	1.9±0.04	1.9±0.04	0.325	0.132

Values shown are mean ±s.e.

[a]Adjusted for age, energy intake, alcohol intake, smoking, physical activity, per capita annual income, education level, residence(urban/rural) and geographical regions.

[b]Statistical significance was determined by analysis of covariance.

Compared with Number of MetS components 0 group,*P<0.05, **P<0.01.

For peer review only - http://bmjopen.bmj.com/site/about/guidelines.xhtml

STROBE Statement—checklist of items that should be included in reports of observational studies

	Item No	Recommendation
Title (page 1 line 2–8)	1	(*a*) Indicate the study's design with a commonly used term in the title or the abstract
Abstract (page2 line 12–55)		(*b*) Provide in the abstract an informative and balanced summary of what was done and what was found
**Introduction** (page 3 line 29–page 5 line 4)		
Background/rationale<br>(page 3 line 30–page 4 line 50)	2	Explain the scientific background and rationale for the investigation being reported
Objectives<br>(page 4 line 55–page 5 line 2)	3	State specific objectives, including any prespecified hypotheses
**Methods** (page 5 line 6–page 8 line 31)		
Study design(page 5 line 7–14)	4	Present key elements of study design early in the paper
Setting(page 5 line 16–22)	5	Describe the setting, locations, and relevant dates, including periods of recruitment, exposure, follow-up, and data collection
Participants(page 5 line 24–33)	6	(*a*) *Cohort study*—Give the eligibility criteria, and the sources and methods of selection of participants. Describe methods of follow-up<br>*Case-control study*—Give the eligibility criteria, and the sources and methods of case ascertainment and control selection. Give the rationale for the choice of cases and controls<br>*Cross-sectional study*—Give the eligibility criteria, and the sources and methods of selection of participants
This is a cross-sectional study studies		(*b*) *Cohort study*—For matched studies, give matching criteria and number of exposed and unexposed<br>*Case-control study*—For matched studies, give matching criteria and the number of controls per case
Variables<br>(page 6 line 18–19, page 6 line 57–page 7 line 48, page 8 line 12–20)	7	Clearly define all outcomes, exposures, predictors, potential confounders, and effect modifiers. Give diagnostic criteria, if applicable
Data sources/ measurement<br>(page 5 line 39–page 6 line 16,<br>page 6 line 23–page 7 line 16,page 7 line 19—48)	8*	For each variable of interest, give sources of data and details of methods of assessment (measurement). Describe comparability of assessment methods if there is more than one group
Bias<br>(page 5 line 11–18, page 5 line 24–48,<br>page 6 line 6–8,page 6 line 37–40,<br>page 8 line 15–19)	9	Describe any efforts to address potential sources of bias
Study size<br>(page 5 line 24–33)	10	Explain how the study size was arrived at
Quantitative variables<br>(page 7 line 32–48, page 8 line 7–10)	11	Explain how quantitative variables were handled in the analyses. If applicable, describe which groupings were chosen and why

For peer review only

For peer review only - http://bmjopen.bmj.com/site/about/guidelines.xhtml

Statistical methods	12	(*a*) Describe all statistical methods, including those used to control for confounding	(page 8 line 6–page 8 line 31)
		(*b*) Describe any methods used to examine subgroups and interactions	This paper is unconcerned with interactions.
		(*c*) Explain how missing data were addressed	(page 5 line 26)
		(*d*) *Cohort study*—If applicable, explain how loss to follow-up was addressed<br>*Case-control study*—If applicable, explain how matching of cases and controls was addressed<br>*Cross-sectional study*—If applicable, describe analytical methods taking account of sampling strategy	(page 8 line 6–27)
		(*e*) Describe any sensitivity analyses	None

Continued on next page

For peer review only

For peer review only - http://bmjopen.bmj.com/site/about/guidelines.xhtml

**Results**(page 8 line 38–page 10 line 40)		
Participants (This is a cross–sectional study studies)	13*	(a) Report numbers of individuals at each stage of study—eg numbers potentially eligible, examined for eligibility, confirmed eligible, included in the study, completing follow-up, and analysed
		(b) Give reasons for non-participation at each stage
		(c) Consider use of a flow diagram
Descriptive data (page 8 line 37–page 9 line 2)	14*	(a) Give characteristics of study participants (eg demographic, clinical, social) and information on exposures and potential confounders
		(b) Indicate number of participants with missing data for each variable of interest
		(c) *Cohort study*—Summarise follow-up time (eg, average and total amount)
Outcome data (page 9 line 7–20)	15*	*Cohort study*—Report numbers of outcome events or summary measures over time
		Case-control study—Report numbers in each exposure category, or summary measures of exposure
		Cross-sectional study—Report numbers of outcome events or summary measures
Main results (page 9 line 27–page 10 line 40) This paper is unconcerned with relative risk.	16	(*a*) Give unadjusted estimates and, if applicable, confounder-adjusted estimates and their precision (eg, 95% confidence interval). Make clear which confounders were adjusted for and why they were included
		(*b*) Report category boundaries when continuous variables were categorized
		(*c*) If relevant, consider translating estimates of relative risk into absolute risk for a meaningful time period
Other analyses (None)	17	Report other analyses done—eg analyses of subgroups and interactions, and sensitivity analyses
**Discussion**(page 18 line 3–page 23 line 2)		
Key results(page 18 line 4–15, page 22 line 52–page 23 line 2)	18	Summarise key results with reference to study objectives
Limitations (page 21 line 38–page 22 line 47)	19	Discuss limitations of the study, taking into account sources of potential bias or imprecision. Discuss both direction and magnitude of any potential bias
Interpretation(page 18 line 17–page 21 line 36)	20	Give a cautious overall interpretation of results considering objectives, limitations, multiplicity of analyses, results from similar studies, and other relevant evidence
Generalisability (page 22 line 33–48 , page 22 line 53–page 23 line 2)	21	Discuss the generalisability (external validity) of the study results
**Other information**(page 23 line 4–26)		
Funding (page 23 line 9–10)	22	Give the source of funding and the role of the funders for the present study and, if applicable, for the original study on which the present article is based

*Give information separately for cases and controls in case-control studies and, if applicable, for exposed and unexposed groups in cohort and cross-sectional studies.

Note: An Explanation and Elaboration article discusses each checklist item and gives methodological background and published examples of transparent reporting. The STROBE checklist is best used in conjunction with this article (freely available on the Web sites of PLoS Medicine at http://www.plosmedicine.org/, Annals of Internal Medicine at http://www.annals.org/, and Epidemiology at http://www.epidem.com/). Information on the STROBE Initiative is available at www.strobe-statement.org.

For peer review only - http://bmjopen.bmj.com/site/about/guidelines.xhtml
